# Single-cell transcriptome reveals cellular hierarchies and guides p-EMT-targeted trial in skull base chordoma

**DOI:** 10.1038/s41421-022-00459-2

**Published:** 2022-09-20

**Authors:** Qilin Zhang, Lijiang Fei, Rui Han, Ruofan Huang, Yongfei Wang, Hong Chen, Boyuan Yao, Nidan Qiao, Zhe Wang, Zengyi Ma, Zhao Ye, Yichao Zhang, Weiwei Wang, Ye Wang, Lin Kong, Xuefei Shou, Xiaoyun Cao, Xiang Zhou, Ming Shen, Haixia Cheng, Zhenwei Yao, Chao Zhang, Guoji Guo, Yao Zhao

**Affiliations:** 1 Department of Neurosurgery, Huashan Hospital, Shanghai Medical College, Fudan University, Shanghai, China; 2 National Center for Neurological Disorders, Huashan Hospital, Shanghai Medical College, Fudan University, Shanghai, China; 3 Center for Stem Cell and Regenerative Medicine, Zhejiang University School of Medicine, Hangzhou, Zhejiang China; 4 Department of Oncology, Huashan Hospital, Shanghai Medical College, Fudan University, Shanghai, China; 5 Department of Pathology, Huashan Hospital, Shanghai Medical College, Fudan University, Shanghai, China; 6 Department of Plastic and Reconstructive Surgery, Shanghai Institute of Precision Medicine, Shanghai Ninth People’s Hospital, Shanghai Jiao Tong University School of Medicine, Shanghai, China; 7 Department of Radiology, Huashan Hospital, Shanghai Medical College, Fudan University, Shanghai, China; 8 Department of Radiation Oncology, Shanghai Proton and Heavy Ion Center, Fudan University Cancer Center, Shanghai, China; 9 State Key Laboratory of Medical Neurobiology and MOE Frontiers Center for Brain Science, Institutes of Brain Science, Fudan University, Shanghai, China; 10 Shanghai Key Laboratory of Brain Function Restoration and Neural Regeneration, Shanghai, China; 11 Neurosurgical Institute of Fudan University, Shanghai, China; 12 National Clinical Research Center for Aging and Medicine, Huashan Hospital, Fudan University, Shanghai, China

**Keywords:** Bone cancer, Cancer genomics, Targeted therapies

## Abstract

Skull base chordoma (SBC) is a bone cancer with a high recurrence rate, high radioresistance rate, and poorly understood mechanism. Here, we profiled the transcriptomes of 90,691 single cells, revealed the SBC cellular hierarchies, and explored novel treatment targets. We identified a cluster of stem-like SBC cells that tended to be distributed in the inferior part of the tumor. Combining radiated UM-Chor1 RNA-seq data and in vitro validation, we further found that this stem-like cell cluster is marked by cathepsin L (*CTSL*), a gene involved in the packaging of telomere ends, and may be responsible for radioresistance. Moreover, signatures related to partial epithelial–mesenchymal transition (p-EMT) were found to be significant in malignant cells and were related to the invasion and poor prognosis of SBC. Furthermore, YL-13027, a p-EMT inhibitor that acts through the TGF-β signaling pathway, demonstrated remarkable potency in inhibiting the invasiveness of SBC in preclinical models and was subsequently applied in a phase I clinical trial that enrolled three SBC patients. Encouragingly, YL-13027 attenuated the growth of SBC and achieved stable disease with no serious adverse events, underscoring the clinical potential for the precision treatment of SBC with this therapy. In summary, we conducted the first single-cell RNA sequencing of SBC and identified several targets that could be translated to the treatment of SBC.

## Introduction

Skull base chordomas (SBC) are rare primary malignant bone tumors arising from the notochordal remnant tissue in the clivus or sellar region, with an incidence of 0.3 cases per million individuals^1^. Although chordoma is generally considered a slow-growing tumor, it is characterized by local invasion and a high recurrence rate^2^. Thus, complete resection (R0 resection) is the aim of an optimal surgical procedure^3^. Unfortunately, it is often precluded in SBC by the tumor location and the surrounding critical structures, such as the brainstem and optic pathways, leaving most patients (almost 80%) with residual tumor tissue and inevitable recurrence^4,5^.

Additionally, the resistance of SBC to radiotherapy, chemotherapy and targeted therapies is quite common, subjecting patients to repeated recurrence and surgery before death. Radiotherapy with a dosage higher than 70 Gy is strongly recommended due to the high radioresistance rate of chordoma. However, there is an inevitable dilemma for SBC since only moderate radiation doses (< 55 Gy) can be applied to accommodate the tolerance of dose-limiting structures such as the brainstem, optic nerves, and temporal lobes^3,6,7^. Chemotherapy is regarded as an inappropriate option for SBC therapy^1,3^. In recent decades, evidence for the utilization of targeted therapies has emerged from several phase II clinical trials. The main targets assessed were *KIT*, *PDGFR*, *EGFR*, *HER2*, and regulators of angiogenesis, such as *VEGFR*^8,9^. Immune checkpoint blockade therapy was also considered a potential second-line choice since *PD-1/PD-L1* was detected in chordoma. However, the presence of *PD-1/PD-L1* in SBC tumor cells or the tumor microenvironment (TME) is still under debate^10,11^. The resistance of SBC to these nonsurgical therapies may result from the presence of cancer stem cells (CSCs). However, no robust evidence or biomarkers of CSCs have been identified in SBC.

Recent advances in single-cell techniques have provided an avenue to explore genetic and functional heterogeneity at cellular resolution. Single-cell RNA sequencing (scRNA-seq) studies of human tumors have revealed new insights into the tumor composition and intrinsic mechanisms of CSCs, the TME, and tumor invasion^12–14^.

To elucidate the presence of CSCs and *PD-1/PD-L1*-expressing subpopulations in SBC and to further explore the cell heterogeneity and the mechanism of tumor aggressiveness, we performed single-cell transcriptomic analysis, profiled 90,691 single-cell transcriptomes in twelve samples from six patients, and identified several novel potential therapeutic markers for prognosis prediction and targeted therapy.

## Results

### A single-cell expression atlas of SBC

**Fig. 1:**
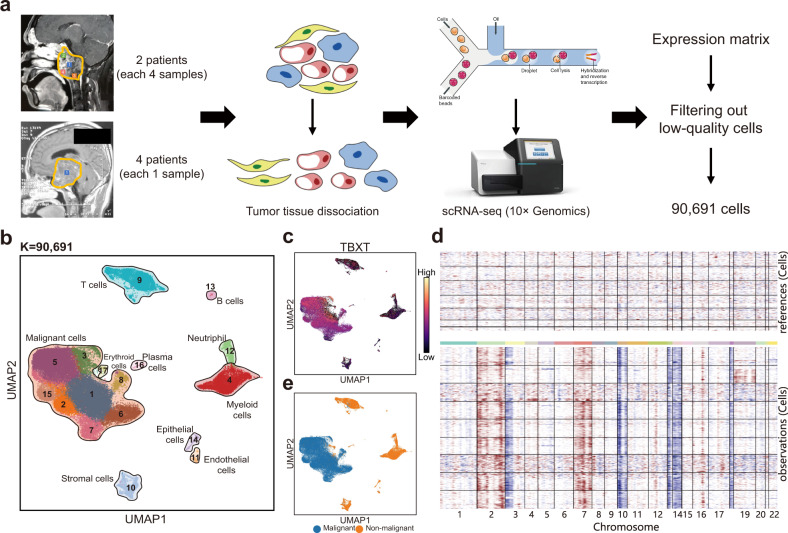
Study design and identification of malignant cells by single-cell RNA-seq. **a** Workflow showed the collection and processing of fresh SBC samples for scRNA-seq. Four samples were obtained in the anterior, posterior, inferior and central regions of SBC from two patients, while one sample was obtained from each of the other four patients. **b** The UMAP of all 90,691 cells passed RNA QC from 12 samples of six patients. Cells were clustered into 17 clusters (marked by numbers). The 17 clusters were classified into 10 subtypes (marked by the text in black), including one malignant and nine nonmalignant groups according to canonical gene markers. **c** UMAP plot of *TBXT*, a known marker gene for SBC malignant cells. **d** Heatmap showed large-scale CNVs for individual cells (rows) from a representative tumor (Patient 1), inferred based on the average expression of 100 genes surrounding each chromosomal position (columns). Red: amplifications; blue: deletions. **e** UMAP plot shows the distributions of malignant and nonmalignant cells based on CNVs and malignant cell markers.

We performed droplet-based scRNA-seq on the 10× Genomics Chromium platform for cells dissociated from twelve samples obtained from six patients. None of the patients had previously received radiotherapy. Four samples were obtained from Patients 1 and 2, representing different regions of the tumor, while one sample was obtained from the other four patients (Fig. 1a and Supplementary Fig. S1a, Table S1).

Single-cell transcriptome data of 90,691 cells were retained after initial quality control (Fig. 1a, b and Supplementary Fig. S1b). The mean gene number and mean UMI number of the six patients involved in our scRNA-seq data were 2,322 and 10,748, respectively. We distinguished ~60,000 malignant and ~30,000 nonmalignant cells in total through the following process. First, we performed cluster analysis on each sample separately. Then, we considered manually annotated immune clusters, stromal clusters, and epithelial clusters as putative nonmalignant clusters to define a reference group^15–17^. Compared with other clusters, which were considered putative malignant clusters, putative nonmalignant clusters were checked for lower gene expression levels of chordoma tumor cell markers (*TBXT, S100A1*, and *VIM*) according to the published literature (Fig. 1c and Supplementary Fig. S1c)^12,18,19^. Both putative nonmalignant clusters and malignant clusters were utilized for CNV inference. Reference groups (putative nonmalignant clusters) were adjusted based on the primary inferred CNV results and then used in the second round of analyses. Finally, malignant clusters were determined according to the CNV pattern (Fig. 1d, e and Supplementary Fig. S1d–h).

Although the CNVs of most malignant cells exhibited amplification in chromosomes 2 and 7, we found an extra difference in CNVs among the six patients and identified several subclones. For example, the CNVs of both Patients 1 and 6 exhibited loss in chromosomes 10, 13, 14, and 18, while amplification of chromosome 12 was observed only in Patient 2 (Fig. 1d, e and Supplementary Fig. S1d–h).

### Transcriptome profiles of the TME in SBC

**Fig. 2:**
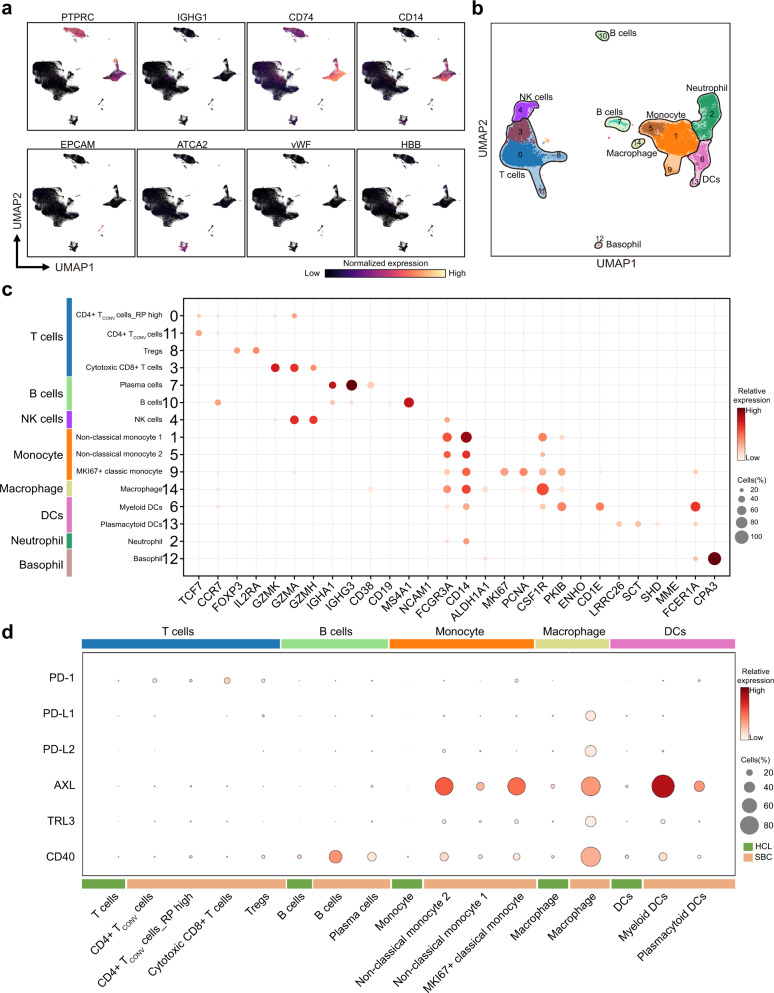
Expression heterogeneity of nonmalignant cells in the SBC ecosystem. **a** UMAP plot of each selected canonical marker genes for identifying the clusters. **b** Fifteen immune cell clusters (marked by numbers) can be partitioned into eight sub-clusters (marked by the text in black) according to the known markers. **c** Dot plot shows the expression of known marker genes in these eight immune cell sub-clusters. **d** Comparison of SBC-infiltrated and HCL immune cells in *PD-1, PD-L1, PD-L2*, and multiple *PD-L1* regulators.

Single-cell profiles of nonmalignant cells highlighted the composition of the TME in SBC. We partitioned the ~30,000 nonmalignant cells into nine main clusters by their expression profiles (Fig. 1b). We annotated clusters by the expression of canonical marker genes of T cells, B cells, plasma cells, neutrophils, myeloid cells, erythroid cells, epithelial cells, endothelial cells, and stromal cells (Figs. 1b, 2a and Supplementary Table S2). Each of the clusters contained cells from different patients, indicating that cell types and expression states in the TME are largely consistent across SBC and do not represent patient-specific subpopulations or batch effects, although they do vary in their proportions (Fig. 2b). We found further diversity within both stromal cells and immune cells (T cells, B cells, plasma cells, neutrophils, and myeloid cells) through finer clustering, powered by their relatively large numbers in our dataset.

The ~3,700 stromal cells were partitioned into four main subsets (Supplementary Fig. S2a, b and Table S3). One subset expressed classical markers of myofibroblasts (clusters 3, 4, and 5), including alpha smooth muscle actin (*ACTA2*) and myosin light-chain proteins (*MYLK*, *MYL9*; Supplementary Fig. S2b, c). Myofibroblasts are an established component of the TME and have been linked to wound healing and contracture^20^. A second subset (cluster 0) expressed receptors, ligands, and extracellular matrix (ECM) genes, including *PDGFRA* and *PDGFRL*, which have been associated with cancer-associated fibroblasts (CAFs, Supplementary Fig. S2d)^21^. Cluster 1 is characterized by high ribosome protein expression (Supplementary Fig. S2e). The fourth subpopulation (cluster 2) can be defined as fibroblast-like cells (*APOD* and *CYP1B1*; Supplementary Fig. S2f)^22^. Through CellphoneDB, we found that cancer cells were relatively likely to interact with stromal cells (Supplementary Fig. S3a, b), and the significantly enriched ligand‒receptor pairs included *FN1*, collagen, and the TGFβ family (Supplementary Fig. S3c).

The main immune cell cluster was partitioned into eight cell types (Fig. 2c, d and Supplementary Tables S4, S5), which we annotated as T cells (clusters 0, 3, 8 and 11), B cells (clusters 7 and 10), NK cells (cluster 4; *NCAM1 (CD56), FCGR3A (CD16)*, *GNLY*, *KLRF1*, *KLRD1*), monocytes (clusters 1, 5 and 9), macrophages (cluster 14; low *CD14*, low *FCGR3A*, *CSF1R*), neutrophils (cluster 2; *MME(CD10), CEACAM8(CD66b)*), dendritic cells (DCs, clusters 6 and 13), and basophils (cluster 12; *FCER1A, CPA3*)^23,24^.

The monocyte subsets were defined as non-classic monocytes (clusters 1 and 5; *CD14, FCGR3A (CD16), ALDH1A1, NRG1, LGALS1, ANXA2, CST3*) and MKI67 + classic monocytes (cluster 9; *CD14*, low *FCGR3A(CD16), MKI67, PCNA*). The DC subsets included myeloid DCs (cluster 6; *PKIB, ENHO, CD1E, CD1B, CD1C*) and plasmacytoid DCs (cluster 13; *LRRC26, SCT, SHD, PTCRA*). The T-cell subsets were classified as regulatory T cells (Tregs) (cluster 8; *FOXP3, IL2RA(CD25)*), conventional CD4^+^ T helper cells (CD4^+^ T_CONV_) (clusters 0 and 11; *TCF7, CCR7, CD48, CD40L, CD69*) and cytotoxic CD8^+^ T-cell populations (CD8^+^ T) (cluster 3; *CD8A, CD8B, GZMK, GZMA, GZMH*). The B-cell subsets were divided into plasma cells (cluster 7; *IGHA1, IGHG3, CD38, SDC1, FCRL5, MZB1*) and B cells (cluster 10; *CD19, MS4A1(CD20), VPREB3*) (Fig. 2c, d and Supplementary Tables S4,S5). Some ligand‒receptor pairs were significantly enriched between cancer cells and T cells, such as the TNF family and *ICAM1* (Supplementary Fig. S4a).

To explore whether the immunotherapy approach was applicable in SBC, we compared the tumor-infiltrated and human cell landscape (HCL) immune cells (Fig. 2d and Supplementary Fig. S4b)^24^. One of the highly expressed genes in infiltrated myeloid DCs was *AXL*, a key PD-L1 protein regulator capable of increasing PD-L1 protein expression^25–27^. As reported, DCs could potentially interact with multiple T-cell subsets via PD-1/PD-L1, leading us to compare the expression of *PD-1/PD-L1* and its regulators in all immune cells^28^. Compared to the same type of immune cell in the HCL, the level of *PD-L1 (CD274)* was significantly elevated in T cells, monocytes, macrophages, and neutrophils (Supplementary Fig. S4b). Moreover, infiltrated macrophages showed higher levels of multiple *PD-L1* regulators, including *AXL, TLR3*, and *CD40* (Supplementary Fig. S4b). Our data suggested that PD-1/PD-L1 therapy might be a practical therapeutic approach in SBC (Supplementary Fig. S4c).

### Landscape of the expression heterogeneity of malignant cells in SBC

**Fig. 3:**
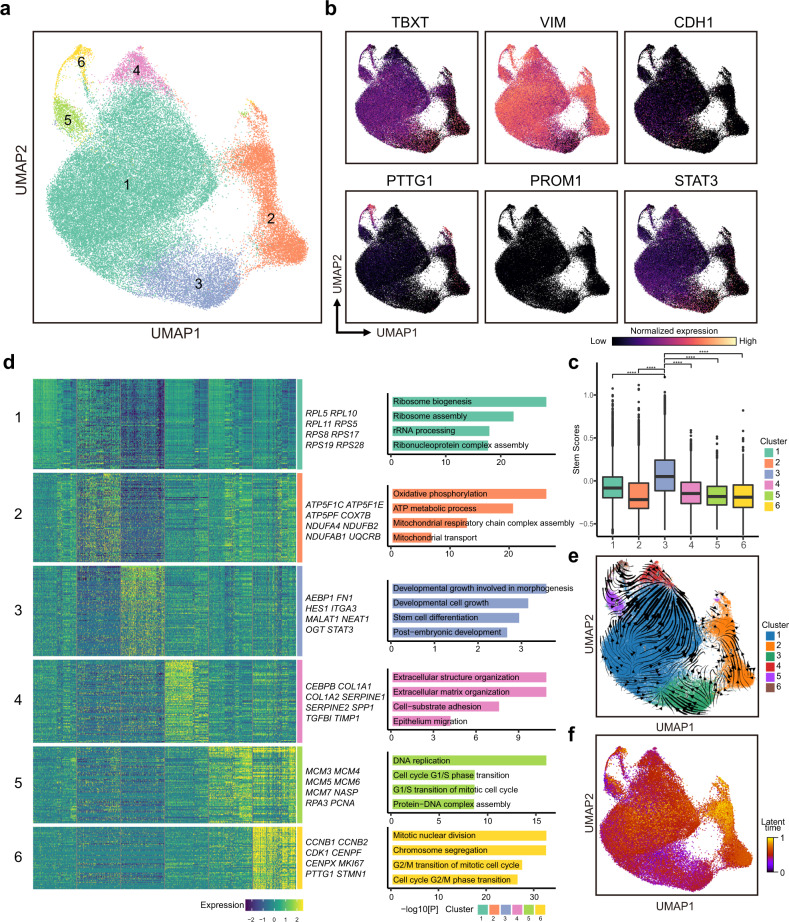
Expression heterogeneity and stem-like malignant cells in SBC. **a** 60,626 malignant cells were clustered into six clusters using unbiased clustering. **b** The UMAP plot of the expression of SBC subpopulation and cancer stem-cell markers. **c** Stem score was calculated in each malignant cluster. Cluster 3 showed the highest stem score. ^****^P-value ≤ 0.0001. **d** The heatmap showed the markers and pathway enrichment results of each SBC malignant cluster. Genes with high expression levels were labeled on the right side of the heatmap. **e**, **f** The RNA velocity trajectory and the latent time of SBC malignant cells.

We next sought to determine the heterogeneity of malignant cells in SBC. To this end, we focused on the ~60,000 malignant cells and identified six subtypes of malignant cells (Fig. 3a and Supplementary Table S6). Each cluster contained cells from all six patients, and all six clusters expressed SBC markers (e.g., *TBXT, KRT19*, and *S100A1*, Fig. 3b and Supplementary Fig. S5a, b). In addition, all six malignant cell clusters highly expressed the mesenchymal cell signature (*VIM, CDH2*; Fig. 3b and Supplementary Fig. S5c), and one (cluster 1) also expressed several epithelial markers, such as *CDH1* (Fig. 3b). We also identified two subpopulations (clusters 5 and 6) with proliferative signatures, including *PTTG1, CENPX*, and *STMN1* (Fig. 3b and Supplementary Fig. S5d).

The presence of CSCs in chordoma was strongly suspected due to the resistance of chordoma to chemotherapy and radiotherapy^29^. However, several studies also identified markers in cell lines or in vitro culture, including *PROM1 (CD133)*, *FUT4 (CD15)*, *SOX2*, *SOX9*, *CXCR4*, and *CD24*^30–32^. Therefore, we further explored the subpopulations of malignant cells characterized by CSC markers in SBC. To our surprise, several markers, such as *PROM1, CXCR4*, and *SOX2*, were not highly expressed in any clusters, while others (*SOX9* and *CD24*) were expressed across all clusters (Fig. 3b and Supplementary Fig. S5e, f, Table S6).

To identify the cluster with stem-cell characteristics among SBC malignant cells, a series of gene markers summarized as CSC markers in a variety of invasive bone tumors (including chordoma, osteosarcoma, chondrosarcoma, Ewing’s sarcoma, multiple myeloma, and giant cell tumor)^30,33^ were used as an initial gene set to define stem-like cells. Correlation analysis of these genes in our chordoma scRNA-seq dataset was performed. Ten genes, including *CD44, MYC, KLF4, ICAM1, ALCAM, NT5E, TBXT, STAT3, FUT4*, and *SOX9*, had significant correlation (Supplementary Fig. S5g). Therefore, we used these ten genes as a gene set to calculate the stem score of each cluster and found that cluster 3 showed a significantly higher stem score (Fig. 3b, c and Supplementary Fig. S5h). In addition, the markers of cluster 3 were also enriched for stem-cell related pathways, including stem-cell differentiation and developmental cell growth (Fig. 3d). Velocity analysis suggested a potential progenitor role of cluster 3 (Fig. 3e, f). Accordingly, we proposed cluster 3 as the stem-like cell cluster of SBC.

CSCs were found to be related to tumor radioresistance in previous studies^29^. To investigate whether cluster 3 was responsible for the radioresistance of SBC, we performed in vitro irradiation of UM‑Chor1, one of the SBC cell lines. After 64 Gy radiation and two-day culture, we identified 118 genes that were upregulated at the transcriptomic level (Supplementary Table S7). Based on the expression pattern similarity in our scRNA-seq data, we identified three modules (Supplementary Fig. S6a, b and Table S8): Module 1 was enriched for DNA damage/telomere stress-induced senescence and was considered to reflect postradiation stress. Module 3 was enriched for the regulation of cell‒cell adhesion, which was related to both postradiation stress and radioresistance^34–36^. Module 2 was associated with the packaging of telomere ends, which was reported to be related to the radioresistance of tumors in multiple cancers^37,38^. By single-cell signature scoring, we found that Module 2 was significantly upregulated in cluster 3, suggesting the presence of Module 2 in preradiated chordoma and possible responsibility for radioresistance (Supplementary Fig. S6c).

To further verify this hypothesis, we looked into the molecules involved in the packaging of the telomere ends pathway. Among them, cathepsin L (*CTSL*) is one of the most widely studied genes in radioresistance^39^. *CTSL* had high expression in both cluster 3 and postradiated UM-Chor1 cells (Supplementary Fig. S6b, d). We then knocked down CTSL expression level by siRNA in UM-Chor1 cells. The survival rate of CTSL knockdown cells was significantly decreased compared with that of control cells after 64 Gy radiation (Supplementary Fig. S6e). Z-FY-CHO, one of the CTSL inhibitors^40^, was also applied to UM-Chor1, and the results corresponded with the *CTSL* knockdown assay (Supplementary Fig. S6e). In addition, no significant difference in survival rate was observed in UM-Chor1 between 64 Gy radiation group and 32 Gy radiation plus Z-FY-CHO group (Supplementary Fig. S6f). These results suggested that targeting *CTSL*, a molecule involved in the packaging of the telomere ends pathway, may improve radiation efficacy in SBC treatment.

### Spatial distribution of SBC subpopulations

To gain further insight into cellular heterogeneity across the spatial distribution, we obtained four samples from the anterior, posterior, inferior, and central regions of SBC from two patients under intraoperative neuro-navigation (Supplementary Fig. S1a). We found no common difference in cellular composition between the central and noncentral positions (Supplementary Fig. S7a, b). Differentially expressed gene (DEG) analysis revealed that *ID3, COL1A2, MTRNR2L2, and MTRNR2L1* were elevated in the central region of SBC (Supplementary Fig. S7c). The UMAP of all cancer cells from Patient 2 showed a cluster consisting almost entirely of cells from the central location (51 cells from the central and one cell from the posterior), and we defined this cluster as the central-specific cluster (Supplementary Fig. S7d). The upregulated genes in the central-specific cluster were enriched in bone development related pathways, including ossification and osteoblast differentiation (Supplementary Fig. S7e). The mean single-cell scores of these pathways were elevated in the central regions of both patients (Supplementary Fig. S7f, g). These findings indicated that the bone development related pathway, rather than the stem cell or radioresistant pathway, is more likely to occur in the central area of SBC. We also analyzed the spatial distribution of stem-like cells (cluster 3 in Fig. 3) in Patients 1 and 2, and the inferior part was found to contain the highest proportion of stem-like cells (Patient 1: 38%, Patient 2: 44%, Supplementary Fig. S7h). Thus, we suggest that surgeons remove as much as possible of the inferior portion of the tumor to improve the effect of postoperative radiotherapy.

### Intratumoral expression heterogeneity of the malignant compartment

**Fig. 4:**
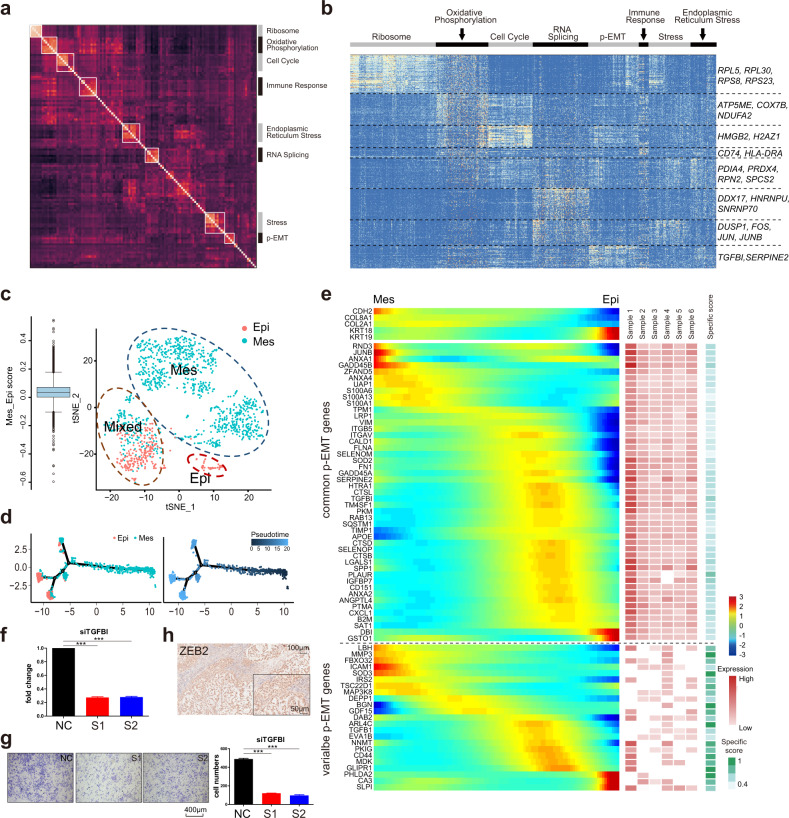
Unbiased clustering reveals a common program of p-EMT in SBC. **a** Heatmap depicted pairwise correlations of 120 intratumoral programs derived from six patients. Eight meta-signatures reflected intratumoral programs. **b** Heatmap showed differentially expressed genes (rows) identified by non-negative matrix factorization (NNMF) clustered by their expression across single cells (columns) from a representative tumor (Patient 1). The gene clusters reveal intratumoral programs that are differentially expressed in Patient 1. The corresponding gene signatures were numbered, and selected genes were indicated (right). **c** The 1,374 cells identified as the Mes-type or the Epi-type were clustered and visualized. **d** Single-cell trajectories and the variations of gene pseudotime were visualized. **e** Heatmap showed NNMF gene scores aggregated by samples common (top rows) and tumor-specific (bottom rows) genes within the p-EMT program. **f**, **g** Knockdown of TGFBI by siRNA attenuated the invasiveness of UM-chor1. The results of quantification were shown on the right side. S1, S2: two different siRNA. NC: normal control. Scale bar, 400 μm, ^***^P-value ≤ 0.001. **h** IHC demonstrated that the expression of ZEB2 was involved in the p-EMT program of SBC.

We then aimed to identify expression patterns that varied among each patient’s malignant cells. We used non-negative matrix factorization (NNMF) to uncover coherent sets of genes that were preferentially co-expressed by subsets of malignant cells. We first defined gene signatures that varied among malignant cells from each donor. Then, we distilled eight meta-signatures that reflected common expression programs that varied within multiple samples (Fig. 4a and Supplementary Table S9). The high concordance between signatures from different patients suggests that they reflect common patterns of intratumoral expression heterogeneity in SBC. The levels of eight common expression programs in Patient 1 are shown in Fig. 4b.

Eight programs were preferentially expressed in subsets of malignant cells from at least two patients (Fig. 4a, b and Supplementary Table S10). One program (cluster 1) mainly consisted of ribosomal proteins reflecting ribosome biogenesis. Another program (cluster 5) included *CALR, MYDGF*, and *TMED9*, suggesting the involvement of the endoplasmic reticulum. Cluster 6 was enriched for RNA binding and splicing genes. These three clusters indicate RNA translation and protein synthesis in SBC. Cluster 2 was enriched for oxidative phosphorylation genes. Another two programs reflected the cell cycle (cluster 3) and apoptosis (cluster 4) in each tumor. The seventh program consisted of *JUN, FOS*, and immediate early genes implicated in cellular activation and stress responses.

A final expression program contained genes associated with ECM and had features of EMT. This program was evident in subsets of cells from five of the six patients examined (Fig. 4a and Supplementary Table S10).

### A p-EMT program in SBC

Although EMT programs have been widely considered potential drivers of invasion, drug resistance, and radioresistance, their patterns and roles in human chordoma remain unclear. We further calculated the EMT scores of each malignant cell based on the KS method^41^. The cells that showed significantly increased EMT scores were identified as the Mes-type, while the cells that showed significantly decreased EMT scores were identified as the Epi-type. The 1,374 typical Mes-type and Epi-type cells were mainly classified into 3 clusters: Mes-type cells, Epi-type cells, and Mixed-type cells (Fig. 4c). The pseudotime analysis showed a cell transition trajectory from Mes-type to Epi-type (Fig. 4d). We closely examined the pseudotime related genes and ECM program for features of EMT. In addition to ECM genes such as matrix metalloproteinases, collagen, and integrin, this program included EMT markers (*CD44, VIM, ITGB5, SERPINE2*, and *TIMP1*; Fig. 4e and Supplementary Table S10). One of the top-scoring genes in this program was transforming growth factor (TGF)-β-induced (*TGFBI*), implicating the classic EMT regulator TGF-β. Moreover, knockdown of TGFBI expression using siRNA attenuated the invasiveness of UM‑Chor1 cells (Fig. 4f, g). We further detected the expression of classical EMT transcription factors (TFs). *ZEB1, TWIST1/2*, and *SNAIL1/2* were not expressed; only *ZEB2* was detected by scRNA-seq and immunohistochemistry (IHC) (Fig. 4h).

While epithelial markers were detected in this program, mesenchymal markers were still maintained, suggesting an intermediate state of EMT in SBC. EMT is increasingly recognized as a continuous and variable process^42,43^. We suggest that the in vivo program identified here reflects a partial EMT-like state or ‘p-EMT.’

### Investigation of the invasiveness of p-EMT cells in vitro

**Fig. 5:**
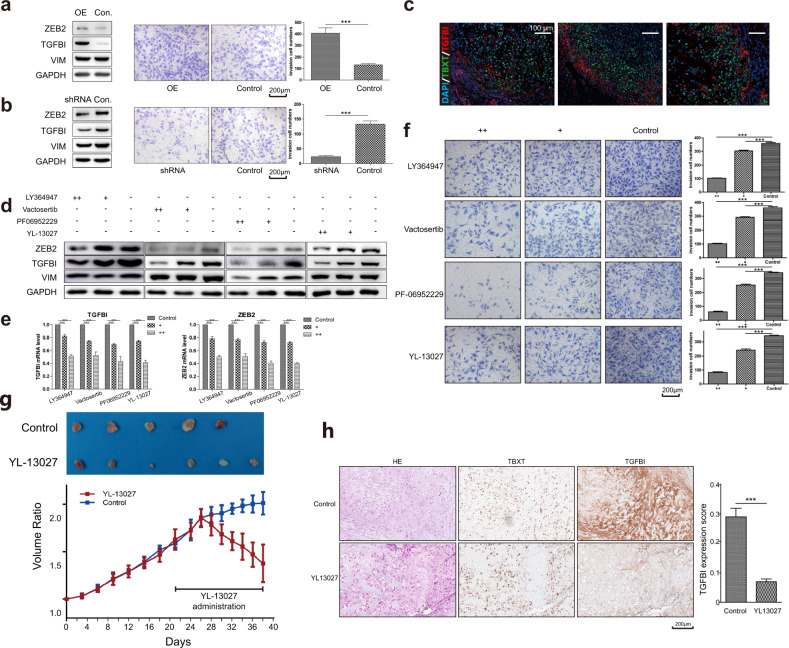
p-EMT is a novel target to SBC. **a**, **b** The construction of stable UM-chor1 cell line with TGFBI overexpression (OE) and knockdown (shRNA). The protein level of TGFBI consists with that of ZEB2. Overexpression of TGFBI increased the invasiveness of the UM-Chor1 through the p-EMT program, and vice versa. The results of quantification were shown on the right side. Scale bar, 200 μm. ^***^P-value ≤ 0.001. **c** IF images of representative SBC tumors stained for p-EMT markers TGFBI and the malignant cell-specific marker TBXT. Scale bar, 100 μm. **d**, **e** Four TGF-βR1 inhibitors (LY364947, Vactosertib, PF06952229, and YL-13027) repressed the p-EMT-like program, including the decrease of ZEB2 and TGFBI in both protein and mRNA levels. ^***^P-value ≤ 0.001. **f** Four TGF-βR1 inhibitors could attenuate the invasiveness of UM-Chor1, suggesting p-EMT as a drug target in vitro. The results of quantification were shown on the right side. Scale bar, 200 μm. ^***^P-value ≤ 0.001; ^**^P-value ≤ 0.01, ^*^P-value ≤ 0.05. **g** YL-13027 treatment resulted in a significant reduction in the growth of UM-Chor1 xenograft. The administration of YL-13027 started on day 21. **h** The HE and IHC showed TGFBI was significantly decreased after YL-13027 treatment in vivo. The expression score of TGFBI was calculated by Qupath and was shown on the right side. Scale bar, 200 μm. ^***^P-value ≤ 0.001.

We investigated the relationship between the p-EMT program and invasiveness in the UM‑Chor1 cell line, which also expressed p-EMT markers such as *TGFBI*. We generated stable cell lines with TGFBI overexpression (OE) and knockdown (shRNA) (Fig. 5a, b). Overexpression of TGFBI increased the invasiveness of UM‑Chor1 cells through the p-EMT program, as indicated by the upregulation of ZEB2. Conversely, knockdown of TGFBI decreased the invasiveness of cells (Fig. 5b).

### p-EMT cells localize to the leading edge in situ

Our in vitro functional data suggested that the p-EMT program was associated with the invasiveness of SBC cells, which led us to investigate the in situ spatial localization of cells expressing this program within SBC tumors. We performed immunofluorescence staining of TGFBI, representing the expression of p-EMT program, along with the SBC marker *TBXT*. This experiment revealed a population of SBC cells with a highly expressed p-EMT pattern, characterized by *TGFBI* expression and cell localization to the leading edge of the tumor in close apposition to the surrounding stroma (Fig. 5c).

### p-EMT is a drug target in vitro and in vivo

To further confirm the functional significance and assess the targetability, we inhibited p-EMT activation in UM‑Chor1 cells and primary cultures of SBC cells by treatment with TGF-βR1 inhibitors (LY364947, Vactosertib, PF06952229, YL-13027) for 48 h^44,45^. The high (++) and low (+) concentrations of LY364947, Vactosertib, and YL13027 were 100 µM and 10 µM, respectively. The high (++) and low (+) concentrations of PF06952229 were 25 µM and 10 µM, respectively. The treatment decreased the invasiveness of the cell line and repressed the p-EMT program, marked by a decrease in ZEB2 and TGFBI at both the protein and mRNA levels, which was the same as the responses caused by genetic inactivation of *TGFBI* (Fig. 5d–f).

To further verify the p-EMT inhibition in vivo, YL-13027 was administered to UM‑Chor1 xenograft models with subcutaneous tumors from day 21. YL-13027 treatment resulted in a significant reduction in the growth of UM‑Chor1 xenografts compared with vehicle treatment (Fig. 5g). Furthermore, HE and IHC staining of the xenograft tumors showed a decrease in the TGFBI expression score following YL-13027 treatment (Fig. 5h). Together, these findings demonstrate that p-EMT activation, which could be inhibited by YL-13027, is a novel drug target for SBC.

### Target searching based on HCL also highlights p-EMT

The construction of the HCL triggered our interest in exploring the DEGs, which were high in SBC but low in other tissues at the single-cell level. After comparing the two databases, we obtained a list of highly expressed genes in SBC*. TBXT, S100A10*, and *S100A1* were the pathological markers of chordoma (Supplementary Fig. S8a). Correlation analysis revealed that these top 20 genes belong to one network along with their most relevant genes in SBC. Among them, selenoprotein family members and RP genes may play essential roles.

Combined with genes previously explored in SBC, we found unexpectedly that there were 10 genes involved in p-EMT (Supplementary Fig. S8a). Coincidentally, *SELENOM* and *S100A1* also contribute to malignancy (Supplementary Fig. S8b, c). Although they were difficult to target directly, we also noted the interacting genes, which might also be potential targets (Supplementary Fig. S8d, e).

### p-EMT predicts recurrence and other clinical features

**Fig. 6:**
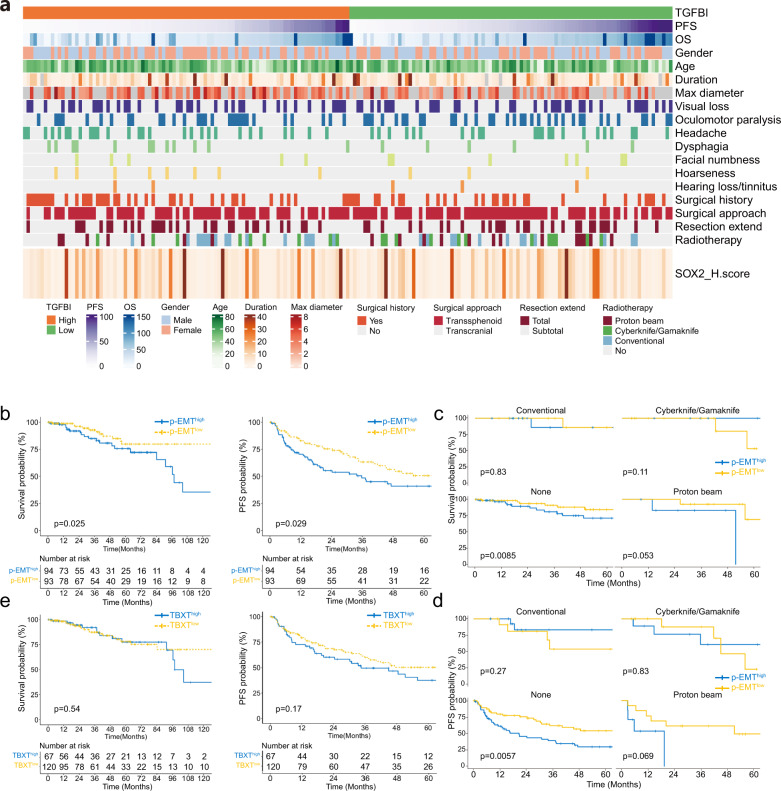
Survival implications of p-EMT and TBXT in SBC. **a** The clinical data of the 187 SBC cohort were summarized. **b** Overall survival (OS) (left) and progression-free survival (PFS) (right) were significantly different between the p-EMT^high^ and p-EMT^low^ cohorts in 187 SBC patients. OS (**c**) and PFS (**d**) of patients with different postoperation radiotherapy. Patients with p-EMT^high^ SBC and no postoperation radiotherapy had significantly lower OS and PFS. **e** OS (left) and PFS (right) showed no significant difference between the TBXT^high^ and TBXT^low^ cohorts in 187 SBC patients.

We next considered the generality and prognostic significance of the p-EMT program in SBC. Between 2010 and 2018, we obtained 187 samples and records from 152 patients, including 120 primary and 67 recurrent SBC patients. The average follow-up time was 40.5 months. There was no difference in several baseline parameters, including age, gender, surgical approach, and total resection rate (Fig. 6a and Supplementary Table S11). Surprisingly, we found that patients who received proton/carbon beam radiosurgery had worse progression-free survival (PFS) than other patients, which may be due to the bias that patients who received proton/carbon beam radiosurgery suffered from more invasive or recurrent SBC (Supplementary Fig. S9a). Using IHC, we investigated the expression levels of TBXT and TGFBI (Supplementary Fig. S9b). The expression score was calculated by Qupath^46^. The expression score of each marker did not differ significantly across years, suggesting a similar distribution of patients with TGFBI and TBXT expression. We classified the samples into p-EMT^high^ and p-EMT^low^ groups according to TGFBI expression. Kaplan‒Meier survival curves showed that patients in the p-EMT^high^ group had significantly lower PFS and higher mortality than patients in the low-risk group (*P* < 0.05), especially those who did not receive radical resection or radiotherapy (Fig. 6b–d). However, there was no significant correlation between TBXT expression and PFS or mortality (Fig. 6e).

### YL-13027 attenuated tumor growth in three SBC patients

**Fig. 7:**
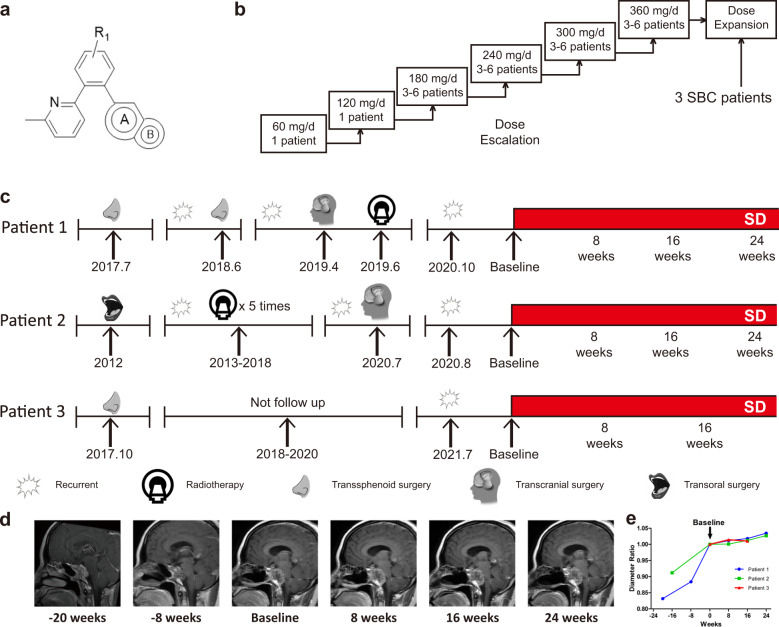
Clinical trial of YL-13027 in three human SBC patients. **a** The chemical structure of YL-13027, a distinct chemical entity as a TGF-βR1 inhibitor. **b** The design of the phase I dose-escalation and dose-expansion study of YL-13027. The starting dose was 60 mg/day and then increased to 120, 180, 240, 300, and 360 mg/day. At each dosage over 120 mg/day, a cohort of 3–6 patients were enrolled, and the dose-limiting toxicities (DLTs) were evaluated within the first 4 weeks of drug administration. Three SBC patients were enrolled at a dose of 360 mg/day. No DLT was observed in these three patients. One patient experienced transient rash in both the knee and abdomen. **c** Diagram of SBC progression and treatment in three patients. The tumor was confirmed to recur locally and progressed rapidly. Up to the cutoff date, all patients achieved stable disease. **d** The tumor (Patient 1) was monitored by MRI evaluation. **e** SBC obviously progressed before enrollment and stabilized after the administration of YL-13027.

To further explore the safety and efficacy of YL-13027 in patients, we translated these findings into the clinic. We enrolled three SBC patients in a single-arm, open-label, single and multiple-dose-escalation phase I clinical study of YL-13027 administered orally to advanced solid tumor patients (Fig. 7a). The design and protocol of the clinical trial are summarized in Supplementary Table S12. Five patients with SBC were assessed, and three were enrolled (Supplementary Fig. S10a and Table S13). All patients were treated with YL-13027 at a dosage of 360 mg/day (180 mg bid) (Fig. 7b). After single-dose treatment, YL-13027 reached a stable plasma concentration (Supplementary Fig. S10b–d). Two patients were treated with YL-13027 continuously for more than 24 weeks, and one was treated for 16 weeks. Up to the study cutoff date, several adverse events (AEs) were observed and are summarized in Supplementary Table S14, including rash and transient creatinine elevation. No serious AEs (SAEs) were observed. The Response Evaluation Criteria in Solid Tumors Version 1.1 (RECIST 1.1) was used to evaluate the treatment outcome. All SBC patients achieved stable disease (SD) (Fig. 7c). Compared with MRI scanning before enrollment, YL-13027 treatment obviously attenuated tumor growth accompanied by symptom relief in all patients (Fig. 7d, e and Supplementary Fig. S10e, f), and non-measurable progression under RECIST 1.1 was detected up to the cutoff date. In this phase I clinical trial, YL-13027 monotherapy showed a good safety profile and retarded tumor growth, demonstrating that targeting p-EMT and thus inhibiting the TGF-β pathway is a novel strategy for SBC treatment.

## Discussion

In recent years, scRNA-seq has facilitated the identification of intratumoral heterogeneity, drug resistance pathways, and immune infiltration patterns relevant to tumor biology, diagnosis, and therapy. To date, our study is the first scRNA-seq study to identify the cellular hierarchies of SBC. We intended to identify the novel treatment targets in SBC for immunotherapy, radiotherapy, and targeted therapy.

PD-1/PD-L1 blockade therapy is one of the most promising approaches in cancer immunotherapy. PD-1/PD-L1 therapy restores the exhausted host antitumor immune responses mediated by tumors and has demonstrated significant clinical efficacy in treating various advanced cancers^47^. With several case reports showing that SBC patients benefited from PD-1/PD-L1 blockade therapy^48,49^, we focused on the expression of markers related to *PD-1/PD-L1* in our research. In our data, compared with HCL, no significant increase in *PD-1/PD-L1* expression was detected in malignant cells. In contrast, the expression of *PD-L1 (CD274)* and its multiple regulators, including *AXL* and *CD40*, was identified in myeloid DCs, macrophages, and TILs, which is consistent with other studies^11,50,51^. Furthermore, the expression level of *PD-L1* in TILs was associated with the prognosis of chordoma^48^. Moreover, a case report showed that metastatic chordoma could be controlled by a PD-1 receptor inhibitor and had a PFS duration of 9.3 months^49^. Our study suggests the possibility of PD-1/PD-L1 inhibitors as second-line therapies for SBC, and subsequent in vivo research remains to be conducted.

Radioresistance is quite common in chordoma and is attributed to CSCs in many malignancies^52^. It is believed that the CSC content and its intrinsic radiosensitivity vary among tumors, affecting the tolerance to radiotherapy^53^. Thus, many studies have speculated on the presence of radioresistant CSCs in chordoma^29,54^. Through clustering and stem score calculation, we identified a subpopulation of SBC malignant cells that exhibited characteristics of CSCs, as stem-like cells. Further analysis showed activation of the packaging of telomere ends pathway in this cluster, which may be responsible for radioresistance. In vitro experiments revealed that it is possible to increase the sensitivity of SBC to radiotherapy by inhibiting this pathway. Therefore, the prognosis of SBC patients may be improved when combining radiotherapy and stem-like cluster targeting drugs. Our findings provide a novel approach to developing radiotherapy sensitizers for SBC.

Our key findings include identifying a p-EMT program in malignant cells. The p-EMT program was previously identified and proven to be associated with metastasis and invasiveness in multiple cancers^55,56^. In SBC, this program involves upregulation of certain epithelial genes and maintenance of mesenchymal genes. Although reminiscent of an EMT-like process, the program lacks classical TFs thought to drive EMT, except for *ZEB2*. In recent years, several EMT markers have been found to be responsible for chordoma invasion and proliferation^57^. In addition, accumulating evidence shows that *ZEB2* plays a pivotal role in EMT-induced malignant mechanisms, including tumor invasion, tumor recurrence^58^, drug resistance^59^, cancer stem-cell-like traits^60^, and metastasis^61^, raising the possibility that *ZEB2*-associated p-EMT is involved in SBC invasiveness. According to our scRNA-seq data, the *ZEB2* expression did not significantly correlate with the p-EMT program in the level of individual tumor cell. However, in the cohort of 187 SBC patients, the *ZEB2* expression did correlate with *TGFBI*, which represented the p-EMT level. In addition, our in vitro experiments also demonstrated that the *ZEB2* expression of UM-Chor1 was significantly downregulated after the application of inhibitors, which targeted *TGFBI*, and the invasiveness of tumor cells was correspondingly attenuated. At the same time, other observations highlight the diagnostic and targeted value of p-EMT in SBC: first, in vitro and in vivo invention demonstrate that the application of p-EMT inhibitor could prevent the growth and invasion of the tumor. Second, SBC patients with high p-EMT levels should receive radiotherapy and more frequent follow-up since they have worse PFS and OS, especially without R0 resection or radiotherapy. Third, based on the comparison between the SBC map and HCL at the single-cell level, we also unexpectedly identified other p-EMT genes, such as *SELENOM*, as new potential drug targets.

We further translated this finding into clinical research. YL-13027, which could deactivate the p-EMT pathway through TGF-βR1 inhibition, could attenuate the tumor growth rate in three SBC patients. Our unbiased definition of an in vivo p-EMT program in SBC could help guide the future treatment of these human bone cancers. In addition, TGF-βR1 inhibitors, such as YL-13027, could block the activation of the p-EMT program and augment PD-1 inhibition to promote T-cell regression in multiple cancers^62^. Thus, combining a TGF-βR1 inhibitor with PD-1 inhibition may be a novel therapeutic strategy for SBC patients.

In summary, our work provides important insights into SBC biology and an atlas of malignant, stromal, and immune cells. Our computational analyses revealed novel treatment prospects in immunotherapy, radiotherapy, and targeted therapy. Finally, we identified a p-EMT program and proved its diagnostic and targeted value. Although further studies are needed, p-EMT and its inhibitor may improve future diagnostic strategies and treatment algorithms.

## Materials and methods

### Study population and human specimens

Patients with SBC were recruited from the Department of Neurosurgery at Huashan Hospital affiliated to Shanghai Medical School, Fudan University from 2007 to 2020 for this study. The diagnosis of SBC was made from clinical manifestations, laboratory tests, and imaging, and SBC was histologically confirmed in a blinded fashion by at least two senior pathologists after surgical resection. Subjects with previous malignant diseases or family history of SBC were excluded. The age and gender of human subjects providing patients are summarized in Supplementary Table S1. Fresh biopsies of chordomas were collected at the time of surgical resection and were processed for scRNA-seq. Paraffin-embedded tissue was used for IHC. Our study was pre-approved by The Institutional Review Board Huashan Hospital, Fudan University (NO. 2018-431) and informed consent were obtained for all human participants.

### Tissue dissociation and library preparation

Tumor samples were collected and transported in IMDM (12440053; Gibco): RPMI-1640 (A1049101; Gibco) 4:1 medium on ice. Single-cell suspensions were obtained from tumor biopsies through mechanical and enzymatic dissociation. Then the samples were washed with phosphate buffered saline (PBS; ThermoFisher Scientific). Viability was confirmed to be > 70% in all samples using trypan blue (15250061; ThermoFisher Scientific). Cell suspensions were filtered using a 70 μm filter (431751; Falcon), and dissociated cells were pelleted and resuspended in PBS with 1% bovine serum albumin (BSA; Sigma-Aldrich). Single cells were captured in droplet emulsions using the GemCode Single-Cell Instrument (10× Genomics), and scRNA-seq libraries were constructed per the 10× Genomics protocol using GemCode Single-Cell 3′ Gel Bead and Library V2 Kit. All libraries were sequenced on the NovaSeq 6000 Sequencing System (Illumina).

### scRNA-seq data processing

Raw fastq files were processed with Cell Ranger (version 4.0) using default mapping parameters coupled with the Homo sapiens GRCm38.95 genome reference. Raw gene expression matrics were generated, and empty droplets were identified using EmptyDrops^63^. Cells with a high proportion (> 20%) of transcript counts derived from mitochondria-encoded genes were removed. Possible doublets were also removed using R package DoubletFinder with assumed 5% doublets rates^64^. Cells with more than 500 UMI were considered for downstream analysis. Seurat was used to performing clustering analysis of single-cell data on a per-sample basis^65^. Genes expressed in more than 3 cells were selected and formed the processed digital gene expression matrix (DGE) with qualified cells. Then, DGE was ln (counts per million (CPM)/100 + 1) transformed, and the number of the gene was regressed out. About 2000 genes with an average expression of more than 0.01 and a dispersion greater than 0.45 were selected for initial principal component analysis (PCA). The number of principal components (PCs) for uniform manifold approximation and projection (UMAP) analysis was chosen according to the PCElbowPlot function and JackStrawPlot function. UMAP was used for visualization with min.dist = 0.75 for a more dense distribution. For clustering, we set different resolution parameters ranging from 0.5 and 2.5 in the “FindAllCluster” function and narrowed them down to certain cluster numbers by distinguishing differential genes among clusters. Resolution and the number of PCs were adjusted on a per-sample basis. The default Wilcoxon rank-sum test was used by running FindAllMarkers function in Seurat to find differentially expressed markers in each cluster. Finally, we annotate each cell type by extensive literature reading and searching for the specific gene expression pattern. All details regarding the Seurat analyses performed in this work can be found in the website tutorial (https://satijalab.org/seurat/v3.0/pbmc3k_tutorial.html).

### Multiple dataset integration and sub-clustering for major cell lineages

For integrated analysis across samples, we used Scanpy in a python environment for the whole datasets and per-lineage datasets^66^. Scanpy can be used for re-implementing the similar results of Seurat more efficiently. All details regarding the scanpy analyses performed in this work can be found in the website tutorial (https://scanpy-tutorials.readthedocs.io/en/latest/integrating-data-using-ingest.html). About 3000 highly variable genes according to their average expression and dispersion were selected for PCA analysis, and about 50 PCs were used for clustering and visualization for the whole dataset. For per lineage, about 2000 highly variable genes were selected, and adjusted PCs were used according to the elbow point. Scaling, PCA, and clustering were performed as described above. To regress out the batch effects from sample variation, BBKNN was performed by using ridge regression for the whole datasets and per-lineage datasets^67^. For malignant cells, we applied the anchor-based batch correction method to perform cluster analysis in order to further explore the common features of malignant cells among different patient. We integrated the data using reciprocal PCA (RPCA) in Seurat (https://satijalab.org/seurat/articles/integration_rpca.html) and employed sample identity to split cells.

### CNV estimation and classification of malignant cell

To infer CNVs from the scRNA-seq data, we used an approach described previously with the R code provided in https://github.com/broadinstitute/inferCNV with the default parameters. First, we considered these manual annotated immune cells, stromal cells, and epithelial cells as putative nonmalignant cells, and their CNV estimates were used to define a baseline^15^. We also regarded other cells expressing *TBXT, VIM*, and *S100A1* as putative malignant cells. Reference groups were adjusted based on the primary infer CNV results and then guided to the second round of analyses. The calculated CNV signal (*x*-axis) was defined as the mean square of the CNV estimates across all genomic locations. The calculated CNV R-scores (*y*-axis) were defined as the Pearson correlation coefficient between each cell’s CNV pattern and the average CNV pattern of the top 5% of cells from the same tumor for CNV signal. Finally, CNV *R*-scores of ≥0.1 was defined as malignant cells.

### Expression programs of intratumor heterogeneity

Malignant cells from each cancer sample were first ln(counts per million (CPM)/100 + 1) transformed and then center-scaled for each gene as described previously^55^. Only highly variable genes remained. After transformation of all negative values to zero, non-negative matrix factorization was performed using the run_NNMF.py function from a previously published NNMF analysis to analyze the data (https://github.com/YiqunW/NNMF/) using the following parameters: -rep 3 -scl “false” -miter 10000 -perm True -run_perm True -tol 1e-6 -a 2 -init “random”. Each NNMF analysis was repeated three times using different randomly initialized conditions, enabling us to evaluate reproducibility. Additionally, the analysis was initially performed using a broad range of *K* values. The results were then compiled using the integrate_and_output.sh script, and evaluated to identify the range of *K* values that gave the best results, which then guided the second round of analyses using a narrower set of *K* values. Finally, *K* = 20 was selected for all samples and resulted in a total of 120 programs across the 6 tumors. The 120 programs were compared by hierarchical clustering, using one minus the Pearson correlation coefficient overall gene scores as a distance metric. Eight clusters of programs were identified manually and used to define meta-signatures. The top 100 ranks of each sample were calculated. Genes were ranked by their average scores, and meta-signatures were selected for appearing in at least half samples. Then meta-signatures were used as input for GSEA enrichment for functional enrichment analysis^68^.

### Calculation of pathway activity score of each cell

To evaluate whether a pathway was enriched at the top ranking of expressed genes in every single cell, we calculated an activity score for each pathway by applying an area under the curve method named AUCell^69^. The pathway activity score was measured as the proportion of expressed genes in the query gene set and their relative expression rank compared to the other genes in each cell. The GO and KEGG pathway gene sets were downloaded from the website http://amigo.geneontology.org/amigo. The gene sets for the p-EMT module and radioresistant gene modules were calculated as mentioned above.

### Pathway enrichment analysis

We used clusterProfiler to perform gene ontology biological pathway enrichment analysis, and all the genes were taken as the universe. We considered biological pathways with *P*-value smaller than 0.05. Differentially expressed genes (DEG) for spatial distribution were calculated using FindAllMarkers function in the R package Seurat (Parameters: min.pct = 0.25, min.diff.pct = 0.25, logfc.threshold = 0.25).

### Identification and analysis of stem-like cells

We applied the anchor-based batch correction method from Seurat to perform integrated clustering analysis to explore further the common features of malignant cells among different patients. The function AddMetaScores in Seurat (v4.0.4) was used to calculate the stem score in each cell based on stem-cell markers as in previous research^30,33^. The score distribution in each sample was visualized using boxplot function in R. The pseudotime trajectory analysis in malignant cells was performed using scVelo^70^.

### Pseudotime trajectory analysis of EMT programs in cancer cells

EMT scores of cancer cells were calculated based on the KS method^41^ and visualized through boxplot in R. The KS method is based on a two-sample Kolmogorov–Smirnov test, which compares cumulative distribution functions (CDFs) of E and M signatures. The cells that showed significantly increased EMT scores were identified as the Mes-type, while the cells that showed significantly decreased EMT scores were identified as Epi-type. The cells identified as the Mes-type or the Epi-type were clustered and visualized using the method of uniform manifold approximation and projection (UMAP) with Seurat functions RunUMAP^71^.

In addition, to discover the cell-state transitions, the R package Monocle2 (v2.18.0) was used to analyze single-cell trajectories with the converted objects from the R package Seurat. Only genes with mean expression >0.1 were used for the analysis. The Monocle2: reduce Dimension function was utilized with the reduction method DDRTree and the default parameters. Results were visualized using the plot_cell_trajectory function and the plot_pseudotime_heatmap function in Monocle2^72^.

### Cell–cell interaction analysis

We used CellPhoneDB^73^ to conduct a systematic analysis of cell–cell communication based on a public repository of ligands, receptors, and their interactions. The enriched ligand–receptor interactions between two cell subsets were calculated based on a permutation test. We extracted significant ligand–receptor pairs with *P*-value < 0.05. The number of ligand–receptor pairs between cell types was visualized with the R package igraph (v1.2.6). The ligand–receptor pairs between cancer cells and stromal cells were plotted with R package ggplot2 (v3.3.5).

### Comparison analysis with the HCL

We performed pseudo-cell processing on the HCL data to achieve a similar sequence depth with chordoma datasets^24^. Each pseudo-cell was an average of 75 cells randomly selected from the same cell cluster. Then, we performed standardized CPM processing and log1p processing. We used the FindAllMarkers function in the R package Seurat (Parameters: min.pct = 0.25, min.diff.pct = 0.25, logfc.threshold = 0.25). We extracted the top 20 marker genes specifically expressed in chordoma cancer cells with low expression levels in HCL. Mitochondria-encoded genes and ATP genes were excluded, which were more related to cell activities in sample processing. Then, we used the Python package pandas to calculate Pearson’s correlation coefficients between genes. After the absolute value processing, we constructed a correlation matrix. We used the “Circular Layout” in Cytoscape to visualize the gene-gene correlation network in the top 20 DEGs and the genes with the highest correlation coefficients in chordoma cancer cells^74^. Gene interaction network was conducted using the Pathway Commons (http://www.pathwaycommons.org).

### Cell culture and reagents

The UM‑Chor1 cell line was purchased from the American Type Culture Collection (CRL-3270; ATCC, Manassas, VA, USA). UM‑Chor1 cells were cultured in IMDM: (12440053; Gibco): RPMI-1640 (A1049101; Gibco) 4:1 medium supplemented with 10% FBS (10099141C; Gibco) and 1% antibiotic mixture (15240062; Gibco). The cells were grown in a humidified 5% CO_2_ atmosphere at 37 °C.

### Radiation of cell lines

Seeded onto 6-well plates were 3 × 10^5^ UM‑Chor1 cells. Once cells reached 90% confluence, cells were irradiated by using a special platform (Small animal radiation research platform, SARRP, Gulmay Medical Co., Ltd), with the following parameters: 3.845 Gy/min at 220 kV, 13 mA; the source-to-skin distance (SSD) for a fixed fluoroscopy unit was 35 cm, and the irradiation area was 15 × 15 cm. Cells were exposed to irradiation or normal control. Then cells were observed for 48 h and the culture medium was changed with the cells washed three times in serum-free medium.

### RNA-seq of cell lines

For RNA-seq analyses, X-Ray treated cells, and untreated cells were collected in Trizol (Thermo Fisher Scientific), and total RNAs were extracted. The cDNA was synthesized using a High-Capacity cDNA Reverse Transcription Kit (Applied Biosystems, Foster City, CA, USA) according to the manufacturer’s instructions. RNA-seq libraries were generated with a NEB Next Directional RNA Library Prep Kit for Illumina (New England Biolabs, Ipswich, MA, USA). Resulting libraries were size-selected by agarose gel electrophoresis and subsequently sequenced using an Illumina HiSeq-X platform with a 2 × 150 bp modality. Paired-end RNA-seq reads with 150 bp in each end were aligned to the Mus musculus genome (GRCm38) using Subread Aligner57 with its default parameter settings, and reads were counted using Feature Counts v1.5.3. DESeq2 was used to identify differentially expressed genes with false discovery rate (FDR) < 0.05 and fold-change ≥ 2. KEGG enrichment pathway analyses were performed using KOBAS.

### siRNA transfection

The siRNA sequences of target gene markers were synthesized by GenePharma. Cells were seed in a 6-well plate with a density of 5 × 10^5^ cells/well. After 24 h and 70%-80% confluence, the cells were added with siRNA (50 nM) in serum-free medium using RNAiMAX (13778030; Invitrogen) per manufacturer’s instructions. After incubation for 20 min at RT, the medium in each well was then changed with complete medium with 10% heat-inactivated fetal bovine serum for another 48 h. The siRNA sequences are as follows:

TGFBI siRNA1 forward: 5′-GCACUAAUAGGAAGUACUUTT-3′

TGFBI siRNA1 reverse: 5′-AAGUACUUCCUAUUAGUGCTT-3′

TGFBI siRNA2 forward: 5′-CCAAUUGAUGCCCAUACAATT-3′

TGFBI siRNA2 reverse: 5′-UUGUAUGGGCAUCAAUUGGTT-3′

TGFBI siRNA3 forward: 5′-GCGGCUAAAGUCUCUCCAATT-3′

TGFBI siRNA3 reverse: 5′-UUGGAGAGACUUUAGCCGCTT-3′

CTSL siRNA1 forward: 5′-CCAUUGUGGAAUUGCCUCATT-3′

CTSL siRNA1 reverse: 5′-UGAGGCAAUUCCACAAUGGTT-3′

CTSL siRNA2 forward: 5′-GACUGUAGCAGUGAAGACATT-3′

CTSL siRNA2 reverse: 5′-UGUCUUCACUGCUACAGUCTT-3′

CTSL siRNA3 forward: 5′-GUCGGAUACACACUCGAAUTT-3′

CTSL siRNA3 reverse: 5′-AUUCGAGUGUGUAUCCGACTT-3′

### Lentiviral transfection

Three oligonucleotides, which contain TGFBI shRNA sequence (5′-GCATGACCCTCACCTCTATGT-3′, 5′-GGGACATGCTCACTATCAACG-3′ and 5′-GCTTCGGAACCACATAATTAA-3′, respectively, were synthesized by Genomeditech. Then, the shRNA oligos were inserted into the recombinant vector (PGMLV-Hu6-MCS-CMV-mScarlet-PGK-Puro) to form TGFBI shRNA plasmids.

For TGFBI overexpression, the PGMLV-6395-TGFBI plasmid was also produced by Genomeditech.

Next, the plasmid, packaging vectors psPAX2 and pMD2.G (ratio 3:2:1) were transfected into the HEK293T cells using PEI (BMS1003-A; Invitrogen) to produce lentiviral particles.

The media was changed after 12 h. The media containing the virus were collected 48 h after the medium was changed. Viral supernatants were centrifuged at 1500 × *g* for 45 min and viral pellets were resuspended with RPMI-1640 medium.

Cells were seeded in a 6-well plate with a density of 5 × 10^5^ cells/well. After 24 h and 70%–80% confluence, the cells were added with lentivirus containing TGFBI shRNAs (50 nM) or scr-shRNA (50 nM, as control) respectively. After incubation for 18 h at 37 °C, the cells were passaged and seeded into new 6-well plates. 36 h after the cell passage, the cells were selected with puromycin (1 μg/mL, A1113803; Gibco).

### Drug treatment

The chemicals used in the study are LY364947 (HY-13462; MedChemExpress), Vactosertib (HY-19928; MedChemExpress), PF06952229 (HY-136244; MedChemExpress); Z-FY-CHO (HY-128140; MedChemExpress) and YL-13027 (Donated by Shanghai Yingli Pharmaceutical Co., Ltd.). Details of chemicals used can be found in the corresponding figure legends.

### Matrigel invasion assay

Transwell invasion assays were performed as follows. Firstly, 100 μL overnight melted matrigel (diluted with the serum-free medium at 1:20, cat#354277; Corning) was added to the upper transwell chamber (353097; falcon) and incubated at 37 °C for 1 h. Secondly, the liquid in the chamber was removed, and the upper and lower chamber were added with 100 μL or 600 μL serum-free IMDM:1640 4:1 medium, respectively. Afterward, the chamber was kept static at 37 °C for 30 min and sipping up the medium suction medium. Thirdly, 100 μL suspensions of cells were prepared and added to the upper chamber (1 × 10^5^ cells/well). The lower chambers were added with 600 μL culture medium containing 10% FBS. Subsequently, the chamber was incubated at 37 °C for 24 h and the cells remaining in the upper chamber were wiped with a cotton swab. The invaded cells were fixed with formaldehyde and stained with 0.1% crystal violet dye. Finally, cells in 5 microscopic fields (at ×200 magnification) were counted and photographed as previously used^75^. All the invasion and migration assays were performed in triplicate and repeated 3 independent times.

### Staining of tissue sections

Tumor samples were fixed in 4% paraformaldehyde for 24 h and then embedded in paraffin. Paraffin blocks were cut into 5 μm sections and used for hematoxylin and eosin (HE), immunohistochemistry (IHC), or Immunofluorescence (IF) staining.

For IHC, slides were deparaffinized through xylenes and graded ethanol, then performed antigen retrieval using citrate buffer at pH 6.0. After washing, slides were blocked with 0.3% H_2_O_2_ and 5% normal goat serum sequentially, followed by staining with primary antibody at 4 °C overnight. Next, slides were incubated with EnVision FLEX/HRP (SM802, DAKO, Glostrup, Denmark) at RT for 20 min, followed by using EnVison FLEX DAB + CHROMOGEM and EnVision FLEX substrate buffer (DM827 and SM803, DAKO, Glostrup, Denmark) to visualize staining signals under light microscopy, finally counterstained using hematoxylin solution. Finally, stained slides were scanned using Ocus (Grundium, Tampere, Finland) and analyzed with Qupath software.

For IF, procedures before primary antibodies incubation were the same as IHC, except for H_2_O_2_ blocking. First, slides were incubated with primary antibody at 4 °C overnight, followed by secondary antibodies. Finally, the slides were counterstained with DAPI in an antifade solution (DAPI Fluoromount-G, 0100-20, SouthernBiotech) and then mounted. Images were taken with Leica SP8 confocal microscope.

### Western blotting

Proteins were extracted by SDS cell lysis buffer (P0013G; Beyotime) supplemented with protease inhibitor cocktail (87785; Thermo Scientific). Protein quantification was measured by the Pierce BCA protein assay kit (23225; Thermo Fisher Scientific). The protein bands were detected by conventional protocols for western blotting. Proteins were detected by using specific primary antibodies against secondary antibodies (Cell Signaling Technology). The following antibodies were used: monoclonal anti-GAPDH (ab181602,1:4000; abcam), monoclonal anti-TGFBI (ab170874, 1:1000; abcam), polyclonal anti-ZEB2 (abs131374, 1:1000; absin), monoclonal anti-VIM (cat#5741, 1:1000; Cell Signaling Technology), polyclonal anti-CTSL (ab200738, 1:1000; abcam), monoclonal anti-alpha tubulin (PTM-5442, 1:2000; ptmbiolabs), anti-rabbit IgG (7074, 1:2000; Cell Signaling Technology), and anti-mouse IgG (7076, 1:2000; Cell Signaling Technology).

### RT-qPCR

Total RNA was extracted from tissue samples and cells using TRIzol reagent (15596018; Invitrogen) after washing with PBS. cDNA was synthesized from purified RNA using a SuperScript III First-Strand cDNA synthesis system (18080051; Invitrogen) according to the manufacturer’s instructions. SYBR Green PCR Master Mix (Q331-02; Vazyme) was used for PCR amplification and a real-time PCR machine (iQ5, Bio-Rad Laboratories) was used to quantify the expression of mRNAs. GAPDH was used as endogenous control and the expression levels were quantified using 2^−∆∆Ct^ method. The qPCR primers sequences are as follows:

TGFBI forward: 5′-CACTCTCAAACCTTTACGAGACC -3′

TGFBI reverse: 5′-CGTTGCTAGGGGCGAAGATG -3′

ZEB2 forward: 5′-CAAGAGGCGCAAACAAGCC -3′

ZEB2 reverse: 5′-GGTTGGCAATACCGTCATCC -3′

VIM forward: 5′-GACGCCATCAACACCGAGTT -3′

VIM reverse: 5′-CTTTGTCGTTGGTTAGCTGGT -3′

GAPDH forward: 5′-GGAGCGAGATCCCTCCAAAAT -3′

GAPDH reverse: 5′-GGCTGTTGTCATACTTCTCATGG -3′

ACTB forward: 5′-CATGTACGTTGCTATCCAGGC -3′

ACTB reverse: 5′-CTCCTTAATGTCACGCACGAT -3′

CTSL forward: 5′-AAACTGGGAGGCTTATCTCACT -3′

CTSL reverse: 5′-GCATAATCCATTAGGCCACCAT -3′

### Subcutaneous xenografts and drug treatment

UM-Chor1 cells (1 × 10^7^) were suspended in 100 mL PBS and injected subcutaneously into the back of BALB/c nude mice (Beijing Vital River Laboratory Animal Technology Co. (Shanghai)). For experiments with TGF-β inhibitors, mice carrying 50 mm^3^ subcutaneous tumors on average were randomized to receive daily treatment with 30 mg/kg of YL-13027 or an equal volume of normal saline by oral gavage. The tumor diameters were measured with a caliper, and the tumor volumes were estimated using the formula: 0.5 × length × width^2^. The mice were sacrificed when the tumors in the control group had reached the maximal size allowed by the Institutional Animal Care and Use Committee. All procedures performed in this study were in accordance with instructions and permissions of department of laboratory animal science, Fudan University. All mice were drug and test naive prior to initiating the experimental procedures performed and described in this paper.

### QuPath image analysis

Hematoxylin & Eosin and immunohistochemistry images were acquired by Ocus whole-slide scanner (Grundium, Tampere, Finland) and processed with Qupath software 0.2.2. First, images are preprocessed by the build-in stain vector estimator. Second, Cells with shape and stain parameters in each area were identified by build-in cell detection via nucleus stain (hematoxylin). The thresholds were set to 0.2, 0.4, and 0.6 for the mean DAB optical density (OD) to calculate the *H*-score. The positive percentage was normalized by the cell number of tumor areas in each slide. For nucleus-positive staining, the mean DAB OD of the nucleus was substrated by the mean DAB OD of cytoplasm to reduce false-positive cells. Finally, scripts of the whole-slide images analysis protocol above were created, batch performed on each set of images, and further checked by two expert pathologists.

### Clinical trial of YL-13027 treatment

#### Study design and participants

We performed a single-arm, open-label, single and multiple-dose-escalation phase I clinical study. The clinical trial was registered in National Clinical Trial (NCT03869632/https://clinicaltrials.gov/ct2/show/NCT03869632) and approved by Medical Ethics Committee of Huashan Hospital, Fudan University, Shanghai, China. The inclusion and exclusion criteria were summarized in Supplementary Table S12.

#### Procedures

YL-13027 was provided by the sponsor (currently under development by Shanghai YingLi Pharmaceutical Co. Ltd) in the form of oral enteric capsule in 10 mg and 50 mg. Patients were followed up every four weeks, MRI evaluations were performed every eight weeks. All adverse events were monitored throughout the study period (until 28 days after participants’ last YL-13027 dose) and graded according to the National Cancer Institute’s Common Terminology Criteria for Adverse Events (CTCAE; version 5.0). Dose-limiting toxicity was defined as a grade 3 adverse event attributable to study treatment within 28 days after YL-13027 treatment. Resumption of treatment for patients with dose-limiting toxicity was permitted (when clinically appropriate) if the severity of the toxicity fell to grade I or lower and treatment was interrupted for no more than 2 weeks. Complete clinical histories were taken at screening. MRI evaluations were done at baseline and every eight weeks after YL-13027 therapy. MRIs were independently reviewed by an expert radiologist based on RECIST 1.1 criteria. Partial response (PR) requires at least a 30% decrease in the sum of diameters of target lesions taking as reference the baseline sum diameters. Stable disease (SD) is defined by neither sufficient shrinkage to qualify for PR nor sufficient increase to qualify for PD, taking as reference the smallest sum diameters while on the study. Progression disease (PD) requires any of the following: at least a 20% increase in the sum of diameters of target lesions, taking as reference the smallest sum in the study (this includes the baseline sum if that is the smallest in study). In addition to the relative increase of 20%, the sum must also demonstrate an absolute increase of at least 5 mm (Note: the appearance of one or more new lesions is also considered progression). If treatment discontinuation was anticipated before the expected time, patients were encouraged to undergo MRI earlier. Laboratory tests for hematology, chemistry, and urinalysis were done 14 days before the first dose of YL-13027. Physical examinations and assessments of PFS performance status were done every eight weeks. If progressive disease (PD) was observed after assessment by RECIST 1.1 criteria, patients dropped out from the trial.

#### Outcomes

Primary objective was to determine the safety, tolerability, and maximum tolerated dose (MTD) of YL-13027 tablets in patients with advanced solid tumors by single and multiple oral administrations. Secondary objectives included: 1. to observe the pharmacokinetic profiles of YL-13027 in patients with advanced solid tumors; 2. to preliminarily evaluate the efficacy of YL-13027 treatment in patients with advanced solid tumors; 3. to explore and observe changes in biomarker indicators TGF-β1, pSMAD2/pSMAD3, total SMAD2, and SMAD3.

## Supplementary information

Supplementary Fig S1

Supplemental Fig S2

Supplemental Fig S3

Supplemental Fig S4

Supplemental Fig S5

Supplemental Fig S6

Supplemental Fig S7

Supplemental Fig S8

Supplemental Fig S9

Supplemental Fig S10

Supplemental Tab S1

Supplemental Tab S2

Supplemental Tab S3

Supplemental Tab S4

Supplemental Tab S5

Supplemental Tab S6

Supplemental Tab S7

Supplemental Tab S8

Supplemental Tab S9

Supplemental Tab S10

Supplemental Tab S11

Supplemental Tab S12

Supplemental Tab S13

Supplemental Tab S14

## Data Availability

All the scRNA-seq raw sequencing data have been deposited into the National Genomics Data Center (NGDC, http://bigd.big.ac.cn/) under the accession number PRJCA009785. Any additional information required to reanalyze the data reported in this paper is available from the lead Correspondence upon request.
