# Supplementary material for: Single-cell transcriptome reveals cellular hierarchies and guides p-EMT-targeted trial in skull base chordoma
Source: Cell Discov. 2022 Sep 20;8:94. doi: 10.1038/s41421-022-00459-2 (PMC9489773; doi:10.1038/s41421-022-00459-2)
Supplement: Supplementary file 1 — Supplementary Fig S1 [file 41421_2022_459_MOESM1_ESM.pdf]

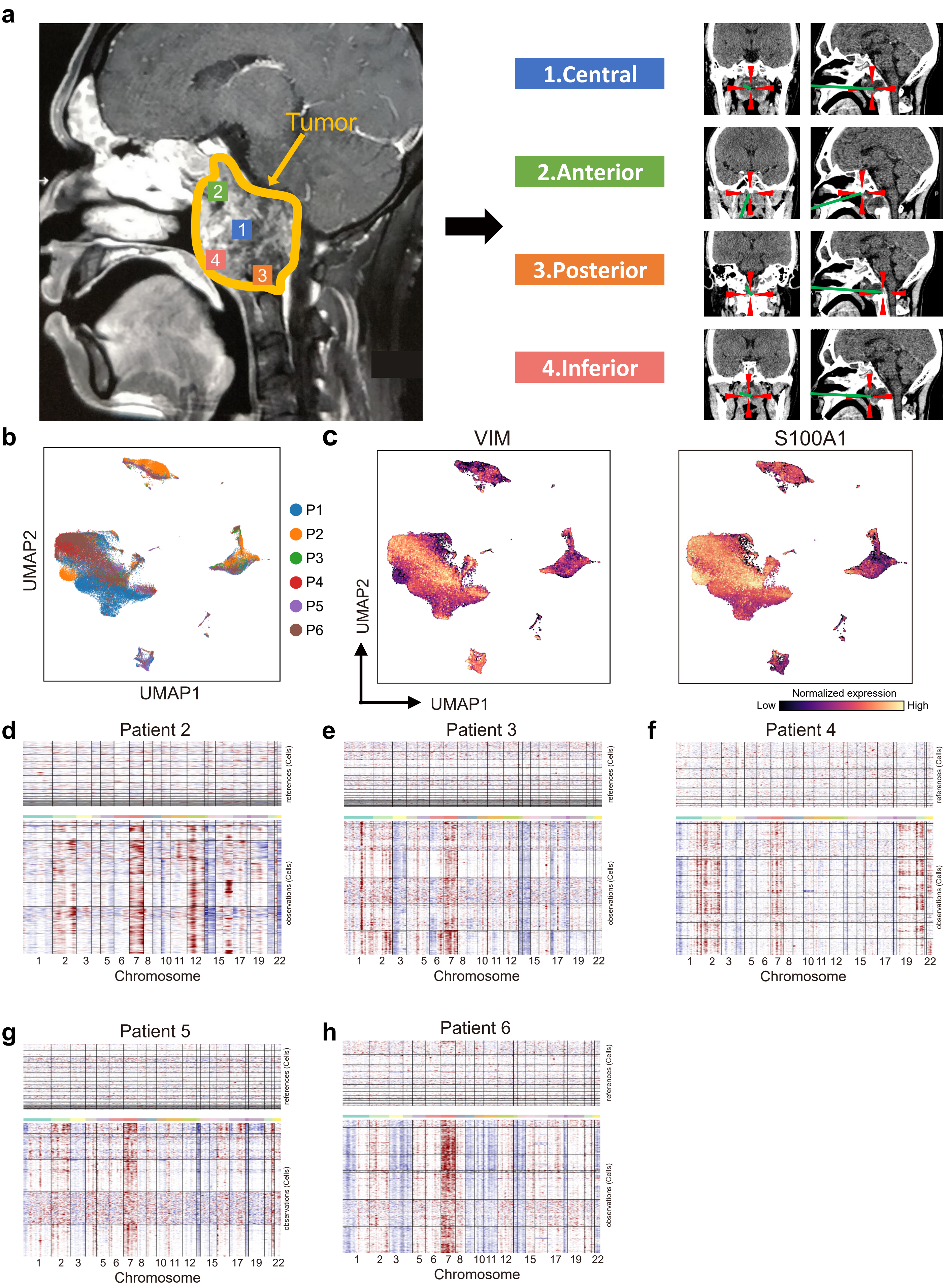

Supplementary Fig. 1 SBC Sample Collection and Expression Heterogeneity in the SBC. a) Four samples in anterior, posterior, inferior and central region of SBC from two patients under the intra-operative neuro-navigation. b) Umap plot of all cells from six patients. Each of the clusters contained cells from different patients, indicating that cell types and expression are largely consistent across SBC and do not represent patient-specific subpopulations or batch effects. c) Umap plot of two known malignant cell markers of SBC. d-h) Heatmaps of CNVs in Patients 2-6.
