# Supplementary material for: Single-cell transcriptome reveals cellular hierarchies and guides p-EMT-targeted trial in skull base chordoma
Source: Cell Discov. 2022 Sep 20;8:94. doi: 10.1038/s41421-022-00459-2 (PMC9489773; doi:10.1038/s41421-022-00459-2)
Supplement: Supplementary file 2 — Supplemental Fig S2 [file 41421_2022_459_MOESM2_ESM.pdf]

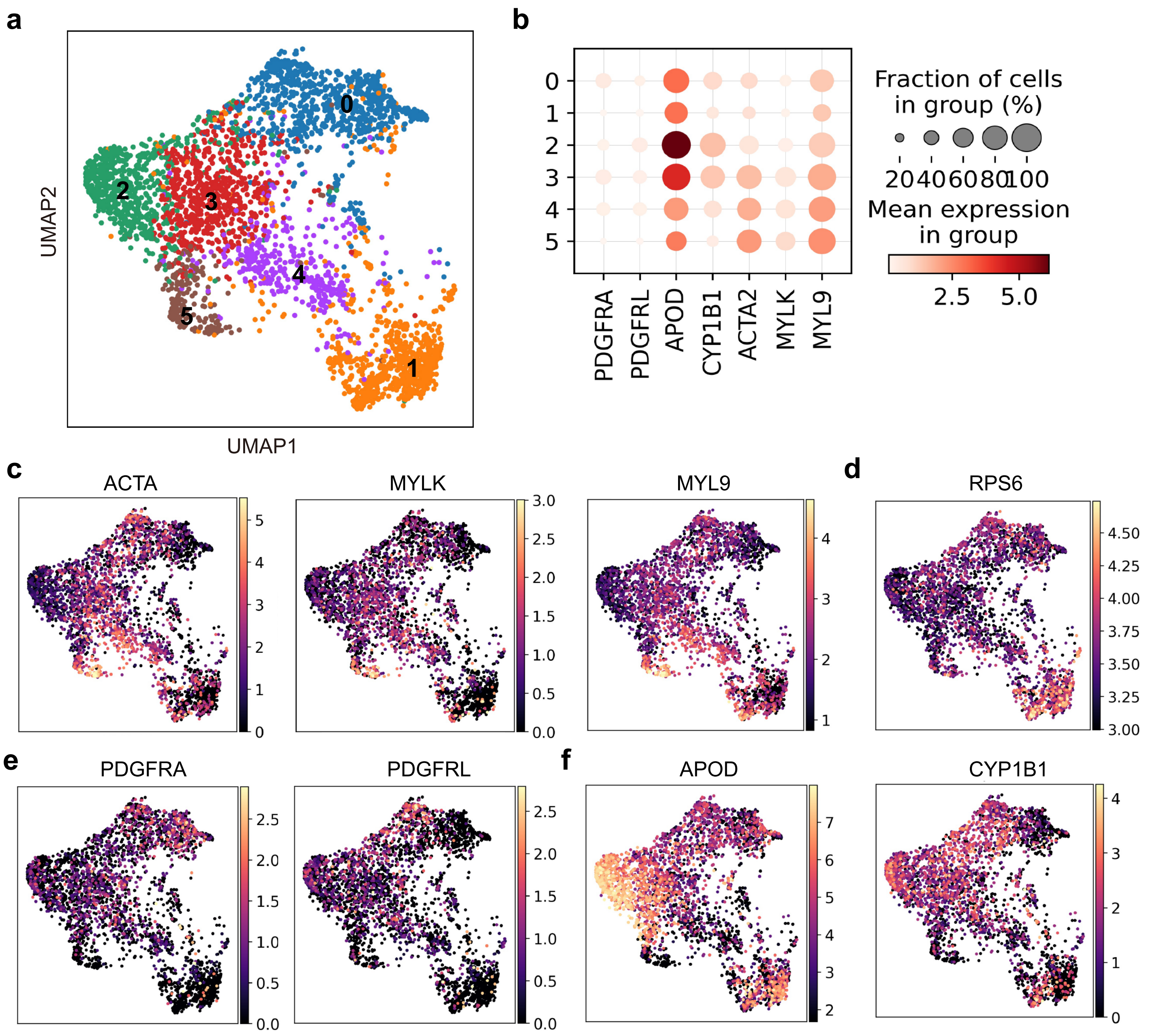

Supplementary Fig. 2 Expression Heterogeneity of Stromal Cells in the SBC Ecosystem. a) ~3,700 stromal cells were clustered into six clusters according to known marker genes. b) Dot plot showing the expression of known marker genes in these six stromal cell clusters. c-f) Umap plots of known stromal markers.
