# Supplementary figures and images for: Single-cell transcriptome reveals cellular hierarchies and guides p-EMT-targeted trial in skull base chordoma
Source: Cell Discov. 2022 Sep 20;8:94. doi: 10.1038/s41421-022-00459-2 (PMC9489773; doi:10.1038/s41421-022-00459-2)

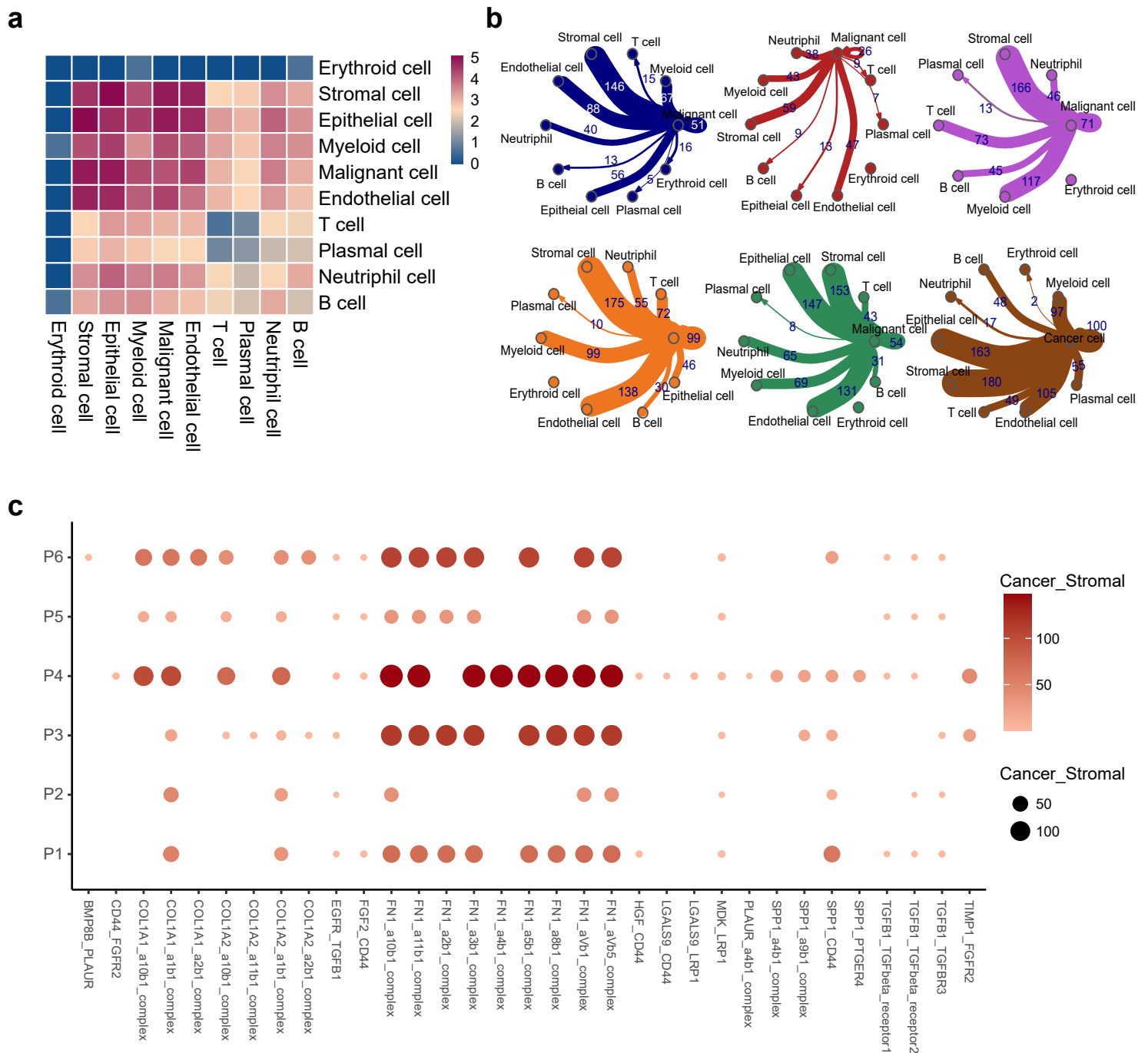

Supplement: Supplementary file 3 — Supplemental Fig S3 [file 41421_2022_459_MOESM3_ESM.pdf]
