# Supplementary material for: Single-cell transcriptome reveals cellular hierarchies and guides p-EMT-targeted trial in skull base chordoma
Source: Cell Discov. 2022 Sep 20;8:94. doi: 10.1038/s41421-022-00459-2 (PMC9489773; doi:10.1038/s41421-022-00459-2)
Supplement: Supplementary file 4 — Supplemental Fig S4 [file 41421_2022_459_MOESM4_ESM.pdf]

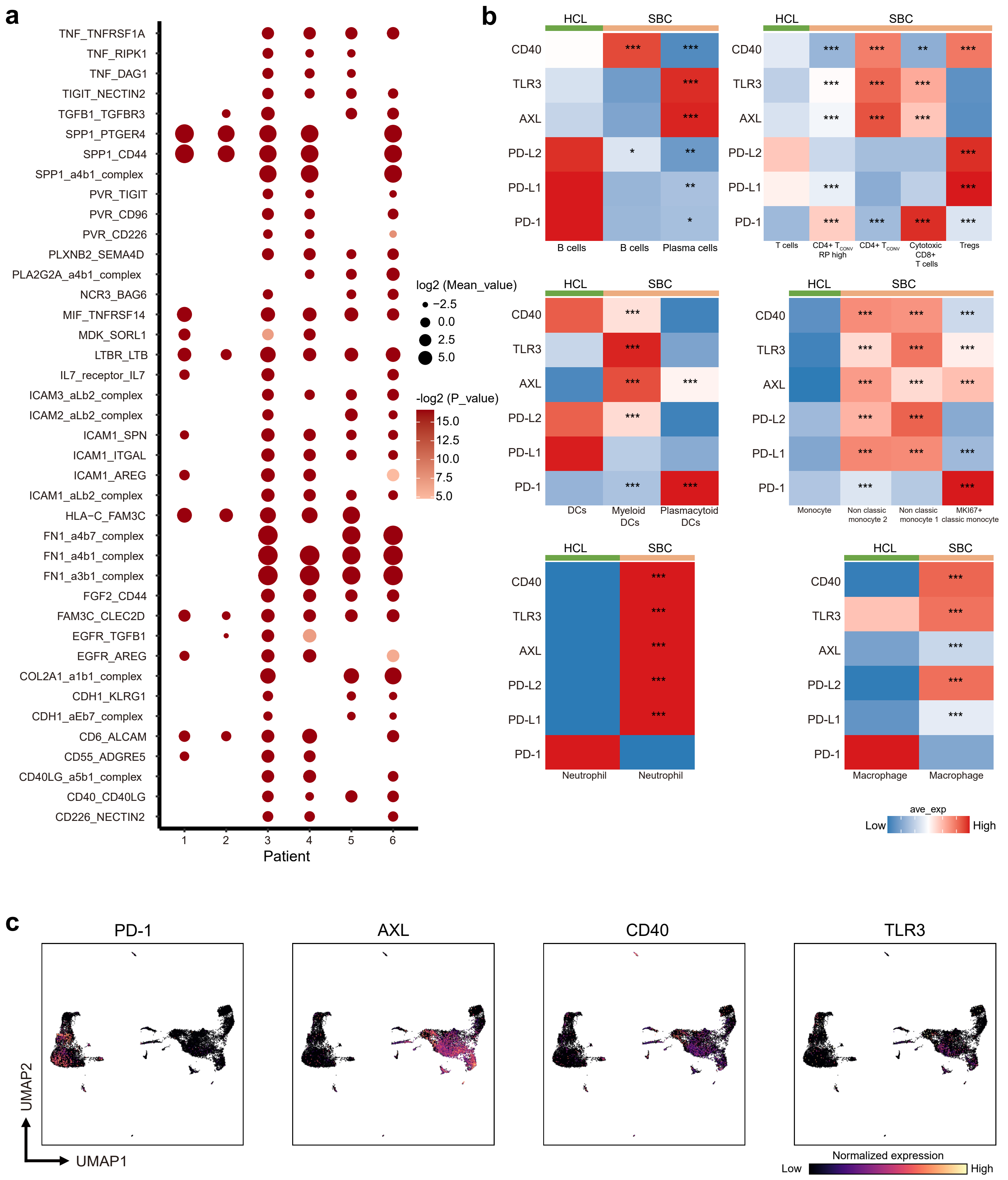

Supplementary Fig. 4 CellPhoneDB analysis of malignant and immune cells in SBC. a) The ligand–receptor pairs from cellphoneDB between cancer cells and T cells were plot with R package ggplot2. b) The heatmaps of the comparison between HCL and SBC-infiltrated immune cells. Each type of SBC-infiltrated immune cells was compared with the corresponding immune subtype in HCL separately. ***: p-value ≤ 0.001; **: p-value ≤ 0.01, *: p-value ≤ 0.05. c) Umap plot of PD-1/PD-L1 and related regulators in Immune cells.
