# Supplementary material for: Single-cell transcriptome reveals cellular hierarchies and guides p-EMT-targeted trial in skull base chordoma
Source: Cell Discov. 2022 Sep 20;8:94. doi: 10.1038/s41421-022-00459-2 (PMC9489773; doi:10.1038/s41421-022-00459-2)
Supplement: Supplementary file 5 — Supplemental Fig S5 [file 41421_2022_459_MOESM5_ESM.pdf]

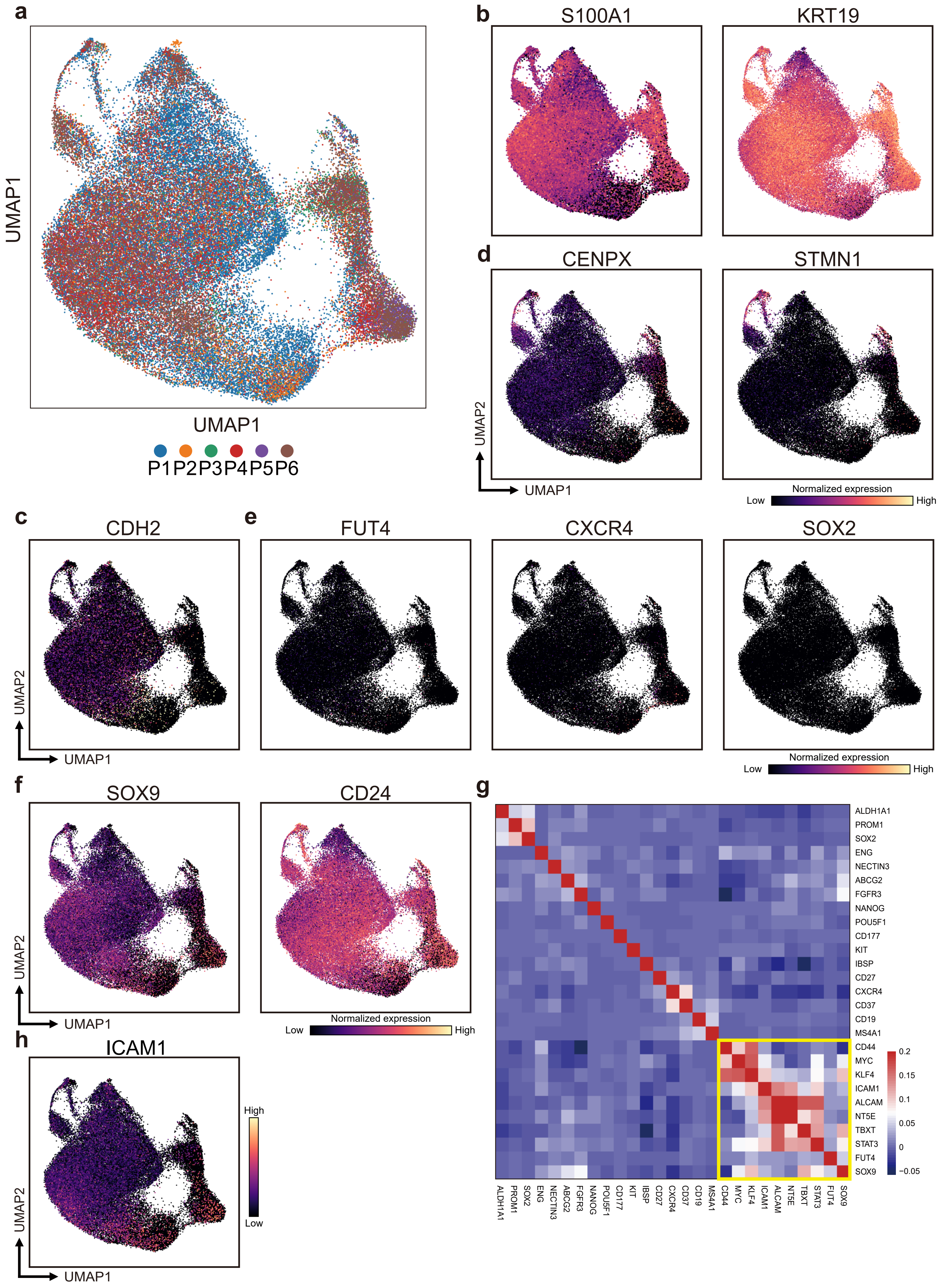

a
UMAP1
UMAP1
P1 P2 P3 P4 P5 P6
b
S100A1
KRT19
d
CENPX
STMN1
UMAP2
UMAP1
Normalized expression
Low
High
c
CDH2
e
FUT4
CXCR4
SOX2
UMAP2
UMAP1
Normalized expression
Low
High
f
SOX9
CD24
UMAP2
UMAP1
Normalized expression
Low
High
g
ALDH1A1
PROM1
SOX2
ENG
NECTIN3
ABCG2
FGFR3
NANOG
POU5F1
CD177
KIT
IBSP
CD27
CXCR4
CD37
CD19
MS4A1
CD44
MYC
KLF4
ICAM1
ALCAM
NT5E
TBXT
STAT3
FUT4
SOX9
0.2
0.15
0.1
0.05
0
−0.05
h
ICAM1
High
Low
UMAP2
UMAP1

Supplementary Fig. 5 Expression Heterogeneity and Radio-resistant Mechanism of Malignant Cells in the SBC. a) Umap plot of malignant cells from six patients. Each of the clusters contained cells from different patients, indicating that cell types and expression are largely consistent across SBC and do not represent patient-specific subpopulations or batch effects. b-f) Umap plot of subpopulation markers. g) Correlation analysis of stem cell markers in SBC. Ten genes (in the yellow box) were used as a set to calculate the stem score of each malignant cell in SBC. h) Umap of *ICAM1*, a stem cell associated marker which is highly expressed in cluster 3.
