# Supplementary material for: Single-cell transcriptome reveals cellular hierarchies and guides p-EMT-targeted trial in skull base chordoma
Source: Cell Discov. 2022 Sep 20;8:94. doi: 10.1038/s41421-022-00459-2 (PMC9489773; doi:10.1038/s41421-022-00459-2)
Supplement: Supplementary file 6 — Supplemental Fig S6 [file 41421_2022_459_MOESM6_ESM.pdf]

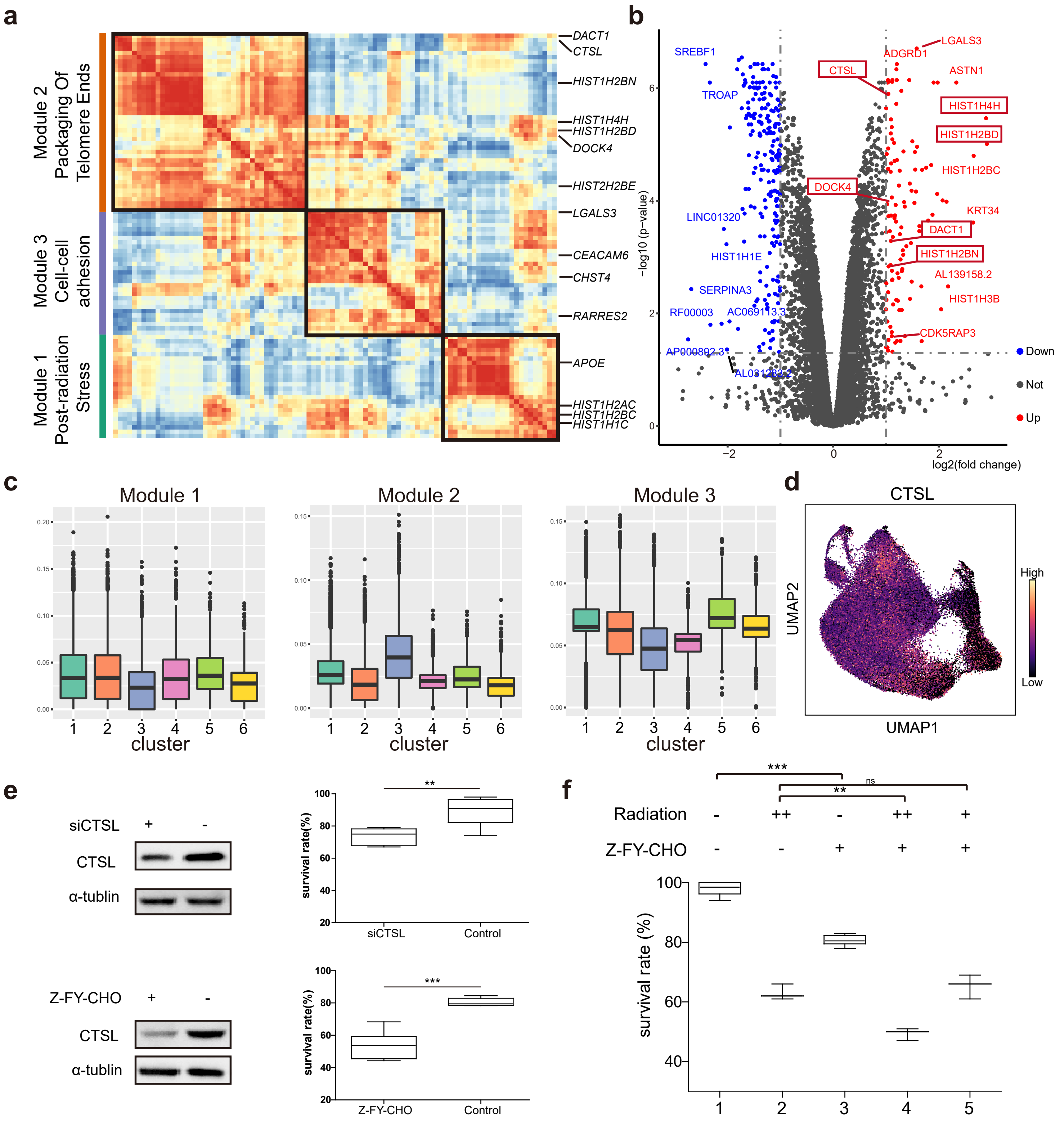

Supplementary Fig. 6 The Radio-resistant Mechanism of Malignant Cells in the SBC. a) Heatmap depicts three expression modules that were elevated after the radiation of UM-chor1. b) The Volcano Plot of RNA change of UM -chor1 after 64 Gy radiation and two-day culture. Markers involved in packaging of telomere ends were labeled in the red boxes. c) The distribution of score of each cell in six clusters according to the expression of three modules. d) The UMAP plot of *CTSL*, which was a known radio-resistance related gene and found included in module 2. e) The siCTSL (up) and Z-FY-CHO (CTSL inhibitor, down) decreased the survival rate of UM-chor1 after receiving 64 Gy radiation. Z-FY-CHO +: 10 μM, ***: p-value ≤ 0.001, **: p-value ≤ 0.01. f) Z-FY-CHO increased the radiosensitivity of UM-Chor1 at the concentration of 10 μM. Radiation +: 32 Gy, Radiation ++: 64Gy, Z-FY-CHO +: 10μM, ***: p-value ≤ 0.001, **: p-value ≤ 0.01, ns: no significance.
