# Supplementary material for: Single-cell transcriptome reveals cellular hierarchies and guides p-EMT-targeted trial in skull base chordoma
Source: Cell Discov. 2022 Sep 20;8:94. doi: 10.1038/s41421-022-00459-2 (PMC9489773; doi:10.1038/s41421-022-00459-2)
Supplement: Supplementary file 7 — Supplemental Fig S7 [file 41421_2022_459_MOESM7_ESM.pdf]

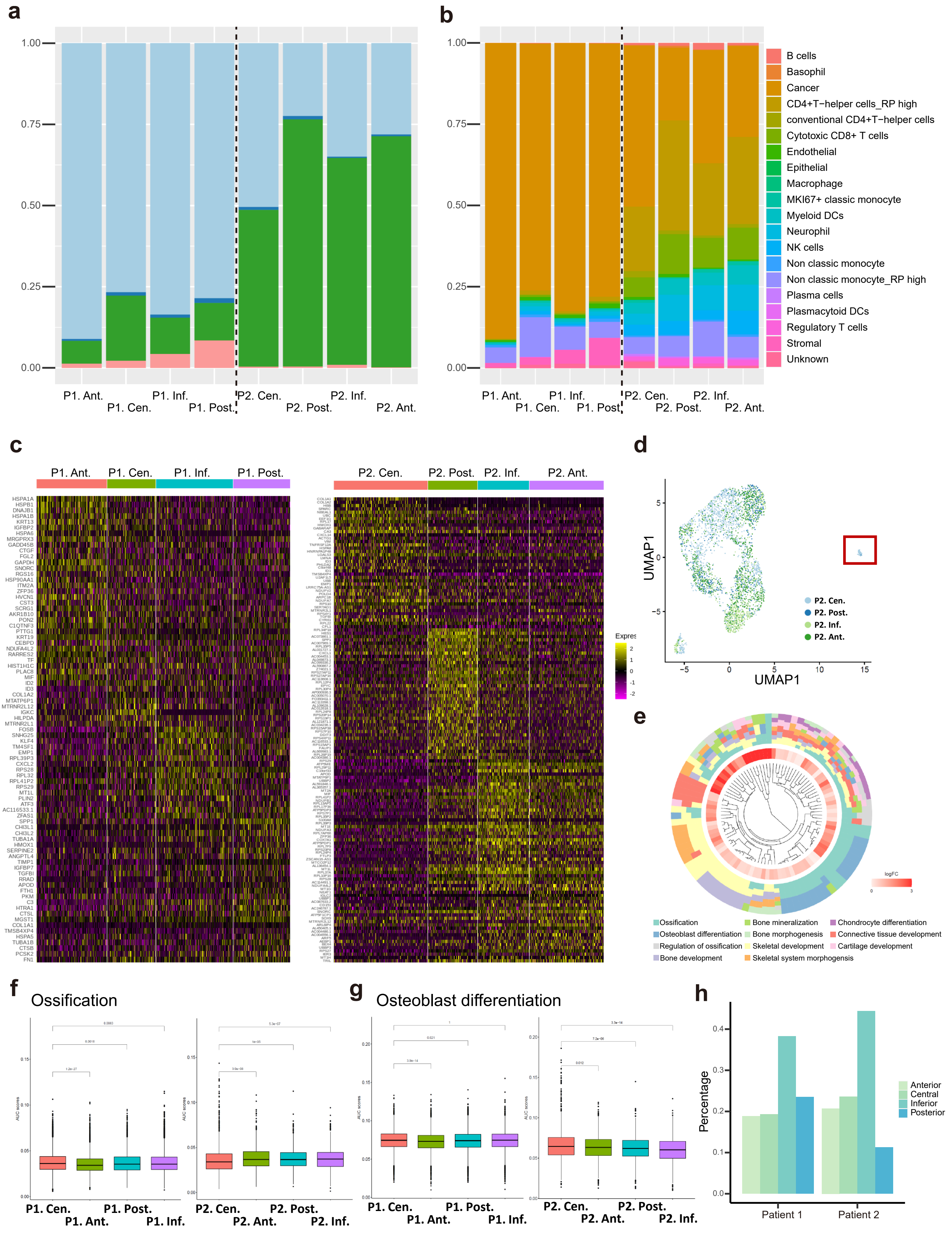

Supplementary Fig. 7 Spatial Distribution of subpopulation in SBC. a-b) The cellular composition of all cells (a) and immune cells (b) in different region of SBC from two patients. c) Heatmap of expression of four regions of two SBC patients. d) All cancer cells from Patient 2 were clustered and visualized. A cluster were almost only including cells from the central location samples (red box). e) Top GO pathways enriched by the up-regulate genes in red box cluster in (d) including ossification and osteoblast differentiation. f-g) The score of ossification (f) and osteoblast differentiation (g) in central region is significantly elevated in both 2 patients. h) The spatial distributions of stem-like cells in Patients 1 and 2.
