# Supplementary material for: Single-cell transcriptome reveals cellular hierarchies and guides p-EMT-targeted trial in skull base chordoma
Source: Cell Discov. 2022 Sep 20;8:94. doi: 10.1038/s41421-022-00459-2 (PMC9489773; doi:10.1038/s41421-022-00459-2)
Supplement: Supplementary file 8 — Supplemental Fig S8 [file 41421_2022_459_MOESM8_ESM.pdf]

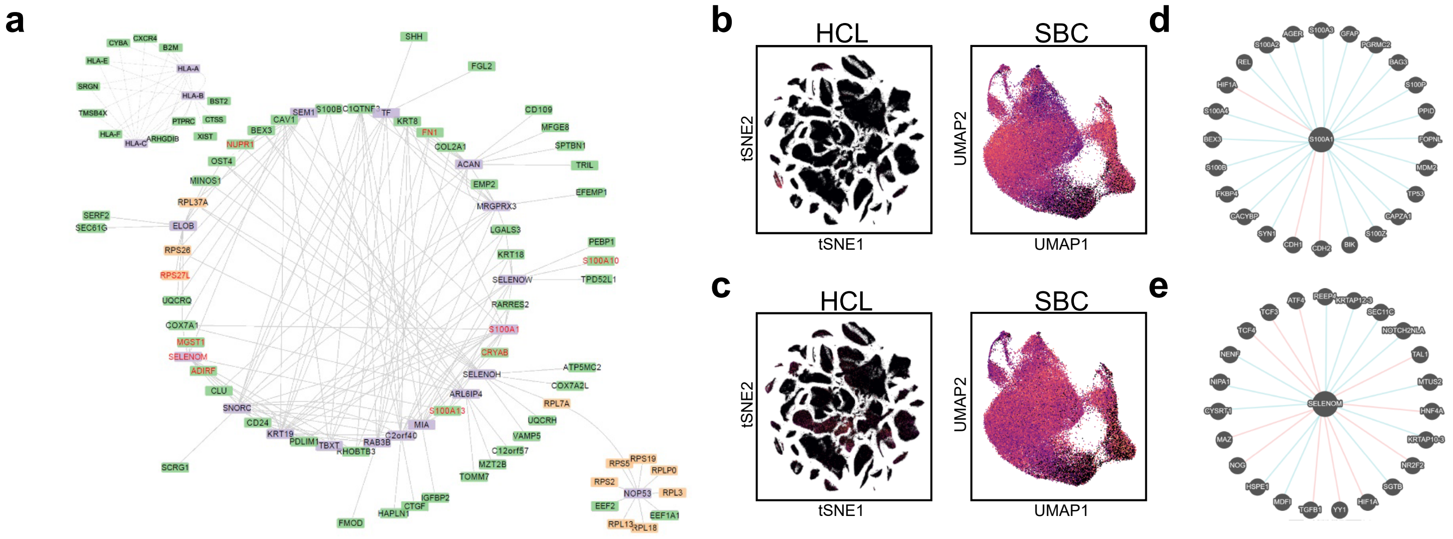

Supplementary Fig. 8 SBC target searching based on the HCL also highlight p-EMT program. a) Correlation networks of the top 20 highly expressed genes and their most relevant genes in SBC. The top expressed genes are the core genes in purple. The most relevant genes are in green and yellow (RPs). The genes involved in p EMT program were in red font. b-c) Feature plots of S100A1(b) and SELENOM (c) in HCL and SBC map. d-e) The interacting gene analysis using pathway commons. The blue and red lines represent the binding, expression controlled genes, respectively.
