# Supplementary material for: Single-cell transcriptome reveals cellular hierarchies and guides p-EMT-targeted trial in skull base chordoma
Source: Cell Discov. 2022 Sep 20;8:94. doi: 10.1038/s41421-022-00459-2 (PMC9489773; doi:10.1038/s41421-022-00459-2)
Supplement: Supplementary file 9 — Supplemental Fig S9 [file 41421_2022_459_MOESM9_ESM.pdf]

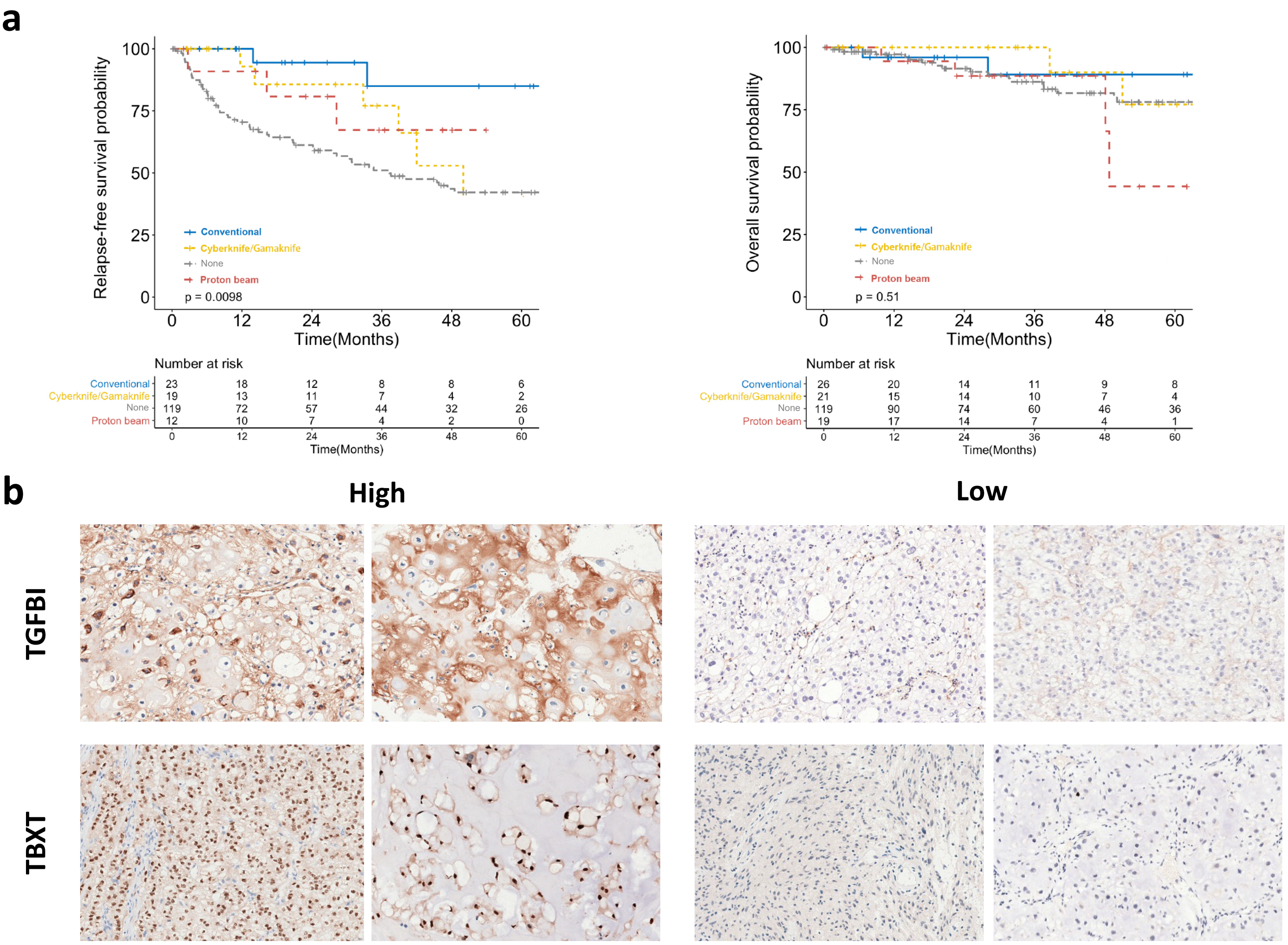

Supplementary Fig. 9 The baseline clinical feature and TGFBI and TBXT expression in 187 SBC cohort.
a) Patients who received proton/carbon beam radio surgery had worse progression free survival (PFS). b) The expression of TGFBI and TBXT were identified by IHC in 187 samples.
