# Supplementary material for: Single-cell transcriptome reveals cellular hierarchies and guides p-EMT-targeted trial in skull base chordoma
Source: Cell Discov. 2022 Sep 20;8:94. doi: 10.1038/s41421-022-00459-2 (PMC9489773; doi:10.1038/s41421-022-00459-2)
Supplement: Supplementary file 10 — Supplemental Fig S10 [file 41421_2022_459_MOESM10_ESM.pdf]

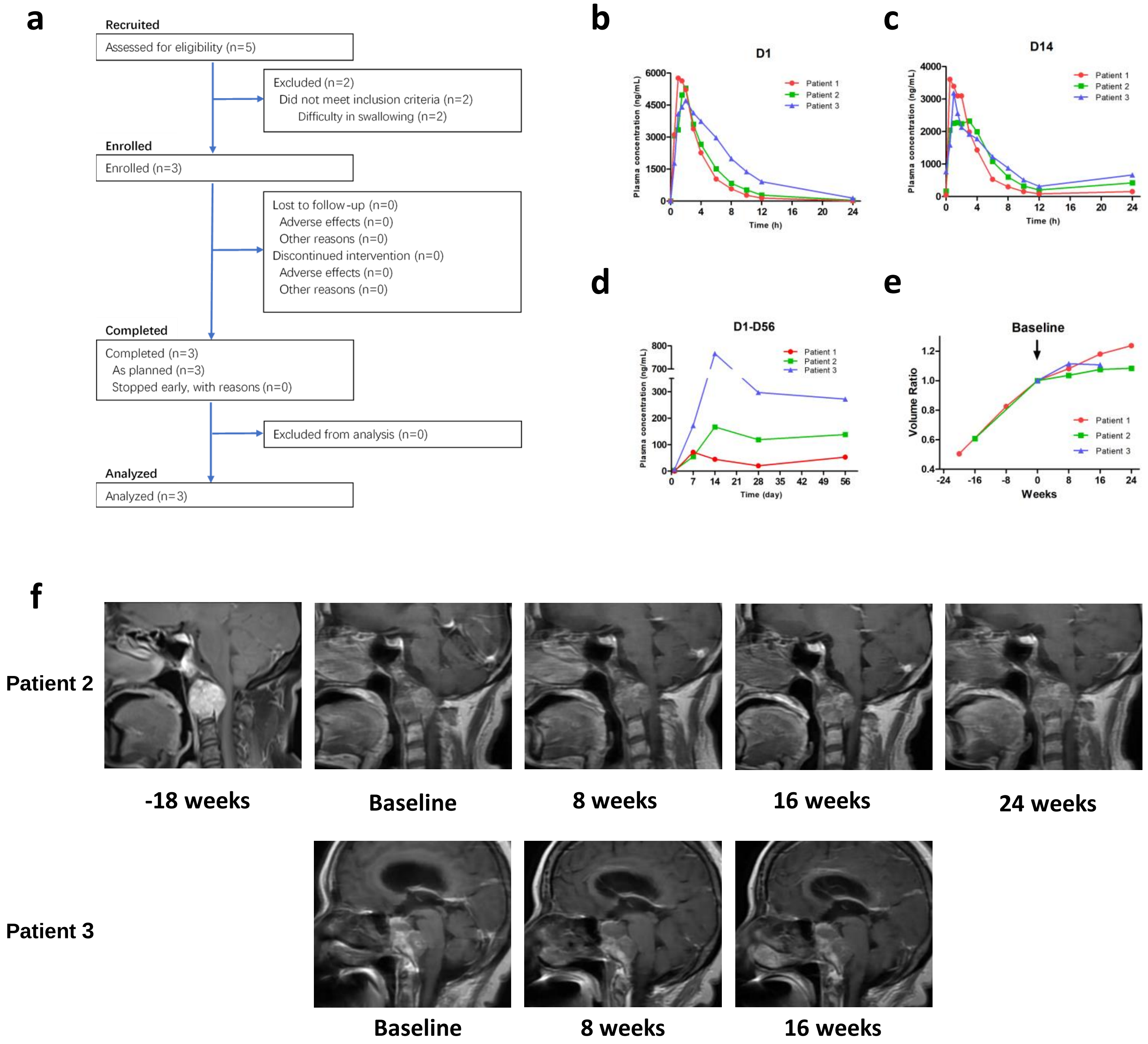

Supplementary Figure 10. Participant flow and outcomes of 3 SBC patients enrolled in Phase I clinical trial of YL-13027 . a) Diagram of participant flow. b-d) The pharmacokinetic profiles of all patients, including single-dose study (D1) and multiple-dose study (D14 and D1-56). e) Volume ratio change of 3 SBC patients. f) The MRI scan of Patient 2 and Patient 3 before and through the clinical trial.
