# Supplementary material for: Single-cell transcriptome reveals cellular hierarchies and guides p-EMT-targeted trial in skull base chordoma
Source: Cell Discov. 2022 Sep 20;8:94. doi: 10.1038/s41421-022-00459-2 (PMC9489773; doi:10.1038/s41421-022-00459-2)
Supplement: Supplementary file 11 — Supplemental Tab S1 [file 41421_2022_459_MOESM11_ESM.pdf]

Supplementary Table 1. Clinical information of the six SBC patients recruited for single-cell RNA sequencing.

Patient No.	Gender	Age	Course (months)	Tumor Diameter (cm)	Symptoms	Recurrence	Pre-op radiation	Region	Resection rate	Brachyury	Ki-67
1	M	32	1	4.5	Hoarseness and dysphagia	Primary	No	Clivus	Total	+	2%
2	M	32	6	5.8	Visual loss and dysphagia	Primary	No	Sellar	Sub-total	+	3%
3	F	63	1	3.0	Visual loss	Recurrence	No	Clivus	Sub-total	+	3%
4	M	43	3	3.9	Visual loss of right eye	Primary	No	Clivus+Sellar	Total	+	2%
5	F	71	5	3.7	Visual loss and blepharoptosis of left eye	Primary	No	Sellar	Sub-total	+	3%
6	M	22	2	4.8	Headache and diplopia	Primary	No	Clivus	Sub-total	+	3%
