# Supplementary material for: Single-cell transcriptome reveals cellular hierarchies and guides p-EMT-targeted trial in skull base chordoma
Source: Cell Discov. 2022 Sep 20;8:94. doi: 10.1038/s41421-022-00459-2 (PMC9489773; doi:10.1038/s41421-022-00459-2)
Supplement: Supplementary file 12 — Supplemental Tab S2 [file 41421_2022_459_MOESM12_ESM.pdf]

Supplementary Table 2. Top 100 marker genes of 17 clusters.

Cluster 1		Cluster 2		Cluster 3		Cluster 4		Cluster 5		Cluster 6		Cluster 7		Cluster 8		Cluster 9	
Gene 1-50	Gene 51-100	Gene 1-50	Gene 51-100	Gene 1-50	Gene 51-100	Gene 1-50	Gene 51-100	Gene 1-50	Gene 51-100	Gene 1-50	Gene 51-100	Gene 1-50	Gene 51-100	Gene 1-50	Gene 51-100	Gene 1-50	Gene 51-100
RPL37A	RPL5	ADIRF	CD99	MALAT1	SMCR5	HLA-DRA	NAMPT	FN1	EDIL3	RPL7A	IFT22	MGST1	AEBP1	H2AFZ	BOLA3	B2M	LTB
KRT19	RPL12	CLU	RPL35A	NEAT1	SLC25A27	CD74	SDCBP	MT-ND1	KRT8	EEF1B2	MRPL11	IGFBP7	RPS27L	STMN1	ZWINT	BTG1	TNFAIP3
EEF1A1	RPL24	HES1	RND3	MT-ND3	CALY	HLA-DRB1	CLEC7A	APP	CHST3	RPL13	IMPDH2	SERPINE2	TPI1	TUBA1B	RANBP1	TMSB4X	CST7
RPL13	SELENOM	EXOC1L	BHLHE40	MT-ND2	ZBTB20-A	TYROBP	INSIG1	MT-ND2	COL11A1	EEF1A1	SNHG7	IFITM3	SCCPDH	PTTG1	CDKN3	CXCR4	HLA-E
RPL37	UQCR10	IFT57	IGFBP2	DDX5	AL359382.	HLA-DPB1	OLR1	MT-ND4	LRP1	RPL19	HSPB2	CHI3L2	SQSTM1	GAPDH	SKA2	PTMA	CRIP1
TOMM7	SERF2	RPS19	IGFBP7	MT-CO2	AP001160.	HLA-DPA1	GLUL	MT-CO3	COBLL1	RPS18	LINC01003	SPP1	EIF4EBP1	PCLAF	CENPM	RPL21	RPL14
RPLP0	RPL13A	ARPC2	CST3	MT-ND4	CCNL1	FCER1G	HLA-DRB5	SPTBN1	MRC2	RPL30	CRIM1-DT	NNMT	CD59	PPIA	POLR2L	CD52	H3F3B
RPS18	RPL27	FNDC4	KRT10	SON	AC098850.	AIF1	LGMN	MT-ND3	GTF2I	RPS23	IGBP1	TMSB10	SELENOM	CKS1B	MYL6	RPS27	RPS25
RPL36	RPL23A	CYR61	PDGFRL	FN1	AL731577.	FTL	SPI1	DST	ATP9A	RPS2	OLA1	TGFBI	TMEM45A	TUBB	PRC1	CREM	CORO1A
RPL19	CD24	SNHG25	CIRBP	MT-CO1	AC020659.	HLA-DQB1	LY86	CD109	ATP2B4	RPL37A	HDDC2	FN1	TNFRSF1A	DTYMK	ANAPC11	RPS29	RPS16
RPS7	RPL39	CAV1	HOPX	WSB1	ARHGAP3	TMSB4X	FCGR3A	ACAN	GATAD1	RPS7	PSMG1	NDUFA4L2	ANXA2	BIRC5	S100A10	HLA-A	PPP2R5C
RPLP1	GAPDH	CD164	CXCL1	AKAP9	ASB15	NPC2	TREM2	COL2A1	HAPLN1	RPS6	BEX2	PLOD2	ACTG1	HINT1	COX8A	SRGN	STK17B
RPS6	CHCHD2	CEBPD	SPG21	DST	DLK1	HLA-DQA	LIPA	FMOD	MRGPRX3	NACA	DHRSX	TM4SF1	PKIG	RAN	ANLN	CD3D	GZMA
RPL9	SEC61G	TM4SF1	EEF1A1	MT-ND1	LINC02334	SAT1	CCL3L1	CRIM1	C20orf194	RPS13	MRPL12	FTH1	OCIAD2	UBE2S	TPX2	HLA-C	RPS10
RPS20	RPL34	RPL37A	RPL11	MT-CO3	AC023590.	C1QA	TYMP	CTGF	KCNQ1OT	RPS8	STOML2	PRSS23	SF3B6	RHEB	PTN	IL32	NKG7
OST4	RPL3	CD24	SNRPN	SPP1	AL022238.	HLA-DMA	MSR1	MFGE8	PLXNA2	MT1F	PITPNA-A	MDK	SEC61G	H2AFV	DEK	CCL5	FYN
RPS28	LGALS3	MINOS1	TCEAL8	AEBP1	AL031714.	C1QB	CCL4L2	IL6ST	ETV1	RPS4X	ARMC10	S100A16	CTSB	LSM4	TUBA1C	ARHGDIB	DDIT4
RPL38	EEF1B2	OST4	KCNMB4	MT-CYB	PURPL	MS4A6A	ITGB2	MT-ND5	CALU	RACK1	RPS5	C1R	CDK4	TMEM106	PFN1	S100A4	GMFG
RPL41	SNX3	AC004990.	TPM2	AC058791.	IGSF1	GPR183	CXCL8	EFEMP1	PAPSS2	RPL9	RSL1D1	SELENOP	MIF	RPA3	UQCRQ	MALAT1	SAMSN1
RPL30	HINT1	GADD45B	TRIB1	SLC25A37	POU1F1	FTH1	ARHGDIB	PLEKHH2	XYLT1	RPS21	SNRNP25	PKM	ID3	CENPX	CHCHD2	KLRB1	CD37
RPS2	ATP5MC2	PDE1A	GAS6	ACAN	FAM184B	LAPTM5	SGK1	ACSL3	TSC22D4	MRPL17	CLNS1A	RND3	TUBA1A	UBE2C	NUCB2	DUSP2	HLA-B
RPL35	ARPC2	RPS4Y1	RPS27A	HES1	NAV2-AS3	PSAP	IGSF6	MT-ATP6	RASD1	EPB41L4A	COA4	SOX4	TCEA3	TK1	CD24	RPL27A	ISG20
RPL31	CAV1	ST13	RAB20	MT-ND5	TENM1	MS4A7	S100A4	MT-CO2	RAB3B	RPL3	SPAG16	GADD45A	FTL	HMGN2	JPT1	RPL10	SPOCK2
RPL35A	RPS11	NEAT1	SERPINB1	AC020916.	AC007098.	RGS1	PTMA	PRELP	SLC38A2	PGM1	AC027644.	COL1A2	RRAD	MZT2B	ATP5MC3	RPL32	CD3G
RPS19	SNORC	RPL31	CAVIN3	PLEKHH2	AC104964.	C1QC	SH3BGRL3	COL6A3	SPTLC3	SNHG8	METTL23	LY6E	FAM3C	TYMS	KDELR2	ZFP36L2	IL2RG
RPS3A	RPS15A	TMEM70	AEBP1	CLU	PLA2G6	SRGN	NR4A2	DDR2	NEDD9	RPL37	MYL5	PLIN2	SLC2A1	TOP2A	CENPU	PTPRC	RPL15
RPL7A	RACK1	RPL41	ATP5MC2	FUS	AC092140.	CYBA	CYBB	TNS3	LAMA4	PDLIM1	MALSU1	SLC16A3	DAB2	LSM5	YWHAQ	RPS15A	CYTIP
RPS13	NUPR1	FXYD1	DMAC1	MT-ATP6	AC016831.	LYZ	GMFG	SLC4A11	GALNT3	RPL12	COA5	HTRA1	ANGPTL4	RPL39L	NUCKS1	RPS24	RPL34
RPL8	COX7A2L	SNHG9	POLE4	AC004990.	AC100830.	CTSB	CTSD	RRBP1	CD81	RPL18	RPS9	C1S	FAM20C	NUDT1	THY1	SH3BGRL3	MT-CO1
RPS8	MZT2B	HOTAIRM	MIR4458H	AHI1	AC005046.	CTSS	LITAF	MT-CO1	DPYSL2	RPS12	COPZ2	TCEAL9	SEM1	MAD2L1	SNRPG	RPS3	EEF1D
RPS14	RPS27	ZFP36	RAB38	KCNQ1OT	AC009120.	LST1	PHACTR1	SYNE1	OLFML2A	RPL5	GPER1	NUPR1	KDELR2	CENPF	COX7A2	HCST	PFN1
RPS23	ST13	VAMP5	DDIT4L	HNRNPH1	MIR222HG	APOC1	CSF1R	WWP2	KLF2	CFAP36	STK16	IFITM2	RPL41	IGFBP2	C12orf75	RPL28	CD96
RPL6	RPL36A	RPS7	ATRAID	CCNL2	TAGLN3	HLA-DMB	HCST	TRIL	ZKSCAN1	RPS3	PIGP	CD151	DSTN	GGH	NAA38	RGS1	DDX24
RPS12	RPL18	KLF5	COX7A2L	HNRNPA2	PCK1	B2M	CXCR4	PDK4	GOLGB1	MRPS33	EBNA1BP2	SLPI	EIF5B	PBK	CALM2	IL7R	CFL1
ADIRF	ATP5ME	MEAF6	GJA1	ABI2	AC211476.	CD14	CD86	CREB3L2	ROCK2	SWI5	FAM114A1	HILPDA	LSP1P5	ENO1	LGALS1	FAU	CD48
RPL22	RPS5	ZSCAN16-	RPS8	ITGA3	B3GAT2	RNASE6	FAM49B	SCARA5	RHOBTB3	SLC25A4	ORAI3	EFNA5	RAB1A	UBE2T	COX20	LEPROTL1	CTSW
HOPX	RPS26	GYPC	SPINT2	LUC7L3	Z99289.1	CST3	MS4A4A	MT-CYB	TPM1	DMAC1	SNHG18	LY96	PHLDA3	RARRES2	ITGB1BP1	RPL23A	RUNX3
RPS27A	RPL4	SNHG18	RHOBTB3	TM4SF1	AC008914.	ALOX5AP	APOE	IGFBP2	RAPGEF5	PDZK1IP1	MGARP	TPM2	LAPTM4A	CENPK	DYNLL1	RPL17	HMGB1
MINOS1	RPL26	RPS2	BEX3	HSF4	AL023584.	CD68	CEBPB	EMP2	PON2	KDELR3	HEBP1	REXO2	EIF5A	ATP5MF	MRPL51	CD69	EVL
RPS4X	MRPS33	MAFF	SELENOM	GOLGA8B	CYP3A5	COTL1	PLEK	ITGA1	PPFIBP1	COPRS	YIF1A	PA2G4	TFG	CALM3	CCNB2	TRBC1	LCK
COX7C	VAMP5	A1BG	TCEAL9	TPM1	VGF	CCL3	CTSZ	ABI3BP	COL11A2	NDUFAF2	C2orf74	GAS6	RCN1	MZT2A	NDUFB2	CD3E	CYBA
RPL18A	S100A1	RHOB	EIF3L	AL157938.	MST1	RNASET2	CD53	CSPG4	CALD1	KRT19	MORN2	CHMP4B	SPSB1	ANXA2	TMPO	RPLP2	ELF1
PDLIM1	NDUFC1	RAMP1	ACP1	PRL	ASB18	ACTB	H3F3A	SLC26A2	TXNIP	RPL8	TMEM60	RHOC	A1BG	CENPW	PSMA7	RPSA	CDC42SE2
NACA	UQCRH	SCRG1	PNKD	CCDC144A	OTOS	PLAUR	HLA-B	NORAD	GLG1	RPS3A	MZT2B	RAB13	SLC25A37	HMGB2	HSPE1	TRBC2	SUB1
RPL7	POLR2L	SLC25A6	MRPL33	STARD4-A	RIMS2	CD83	REL	ITGA3	IQGAP1	YBEY	TUSC1	HSPB1	MYL6B	TPI1	DHFR	TRAC	RAC2
RPS21	RPLP2	NUPR1	MRPL20	AC005726.	DEFA4	C1orf162	RAB31	C2orf40	SEMA3C	RPS14	TOMM22	PDGFRL	SRM	PLP2	CENPH	SARAF	FAM177A1
RPL11	RPL23	IRF1	PYURF	MIR7-3HG	FSIP2-AS1	ITM2B	CFL1	MT-ND4L	GALNT10	NUDT2	RPL36	YWHAQ	GNB2	CENPN	NME4	RPL36AL	CALM1
COX7A1	RPS9	COL1A2	SOX9	ZDHHC11	SYT4	CXCL16	NR4A3	SLC25A37	ATP1A1	ASB9	BNIP3	SOD2	HES1	CKS2	UBB	TSC22D3	MT-CYB
RPS15	SNHG25	SOCS3	CUTA	CHGB	ANXA1	ARL4C	DBI	LGALS3BP	AHNAK	NDUFA8	RPS28	CCDC80	CRLS1	NUSAP1	COMMD4	CD2	RPS14
RPS25	S100A13	ERLEC1	AC016831.5	NNAT	SCG3	RGS10	FCGRT	ESYT2	SEZ6L2	MRPS15	RPL24	CTSL	GOLM1	CCDC34	DCTN3	CD7	CLEC2D

Cluster 10		Cluster 11		Cluster 12		Cluster 13		Cluster 14		Cluster 15		Cluster 16		Cluster 17	
Gene 1-50	Gene 51-100	Gene 1-50	Gene 51-100	Gene 1-50	Gene 51-100	Gene 1-50	Gene 51-100	Gene 1-50	Gene 51-100	Gene 1-50	Gene 51-100	Gene 1-50	Gene 51-100	Gene 1-50	Gene 51-100
DCN	ANKH	IFI27	COL18A1	S100A9	PTPRC	CD74	IGHM	SLPI	ALDH2	TOMM7	RPS10	MZB1	XIST	HBB	OSBP2
LUM	UACA	IGFBP7	WWTR1	SRGN	EVI2B	HLA-DRA	RPL29	WFDC2	AKR1C3	MIA	RPS20	SSR4	EAF2	HBA2	YPEL4
COL1A2	ACTA2	VWF	DUSP6	S100A8	S100A12	HLA-DRB1	RPL18A	TACSTD2	NCOA7	S100A1	C2orf40	IGKC	POU2AF1	HBA1	SCGB1A1
COL3A1	ID3	FKBP1A	HMGB1	FTH1	SDCBP	RPL21	RPL34	AQP3	MT-ATP6	CD63	PON3	IGHG1	ICAM2	ALAS2	CTSE
COL1A1	DSTN	PECAM1	MEF2C	NAMPT	TNFAIP3	HLA-DPB1	RPL10	KRT17	SFN	RPL17	GAPDH	JCHAIN	PAIP2B	SNCA	IGF2BP2
MGP	MEG3	CRIP2	GSN	SAT1	GLUL	CXCR4	RPL13A	ELF3	BPIFB1	POLR2L	NDUFA7	IGHG3	HLA-DOB	SLC25A37	PHOSPHO1
TIMP1	NOTCH3	IGFBP4	MYL12A	H3F3A	RGS2	PTMA	RPL17	KRT7	SIX3	NDUFA4	MYL6	IGLC2	GNG7	HBM	GMPR
COL6A2	CLEC11A	TCF4	PODXL	CXCL8	BASP1	CD37	NR4A2	CP	PRSS22	MINOS1	NDUFA13	IGHG4	MAN1A1	AHSP	SPTB
CALD1	ITM2B	CD59	TMSB4X	LST1	AQP9	CD79A	BIRC3	CLDN4	MT1X	RPL41	UBL5	DERL3	EVI2B	HBD	RHD
PCOLCE	TMSB4X	HSPG2	SLC9A3R2	CTSS	TREM1	RPSA	PRKCB	ALDH1A1	CLDN10	ATP5MC2	NDUFB11	CD79A	MEI1	TRIM58	TMOD1
IGFBP7	ASPN	ENG	PCDH17	SOD2	SAMSN1	RPS27	RPS4X	CXCL17	TNFSF10	RNASE1	TMBIM4	IGLC3	SLPI	DCAF12	KRT1
BGN	IGFBP5	RAMP2	TAGLN2	BCL2A1	GMFG	RPS29	YWHAZ	F3	IER3	COX6A1	ATP5MC3	FKBP11	PECAM1	SLC4A1	RIPOR3
COL6A1	ACTG1	EGFL7	A2M	TYROBP	SH3BGRL3	RPL32	RPL9	AQP5	SOX2	MT1M	GSTP1	SEC11C	HSH2D	HEMGN	SLFN14
SPARC	SSPN	PTMA	ITGB1	ACTB	AC245128.	FAU	JCHAIN	ID1	PRRG4	KRT18	MYL12B	CD27	FAM30A	CA1	LINC00570
APOD	PTCH1	COL4A1	CALCRL	H3F3B	PTGS2	HLA-DPA1	MEF2C	AGR2	PPA1	RARRES2	COPS9	CYBA	PRDM1	TESC	TUBB1
VCAN	PRRX1	GNG11	HLA-A	G0S2	HLA-DRA	HLA-DQB1	ARHGDIB	TSC22D1	CYB5A	AKR1B10	RNF181	ITM2C	IGHA2	TENT5C	AL138900.3
SERPINF1	TFPI	SPARCL1	ZFP36L1	B2M	CLEC7A	IGKC	RPS25	TPT1	PLEKHS1	APOE	COX14	IGHA1	IGHM	HBQ1	SLC38A5
LGALS1	OLFML3	PLVAP	TIE1	PLAUR	PLEK	CD52	RPS3	CAPN13	CD55	UQCRQ	NDUFB8	CD38	ZBP1	MYL4	RNF182
GSN	PLAC9	NFIB	ANXA2	MNDA	RNF149	HLA-DQA	TNFRSF13	LCN2	EZR	RPL32	COX6B1	TNFRSF17	TNFRSF18	RBM38	SMIM5
IGFBP4	CAMK2N1	SPARC	IFITM2	CEBPB	IL1B	REL	HERPUD1	CD9	ACTG1	BEX3	ATP5MG	SPCS3	PNOC	C2orf40	ART4
PDGFRB	IFI27	COL4A2	MMP2	FTL	LCP2	CD83	HLA-B	VMO1	ECE1	RPS18	ATP5MD	XBP1	ITGB7	NFE2	KEL
TAGLN	PLS3	AQP1	MYL12B	LITAF	S100A11	LTB	RPL3	SERPINB3	KRT18	TMA7	MRPL51	FCRL5	RAB30	EPB42	DEFA3
SPON2	BICC1	ADGRL4	S1PR1	DUSP1	SLC11A1	LAPTM5	RPL26	RARRES1	MMP10	COX5B	GTF2A2	ISG20	TP53INP1	EPB41	BPIFB1
CDH11	ISLR	CLEC14A	STOM	FCER1G	FYB1	RPS3A	CMTM6	S100A6	A4GALT	COX7A2	ARL6IP4	HLA-C	SEL1L	IFIT1B	GPR146
MXRA8	MYL12A	IGFBP5	FOXP1	CSF3R	CYBA	RPS8	CD48	PLPP2	LAMB3	PFDN5	KRT8	FKBP2	U62317.4	IGLC2	EPYC
SFRP4	FHL1	CD93	GIMAP7	LAPTM5	OLR1	MS4A1	BCL11A	PITX1	ST6GALN	S100A13	RPL37A	SDC1	LY9	MBNL3	PAGE2B
C1R	IGFBP6	IFITM3	DNASE1L3	AIF1	HLA-E	MALAT1	RPS5	PERP	CAPN1	PPDPF	ELOB	SPCS2	RGS1	MXI1	XK
CTHRC1	PDLIM7	ECSCR	CCDC85B	S100A4	SMIM25	RPL27A	RPS12	NFIB	SAA1	SPG21	NDUFA11	PRDX4	TMEM156	DMTN	SERPINB3
NR2F2	TPM4	HLA-E	ECE1	NFKBIA	NCF2	RPS11	HLA-DQA	MPZL2	MEIS2	RHEB	CALM2	MANF	AQP3	TF	HEPACAM2
MYL9	ITGB1	MARCKS	TMSB10	C5AR1	S100A6	RPL39	IRF8	FXYD3	DHCR24	RPS26	TXN	JSRP1	FAM92B	GYPA	SLC2A4
CTSK	SERPINH1	TFF3	TSHZ2	LYZ	SLA	B2M	CORO1A	GSTP1	CTSD	NDUFA1	ANAPC16	SLAMF7	NCF4	FECH	ESPN
C11orf96	CFH	LDB2	RNASE1	HLA-B	SORL1	RPL11	PHACTR1	BST2	SPINT2	COX6C	SEC61G	HLA-B	LMAN1	AC092490.	CCL3L1
TPM2	LTBP2	TGM2	MGP	TMSB4X	IVNS1ABP	BTG1	CD79B	CYP4B1	CXADR	MT1E	UQCR11	SPAG4	AC007952.	SPTA1	KAT2B
THBS2	F2R	ID3	PLPP3	SERPINA1	TNFRSF1B	TMSB4X	RPL27	EPHX1	ADAM28	DYNLL1	NDUFAF3	PIM2	FCGR2B	E2F2	SLC6A8
FBLN1	FSTL1	HLA-B	TNFSF10	PHACTR1	CLEC4E	RPLP2	SPIB	SPINT1	PRDX1	COX7C	GADD45GI	HERPUD1	SCNN1B	LYL1	HBG2
FHL2	DIO2	HYAL2	SOX4	FPR1	CMTM6	CD69	LIMD2	MSLN	SORD	S100A11	PCBP2	IGHG2	IL2RG	GYPB	TFR2
PTMS	A2M	TGFBR2	PTMS	ITM2B	ATP2B1	RPS15A	PABPC1	AKR1C2	EHF	ATP5MF	ATP5IF1	TENT5C	S100B	KLF1	KCNH2
TSC22D1	CD63	EMCN	PTPRB	HLA-C	LYN	RPL23A	SRSF5	PRSS23	JUN	COX8A	RPL31	CRELD2	ADA2	BPIFA1	R3HDM4
EMILIN1	OLFML2B	TSC22D1	KTN1	MCL1	PTPRE	RPS23	MT-CO1	AKR1C1	HES4	GABARAP	RPL13A	BST2	ARSA	RHAG	SLC22A16
TUBA1A	C1S	INSR	ADGRF5	ALOX5AP	MAP3K8	BANK1	RPL19	RAB25	PHLDA2	NDUFB2	RPL12	CD74	VPREB3	TMCC2	FOXO4
NNMT	TIMP2	ELK3	CTNNB1	LUCAT1	OSM	RPS10	RPS6	LMO4	CLDN7	CA3	RPL35A	APOD	ST6GALN	AC100835.	TMEM86B
GGT5	CYP1B1	ESAM	CD34	CXCR4	OAZ1	GPR183	RPS13	BPIFA1	MDK	NUDT4	ATP5F1E	HSP90B1	KLF13	ANK1	AC007383.3
TCF4	TIMP3	PCAT19	HLA-C	FCN1	ACSL1	HLA-DMA	RPS27A	EPCAM	TSPAN13	COX7A1	RPS27L	BPIFA1	KCNN3	AC130456.	GSTM1
COL4A2	FOXS1	ID1	MGST2	IER3	SELL	EZR	IGLC2	SCPEP1	GABRP	C12orf57	MT2A	SDF2L1	TAGAP	TRIM10	IGKC
SERPING1	LMNA	CDH5	ARHGAP2	NEAT1	PPIF	RPL10A	POU2F2	ADH1C	IL1R1	NDUFS5	ITM2A	CYTIP	AC012236.	SLC14A1	AC092117.1
IFITM3	COL5A2	RAMP3	LMO2	COTL1	LSP1	RPL14	H3F3A	SERPINF1	FMO2	MT1X	CRYAB	CTSS	RHOH	TAL1	ADD2
LHFPL6	LTBP4	PALMD	GIMAP4	LCP1	ARPC5	RPL15	HLA-C	S100A2	CLDN1	RPS17	NAA38	CD79B	DUSP2	GATA1	CRB1
ITGBL1	PLTP	MARCKSL1	TUBA1A	MXD1	CD53	CYBA	EVI2B	MT-CYB	POR	S100B	RPS27A	SPCS1	BPIFB1	IGHG3	AQP10
MARCKS	LMO4	NPDC1	FILIP1	IFITM2	CSTA	RPS16	YPEL5	IFI27	FAM107B	RPL15	CNN3	SEL1L3	SPATS2	TMPRSS9	AC092167.1
COL4A1	VIM	MMRN2	PLXND1	BTG1	CD74	RPS24	HLA-DMB	MUC4	TSPAN1	OST4	BLOC1S1	ICAM3	DPEP1	TSPO2	FAM178B
