# Supplementary material for: Single-cell transcriptome reveals cellular hierarchies and guides p-EMT-targeted trial in skull base chordoma
Source: Cell Discov. 2022 Sep 20;8:94. doi: 10.1038/s41421-022-00459-2 (PMC9489773; doi:10.1038/s41421-022-00459-2)
Supplement: Supplementary file 13 — Supplemental Tab S3 [file 41421_2022_459_MOESM13_ESM.pdf]

Supplementary Table 3. Top 100 marker genes of six clusters of stromal cells.

Cluster 0		Cluster 1		Cluster 2		Cluster 3		Cluster 4		Cluster 5	
Gene 1-50	Gene 51-100	Gene 1-50	Gene 51-100	Gene 1-50	Gene 51-100	Gene 1-50	Gene 51-100	Gene 1-50	Gene 51-100	Gene 1-50	Gene 51-100
GRUNX2	S100A4	RPS18	RPL23	APOD	PHGDH	IGFBP7	COL3A1	FN1	LOXL1	RGS5	HLA-B
ITGA10	EML4	RPLP1	TOMM7	GSN	APOE	SFRP2	PLSCR4	POSTN	MEG3	COL18A1	OAZ2
ENPP2	CD9	RPL37A	S100A11	CLDN1	SYNPO2	NR2F2	MYL12B	COL6A1	RCN3	NOTCH3	ITGB1
MEF2C	THSD4	RPS12	RPL35A	GPC3	MRAS	FHL2	CRIM1	COL6A2	PKM	CALD1	MEF2C
RBP4	MT-CO1	RPS14	RPS23	DCN	SCN7A	ITGBL1	UACA	SERPINE1	PTGIS	PLXDC1	DLC1
MXRA8	CLMP	RPS6	RPL7	P2RY14	PLAC9	DCN	SYNPO2	COL6A3	CAV1	NDUFA4L2	CCDC3
EMP2	TSC22D3	RPL39	RPS15	RBP1	PHLDA3	VCAN	SSPN	LOXL2	PXDN	IGFBP7	EFHD1
OLFML3	ARL6IP5	GAPDH	RPS25	FOXS1	CXCL12	CFH	TIMP1	COL4A2	CAVIN1	COL4A2	EPAS1
IBSP	SATB2	RPL27	OST4	ITM2B	BTG1	MGP	PDLIM2	COL5A1	PFN1	ABCC9	MYH9
DLX5	CTSK	RPL37	FTL	ABCA8	PTCH1	IGFBP2	MATN3	COL1A2	HTRA1	MCAM	GUCY1B1
FGFR1	MAFB	RPS27A	RPL8	OAF	ISLR	BGN	SYNPO	ITGB1	NEAT1	COL4A1	CAV1
PPP1CB	CLU	RPS13	RPL6	SPARCL1	CLDN11	SFRP4	CD81	COL4A1	SLC20A1	B2M	EBF1
NCAM1	MALAT1	RPL27A	C2orf40	MGP	F3	C1R	FGF7	LOX	FKBP10	HES4	IL34
GMFG	EMID1	RPLP0	RPS7	C11orf96	CYP1B1	SPON2	FBLN5	SULF1	UGDH	PDGFRB	TFPI
OMD	RSPO3	RPL35	RPS27	MATN2	A2M	FRZB	IGFBP4	COL1A1	AEBP1	FRZB	ARHGAP29
COLEC12	CAMK2N1	RPL13	RPL3	TENM2	MARCKS	MARCKS	OGN	PDLIM3	SPHK1	TINAGL1	C11orf96
SPP1	SCARA3	RPS21	RPL24	LHFPL6	EMCN	GGT5	UBE2E2	SPARC	COL8A1	ACTA2	SEPTIN4
ENPP1	GPC1	KRT19	RPS4X	NR4A1	KIAA1755	SLC6A6	LHFPL6	THBS2	LRRFIP1	MYL9	TLN1
CDH11	AHNAK	RPL18A	RPL10	NR2F2	RNF24	CALD1	ABI3BP	CERCAM	KDELR2	A2M	SELENOW
TXNIP	FBLN1	RPL28	RPL29	PTGDS	ADH5	ISLR	TAGLN	TPM2	ACTA2	FILIP1L	EEF1D
CXCL14	TPST1	S100A10	FTH1	CRISPLD2	NDRG2	LRRC32	PHLDA3	CCDC80	GAP43	ITGA1	UACA
TSC22D1	DDX5	RPL19	RPL21	SOCS3	CH25H	EFEMP1	GUCY1A1	NTM	CD151	THY1	PPP1R15A
PTH1R	SDC1	RPLP2	PPIA	ZBTB20	AC116345.	TGM2	CAVIN2	CD44	PLOD2	KCNJ8	BCAM
NNMT	CTSD	RPL30	RPL31	PKDCC	IGFBP5	PRELP	PHACTR2	ACTN1	PSAP	CPE	RPS7
PMEPA1	FOXC2	RPS28	RPL7A	CEBPD	EZR	LTBP4	ENG	TAGLN	ANO1	CCDC102B	SLC25A5
PTCH1	SERPINF1	RPS19	TMSB10	ECM1	FOXD1	THBS2	MEG3	ACTB	CKAP4	SOD3	ARHGEF17
FLNB	TNFSF11	RPL23A	RPL14	LUM	JAM2	HCFC1R1	NRP1	COL5A2	CALU	CHCHD10	CYGB
DBI	MMP2	RPS2	RPSA	AKR1C3	GUCY1A1	COL8A1	AHR	CDH13	DCBLD2	TGFB1I1	GJA4
EMP3	CTNNB1	RPL38	SNORC	OLFML2B	C1S	AEBP1	CFI	SH3BGRL3	S100A11	ARHGDIB	BTG1
HMGB1	BICC1	RPL12	RPL10A	LAMA2	HIC1	GSN	FBLN1	COL3A1	P4HA2	PPP1R14A	LPP
SELENOP	HERPUD1	UBA52	RPL22	KLF9	STAT3	C1S	PDLIM3	TNFRSF12	MICAL2	HLA-C	ACTN4
KRT17	CADM1	RPL26	RPS16	H3F3B	MEOX2	F2R	CTSF	MYL9	ITGA5	PTMA	PPP1R12A
TMEM50A	EMP1	RPL34	RPL15	MARCKSL1	COLEC12	FIBIN	NID1	LTBP1	LGALS1	ISYNA1	ITGA7
PLEKHA5	PTMA	RPL36	TPT1	IGFBP4	IGFBP6	DIO2	PMP22	TIMP1	ZYX	EPS8	SGIP1
PCOLCE	ZFP36L2	SERF2	S100A6	BTG2	ETV1	CLDN11	PDGFRB	PRSS23	ANGPTL4	BGN	HLA-A
FAM118A	VAMP5	RPS20	RPL18	LMO4	ING1	PLXDC2	ITGA1	FBN1	CREB3L1	MALAT1	HSPB1
CRNDE	TANK	ATP5F1E	RPL11	JUN	MTUS1	RARRES2	PRRX1	CTSB	MYH9	PGF	APOE
PALLD	IFITM5	RPL41	NDUFA4	SSPN	TENT5A	ITM2B	MYLK	SLC16A3	ITGB5	GGT5	ADAMTS1
CDC42EP3	DPEP1	RPS29	MT2A	RGS16	XIST	CDH11	RCAN2	TIMP3	TAGLN2	SPARCL1	ID4
UNC5B	PNISR	RPS11	RPS8	NR4A2	PGF	LRP1	RRAS	EFEMP1	PHLDA2	TPM2	LTBP4
SDC2	LAPTM4A	RPS10	CHCHD2	FZD2	SOX9	FHL1	FILIP1L	IGFBP2	AP3S1	SEPTIN7	TNS1
ZFP36L1	RSRP1	RPL32	RPL36A	CCL2	OGN	CCL26	NOTCH3	FLNA	PLAUR	TPPP3	HIGD1B
ZFHX4	MPP6	RPL13A	RPS5	ITGB4	FZD1	GAS6	CD14	SERPINH1	NDUFA4L2	CD59	STOM
FXYD6	FOS	RPS15A	LGALS3	CFI	EBF2	RFTN1	LTBP2	P4HB	BGN	CYTOR	GPC3
NDNF	ANXA1	RPS3A	RPS3	JUNB	YBX3	CTSC	DKK3	CTHRC1	PDLIM4	TAGLN	MYH11
H3F3A	FOSB	EEF1A1	PFDN5	TCF4	WWTR1	FMO2	ACTA2	ANXA2	WDR1	MGLL	TMSB4X
LEF1	AC020916.	FN1	RACK1	DIO2	EGR3	SMOC2	ANGPT1	S100A16	F2R	LHFPL6	LRRC32
IFITM2	SOX4	RPL9	TF	GADD45A	NFIA	LUM	SEPTIN11	HSPG2	TPM1	KCNE4	SFTA1P
RAD21	COL10A1	RPS24	POLR2L	HILPDA	PRELP	MEST	ID3	FSTL1	CLIC4	GUCY1A1	EHD2
NR3C1	SEPTIN11	EEF1B2	FAU	SLC2A3	SERPINF1	CTGF	ZMAT3	LTBP2	ERRFI1	MYLK	SH3BGRL
