# Supplementary material for: Single-cell transcriptome reveals cellular hierarchies and guides p-EMT-targeted trial in skull base chordoma
Source: Cell Discov. 2022 Sep 20;8:94. doi: 10.1038/s41421-022-00459-2 (PMC9489773; doi:10.1038/s41421-022-00459-2)
Supplement: Supplementary file 14 — Supplemental Tab S4 [file 41421_2022_459_MOESM14_ESM.pdf]

Supplementary Table 4. Top 100 marker genes of 15 clusters of immune cells.

Cluster 0		Cluster 1		Cluster 2		Cluster 3		Cluster 4		Cluster 5		Cluster 6		Cluster 7	
Gene 1-50	Gene 51-100	Gene 1-50	Gene 51-100	Gene 1-50	Gene 51-100	Gene 1-50	Gene 51-100	Gene 1-50	Gene 51-100	Gene 1-50	Gene 51-100	Gene 1-50	Gene 51-100	Gene 1-50	Gene 51-100
RPLP1	RPS20	FTL	CXCL16	S100A9	GCA	CCL5	LEPROTL1	NKG7	MATK	C1QB	FCER1G	HLA-DPB1	PPT1	MZB1	PIM2
RPS27	RPL35	C1QA	RNASET2	S100A8	THBS1	NKG7	PPP2R5C	GNLY	PPP2R5C	C1QA	S100B	HLA-DRA	STX11	SSR4	SCRG1
RPL10	RPS8	CTSB	A2M	NAMPT	ITM2B	RPS27	UBB	GZMB	SAMD3	FTL	GAPDH	HLA-DQB1	PLSCR1	IGKC	SLAMF7
RPL32	RPS5	NPC2	ASAH1	NEAT1	TIMP1	GZMK	CCL4	CCL5	DDIT4	HLA-DPA1	TUBA1B	CD74	GPAT3	IGHG1	BPIFA1
RPL41	TSC22D3	PSAP	CEBPB	SOD2	RNF149	CD3D	RPLP1	KLRD1	SRSF2	KRT19	RPL30	HLA-DPA1	EMP3	JCHAIN	MYDGF
CD3D	TRAC	CD74	HMOX1	SAT1	RGS2	CST7	CMC1	CST7	EVL	CST3	FGL2	HLA-DQA	NPC2	IGHG3	ICAM3
RPL21	RPL12	C1QB	CCL3L1	H3F3A	SDCBP	MALAT1	ITM2A	PRF1	RUNX3	HLA-DRA	EEF1B2	HLA-DRB1	ACTB	IGLC2	RPN2
RPS25	RPL30	HLA-DRA	LY96	FTH1	CLEC4E	RPL41	TRBC1	CTSW	SYTL3	HLA-DPB1	CD68	INSIG1	GABARAP	IGHG4	TMEM59
RPS19	PTMA	HLA-DRB1	GPR183	SRGN	IVNS1ABP	CXCR4	FAU	FGFBP2	PYHIN1	APOE	RPS11	CST3	RILPL2	DERL3	KDELR1
RPS18	RPL27	C1QC	FCGR3A	CXCL8	SMIM25	DUSP2	RPL18A	MALAT1	RARRES3	C1QC	ARPC3	HLA-DMA	YBX1	CD79A	RABAC1
RPS3	RPL36	TYROBP	CCL4L2	LST1	VCAN	RPS29	CD7	GZMH	AKNA	CD74	RPS23	LYZ	DBI	FKBP11	SRPRB
IL7R	RPL19	CTSD	ITM2B	CTSS	FOS	RPL28	FYN	HLA-C	C12orf75	FN1	SLC40A1	GPR183	NR4A3	PRDX4	TPD52
RPS15A	CD3E	MS4A7	CSF1R	BCL2A1	PPIF	GZMA	CD52	GZMA	FYN	AIF1	TMEM176	FCER1A	RPS3A	SEC11C	HERPUD1
RPL23A	EEF1D	APOC1	DAB2	CEBPB	NCF2	TUBA4A	CD3G	CD247	MT-CO2	TPT1	CTSB	LST1	TYROBP	ITM2C	SDF2L1
RPS27A	RPL18	FCER1G	RNASE6	G0S2	ACSL1	RPL23A	ISG20	KLRB1	EIF1	HLA-DRB1	FOLR2	HLA-DQA	RPS9	IGLC3	ICAM2
RPS12	RPS28	APOE	TYMP	PLAUR	S100A4	IL32	RPS27A	CMC1	CD69	SELENOP	RNASE1	AREG	RPS16	APOD	CHID1
RPLP2	B2M	CD14	CD63	S100A11	CLEC7A	RPS15A	RPS19	CD7	SRSF7	HLA-DQA	CTSC	TMSB10	LSP1	TF	SELENOS
RPL28	CD2	CST3	PLIN2	MNDA	CYP1B1	B2M	YPEL5	HLA-A	SYNE2	CD63	IFITM3	CD1C	JAML	S100B	TENT5C
RPS14	TRBC2	LGMN	MS4A4A	CSF3R	CD55	BTG1	TRGC2	KLRF1	RPL14	LGALS3	RPLP0	CLEC10A	LY86	XBP1	MRGPRX3
KLRB1	RPS3A	SPP1	TGFBI	SERPINA1	COTL1	RPL13A	DDX24	GZMM	RSRP1	C2orf40	CSTB	C1orf162	OLR1	CD27	SEL1L
RPL13A	RPL36A	SAT1	CREG1	DUSP1	LCP1	RPS3	RPL32	RPS3	KRT19	SNORC	GSTP1	C15orf48	RPS23	FKBP2	MT1E
RPL34	RPL26	HLA-DPB1	STAB1	LYZ	LAPTM5	HLA-A	CNOT6L	CCL4	PTPN7	EEF1A1	MT2A	SAT1	SLC25A5	SLPI	DERL1
IL32	RPL15	CTSZ	MFSD1	IER3	ARPC5	RPL27A	CD2	CLIC3	ABHD17A	MARCKS	TMSB10	LGALS2	RNASET2	SNORC	PDLIM1
RPS29	ITM2A	HLA-DQB1	NEAT1	BASP1	IL1B	HLA-C	RPL30	TRBC1	MT-ATP6	S100A11	RPS13	COTL1	ALOX5AP	SPAG4	RAB30
RPL13	FAU	HLA-DQA	RGS1	C5AR1	MAP3K8	CD8A	EEF1D	B2M	SH2D2A	RPL37A	MS4A7	PPA1	H2AFY	IGHA1	CLPTM1L
CD52	RPS17	HLA-DPA1	CXCL8	S100A6	LCP2	RPS25	SRSF2	TRDC	BIN2	HLA-DQB1	S100A10	CD1E	RALA	SELENOM	TMED9
RPS10	MALAT1	CCL3	OLR1	FCN1	OLR1	RPLP2	FAM177A1	DUSP2	VAMP2	TYROBP	A2M	AP1S2	GSN	SDC1	C1QTNF3
RPL27A	RPL8	AIF1	RNASE1	SLC25A37	VMP1	RPL21	DUSP4	HLA-E	RPL30	NACA	SERF2	HLA-DMB	RGS10	TNFRSF17	PAIP2B
RPL3	FAM177A1	SGK1	KLF6	ACTB	EVI2B	RPS18	RPS10	CALM1	RPL21	CTGF	GRN	CD86	GDI2	HSP90B1	TMEM208
ZFP36L2	RPL24	FCGRT	CYBB	FPR1	CSTA	PTMA	DNAJB1	PTMA	LIMD2	FCGRT	VAMP8	RNASE6	PHACTR1	FCRL5	CD79B
LEPROTL1	CRIP1	MS4A6A	CLEC7A	NFKBIA	PLEK	RPS14	ARHGDIB	IFITM2	SEPTIN7	ITM2B	IFI27	SERPINB9	SEC11A	NUCB2	CTHRC1
RPS6	SPOCK2	CD68	LY86	LUCAT1	SELL	CD3E	HCST	IRF1	IFITM1	NPC2	ANXA5	CSF2RA	REL	CD38	ST6GALNAC4
BTG1	RPL6	MARCKS	HES1	MXD1	IFITM3	CALM1	RPL31	RPL3	LDHA	VIM	RPL9	MS4A6A	HERPUD1	SPCS2	RAB3B
RPS16	RORA	HLA-DMA	NAMPT	LITAF	BRI3	RPL13	PTPRC	UBB	PAXX	CCL3L1	CTSL	RPS24	PPIF	C2orf40	EAF2
RPL11	RPL7	HLA-DRB5	MEF2C	PHACTR1	OSM	RPL3	RPS7	RPS27	C1orf21	CTSD	ATP5F1E	PKIB	KLF4	CRELD2	P4HB
RPL18A	ARHGDIB	LIPA	APLP2	H3F3B	SORL1	ZFP36L2	CD96	HLA-B	DDX5	RPL7A	RPL23	FCER1G	IFI30	JSRP1	DNAJB9
RPL17	RPL38	TREM2	MAFB	TYROBP	CMTM2	RPS2	RPL36AL	HCST	LINC01871	CD14	RPS24	AXL	NEAT1	PLPP5	DNAJC1
RPL31	NDUFS5	CTSL	SOD2	IFITM2	LYN	CD8B	RNF19A	SPON2	KLRC1	CCL4L2	HLA-DRB5	H3F3A	FTH1	SPCS3	MEI1
RPL35A	RPLP0	GPNMB	TIMP2	ASAH1	ATP2B1	TRAC	RPL34	XCL2	IL32	FCGR3A	LGMN	NAP1L1	DUSP4	SPCS1	SPATS2
RPL10A	CD3G	MSR1	KLF4	S100A12	PLSCR1	GZMH	TMSB4X	IER2	S1PR5	CCL3	ESYT2	CXCL16	RPL15	LMAN1	TMEM205
RPS4X	RPL36AL	CD9	LAPTM5	GLUL	ATP6V0B	TRBC2	SLC38A1	TUBA4A	BTN3A2	TF	HLA-DQA	RPS11	BTF3	ERLEC1	HM13
CXCR4	CD69	CD83	ADAP2	TREM1	C15orf48	RPS15	CLEC2D	BTG1	TRGC1	MS4A6A	YBX1	AIF1	RPS2	TXNDC15	DDOST
RPS2	RPS7	GLUL	SLCO2B1	FTL	TRIB1	RPS12	RPL17	RPL23A	ID2	CTSZ	APOD	TIMP1	ALDH2	PDIA6	GADD45A
LTB	RPL9	GRN	CD81	FCER1G	ANP32A	RPL10	RPS20	HOPX	DSTN	PSAP	OAZ1	BID	EIF3L	MANF	ARSA
SARAF	DDX24	IER3	RGS10	AQP9	PTPRE	RPL35A	RPL14	APOBEC3G	RPS7	APOC1	SLPI	BASP1	SNX3	LGALS3	POU2AF1
RPS15	TRBC1	FCGR2A	OGFRL1	AC245128.	MX2	SRSF7	SUB1	RPS27A	MYL12A	PRDX1	MS4A4A	ATP1B3	CLEC7A	KRT19	TP53INP1
CREM	RPS13	HLA-DMB	GSN	AIF1	CMTM6	GZMM	CD69	CXCR4	RAC2	HLA-DMA	IGKC	CTSH	ARL4C	IGHG2	ANKRD28
RPSA	RPL5	PLAUR	QKI	MCL1	LRRK2	CTSW	RPS28	CHST12	HMGB1	BRI3	PPIA	SLC25A6	RPL23	SSR3	COMMD3
RPL39	CALM1	HIF1A	SDCBP	PTGS2	ALOX5AP	EIF1	PIK3R1	IL2RB	SLC38A1	RPL37	PDLIM1	FCGR2B	TMSB4X	RRBP1	KRT18
RPL14	CD96	LGALS1	DBI	SLC11A1	CLEC12A	CREM	RPL15	PLAC8	RPS14	ACTG1	CTSS	LIMS1	CEBPD	LMAN2	SRM

Cluster 8		Cluster 9		Cluster 10		Cluster 11		Cluster 12		Cluster 13		Cluster 14	
Gene 1-50	Gene 51-100	Gene 1-50	Gene 51-100	Gene 1-50	Gene 51-100	Gene 1-50	Gene 51-100	Gene 1-50	Gene 51-100	Gene 1-50	Gene 51-100	Gene 1-50	Gene 51-100
IL32	EEF1D	STMN1	TUBA1C	CD79A	RPS2	KLRB1	RPS15A	TPSB2	HDC	JCHAIN	SPIB	ACP5	FUCA2
BATF	RPL28	TUBA1B	ANAPC11	RPS8	LTB	XCL2	PTMA	TPSAB1	RPL26	SEC61B	SRP14	GRN	SHTN1
TIGIT	TNFRSF9	H2AFZ	MAD2L1	MS4A1	CD52	TNFRSF18	RPS18	CPA3	TSC22D1	C12orf75	HINT1	GNS	CYSTM1
B2M	DDX24	PCLAF	YWHAH	RPS23	RPS25	LTB	RPL31	HPGDS	JUND	SOX4	RPS9	TCIRG1	NENF
TRAC	CYTIP	HMGN2	LGALS1	RPS11	RPS28	RPLP1	IL7R	MS4A2	PTMA	GZMB	FYTTD1	CD84	PRDX3
HLA-A	CALM1	HMGB1	DTYMK	RPL11	RPS15A	XCL1	RPL35A	LAPTM4A	MT-CYB	GPR183	AKAP13	CD68	ATP6V1F
CD3D	GADD45A	TUBB	ENO1	CD37	EEF1B2	KRT86	RPL41	RHEX	RPL14	LDLRAD4	HERPUD1	TIMP2	AKR1A1
LTB	RASGRP1	CKS1B	VIM	RPL13A	RPL27A	CTSW	IL4I1	ANXA1	FOSB	IGKC	APP	CHCHD10	GDI2
CARD16	PTPRC	HIST1H4C	ARL6IP1	RPS27	RPL12	CD52	TRBC1	NFKBIA	RPL30	IRF4	GPR65	TKT	OLA1
S100A4	CIRBP	HMGB2	ALOX5AP	RPL32	RPL35A	ID2	RORA	LTC4S	LMO4	SERPINF1	CREM	PCBD1	PGAM1
TNFRSF4	ICAM3	H2AFV	ANP32E	BANK1	CD69	RPL13	ZBTB16	RPS4X	RPS11	PLAC8	DSTN	PHB2	ATP6V1H
TNFRSF18	BIRC3	TYMS	CARHSP1	RPS3A	REL	AREG	RPL29	GATA2	RPL23	RPS3A	BCL11A	MMP14	NDUFS4
CD2	RPL36AL	NUCKS1	DBI	RPL39	RPS3	EEF1A1	RPS10	IL1RL1	RPS23	AREG	RPS4X	CTSZ	SDHD
BTG1	RPS3	TOP2A	CKLF	IGKC	RPL22	RPL6	RPS8	CTSG	STX3	TCF4	SLC20A1	MMP9	NDUFA8
TRBC2	GLRX	CFL1	COTL1	RPL8	CD79B	RPS24	DDIT4	CD63	NACA	RPS11	SSR4	RGS10	UQCRC2
PMAIP1	UQCRB	BIRC5	SMC4	RPL18A	HLA-DRA	RPL15	CD96	H3F3B	ID2	ALOX5AP	CORO1C	SPI1	SERPINB6
ARID5B	SUB1	PTTG1	ZWINT	RPL13	RPL14	RPL17	TRDC	VIM	RPL5	EZR	RGS2	TMEM176	PLBD1
LINC01943	PHTF2	UBE2C	IDH2	RPS5	RPL15	SKP1	RPL18	HPGD	LAT	SLC15A4	RNASE6	PLD3	PLIN3
CD27	ATP5IF1	DEK	TMPO	RPL19	RPL37A	UBB	RPL14	MT-CO2	EEF1A1	NR3C1	ETV3	ATP6AP1	ETFB
DUSP4	RPL3	TK1	AP2S1	RPS27A	RPL36	RPL10	RPL4	RPL36AL	ACSL4	RPS23	DERL3	NDUFA10	PPP1R14B
CD3E	AC133644.	CDK1	SNRPF	RPL30	EZR	RPS4X	HLA-A	RPL24	PFDN5	IRF8	EEF1B2	VDAC2	CCT3
ICOS	LINC02195	PTMA	PCNA	RPS6	RPS7	TNFRSF4	SPRY1	MT-ND4	RPL7A	PPP1R14B	RPL10A	SLC25A11	IDH3G
TSC22D3	SAMSN1	DUT	CAPG	RPSA	IGLC2	RPL5	TNFRSF25	FTH1	NFKBIZ	CD74	RNASET2	VIM	TUBA1C
MALAT1	ETS1	ANP32B	GGH	RPL21	RPL41	RPS27A	ZFP36L1	UBB	RPL22	PLD4	SEL1L3	NDUFB5	NDUFS2
TRBC1	RTKN2	SLC25A5	CALM3	RPL9	VPREB3	CCDC107	PABPC1	RGS13	MT-ND2	TXN	RPS2	RALA	GLRX
UBC	GATA3	NUSAP1	ERH	CD83	RPL27	RPL8	LST1	LMNA	GPR65	PTGDS	RPLP0	IL13RA1	ACADVL
IL2RA	CLEC2D	ACTB	BANF1	RPLP2	MALAT1	RPL10A	TNFSF14	CD69	DDIT4	LILRA4	AP3S1	CST3	NDUFV1
CD52	UGP2	GAPDH	TMEM14C	RPL18	RPL7A	KIT	RPS12	CLIC1	RPL32	RPS8	SELL	CYC1	BLVRA
FOXP3	C15orf53	PFN1	AP1S2	RPL37	RPL38	RPL7	EML4	AREG	RPS24	CLIC3	CHAF1A	CTSD	MGST3
LAIR2	ITM2A	RAN	BLOC1S1	CD74	LINC00926	FAM177A1	RPLP0	S100A6	RPL10	TSPAN13	HLA-DPB1	TWF2	CCR1
CD7	RPSA	COX8A	SIVA1	RPL3	RPS14	RPL3	HNRNPA0	KIT	RAB27B	RUBCN	FKBP2	UNC93B1	CORO1B
SPOCK2	YWHAB	RANBP1	CENPM	RPL10A	RPL10	RPL32	CREM	RPL11	NDUFA4	SPCS1	C12orf45	NDUFS8	PDE4DIP
CTSC	IKZF2	PPIA	RHOA	IGHM	RPL6	HNRNPA1	RACK1	FAU	MT-ND5	IRF7	YWHAZ	MCRIP2	HNMT
HLA-C	CD247	YBX1	HMGN3	RPL23A	RPS15	IL2RG	FNBP1	MT-ATP6	LEO1	ANKRD11	PARK7	COMT	CYFIP1
RGS1	PHLDA1	LSM5	GNG5	RPS12	RPS16	RPS17	GPR65	CD9	ALOX5AP	SLC7A5	EGLN3	PTMS	ATP5F1C
TBC1D4	RPS15	LSM4	SNRPE	RPS4X	RALGPS2	IL32	SCX	AL157895.	BST2	MYL12A	RPS12	NECTIN2	FAM162A
CYTOR	MIR4435-	RPA3	LSM3	EEF1A1	RPL7	RPSA	RPL13A	MALAT1	FOXP1	ITM2C	CYBA	TEX264	CD4
IL2RG	LAT	HMGN1	NPC2	RPS20	HLA-DRB1	RPS5	SRSF2	VWA5A	HSP90AB1	PLP2	RPS13	GNPTAB	TXN2
CTLA4	PIK3IP1	MKI67	SNRPG	RPS29	POU2F2	TMIGD2	RPL24	CAPG	RPL6	NR4A3	MALT1	UQCRC1	SPAG7
CD3G	FYN	CKS2	ATP5PF	RPS18	PRDM2	FXYD5	NSMCE1	PPP1R15A	CKLF	HSP90B1	THBD	RASSF4	TBXAS1
ISG20	LAYN	TMSB4X	H2AFY	FAU	RPS9	NCR3	SRSF5	SELENOK	KDM6B	BID	LILRB4	NRP2	ATP5F1B
RPL24	RPL11	CENPW	RBX1	RPL29	MT-ND4	CD7	RPL9	MT-ND3	NR4A1	EEF1A1	ZC3HAV1	NDUFS3	CSTB
SOD1	RORA	CENPF	CENPN	RPS10	NOP53	AQP3	RPS23	GLUL	RACK1	IL3RA	ARL4C	CSF1R	ARF3
LCK	HSPA8	HNRNPA1	HPRT1	RPL5	CD55	RPL34	CXCR4	RPS27A	RPL29	SELENOS	ARID3A	ATOX1	LEPROT
CORO1B	BTG3	TPI1	PTMS	BIRC3	BCL11A	KRT81	NINJ1	RPL34	DUSP6	RPL6	SNX3	PRCP	REPIN1
STK17B	GBP5	PKM	HNRNPAB	RPS13	PNISR	SPOCK2	RPL19	BIRC3	IER2	MZB1	SUB1	HEXB	FCGRT
PBXIP1	CREM	RRM2	PGAM1	TNFRSF13	RPL23	RPS3A	SON	SLC18A2	TMEM176	RPL23	RPS7	TIMM8B	APLP2
ARHGDIB	MAST4	SMC2	MZT2A	RPS21	LY9	RPS6	PRMT9	CLU	GALC	IRF2BP2	HLA-DRA	ATP6V1A	KDELR1
IL2RB	FYB1	NUDT1	VPS29	RPL34	HLA-DPB1	RPL11	RPL23A	PRDX6	BTG2	PTPRE	SIDT1	GSN	HCFC1R1
SKAP1	RAC2	MZT2B	COX5A	RPL26	HLA-DQB1	DLL1	RPL26	PEBP1	MAOB	N4BP2L1	PRKCB	SNX5	EIF3D
