# Supplementary material for: Single-cell transcriptome reveals cellular hierarchies and guides p-EMT-targeted trial in skull base chordoma
Source: Cell Discov. 2022 Sep 20;8:94. doi: 10.1038/s41421-022-00459-2 (PMC9489773; doi:10.1038/s41421-022-00459-2)
Supplement: Supplementary file 15 — Supplemental Tab S5 [file 41421_2022_459_MOESM15_ESM.pdf]

Supplementary Table 5. 15 clusters of immune cells were further classified into eight cell types according to the marker genes listed above.

cluster	cell type	gene list
0	conventional CD4+T-helper cells (CD4+ T_{CONV})_RP high	TCF7, CCR7, CD48
1	non-classic monocyte 1	CD14, FCGR3A (CD16), ALDH1A1
2	neutrophil	MME (CD10), CEACAM8 (CD66b)
3	cytotoxic CD8+ T cell populations (CD8+ T)	GZMK, GZMA, GZMH
4	NK cells	NCAM1 (CD56), FCGR3A (CD16)
5	non-classic monocyte 2	CD14, FCGR3A (CD16), ALDH1A1
6	myeloid DCs	PKIB, ENHO, CD1E
7	plasma cells	IGHA1, IGHG3, CD38
8	regulatory T cells (Tregs)	FOXP3, IL2RA (CD25)
9	MKI67+ classic monocyte CD14	low FCGR3A (CD16), MKI67, PCNA
10	B cells	CD19, MS4A1 (CD20)
11	conventional CD4+T-helper cells (CD4+ T_{CONV})	TCF7, CCR7, CD48
12	basophil	FCER1A, CPA3
13	plasmacytoid DCs	LRRC26, SCT, SHD
14	macrophage	CSF1R
