# Supplementary material for: Single-cell transcriptome reveals cellular hierarchies and guides p-EMT-targeted trial in skull base chordoma
Source: Cell Discov. 2022 Sep 20;8:94. doi: 10.1038/s41421-022-00459-2 (PMC9489773; doi:10.1038/s41421-022-00459-2)
Supplement: Supplementary file 16 — Supplemental Tab S6 [file 41421_2022_459_MOESM16_ESM.pdf]

Supplementary Table 6. The top 100 marker genes of six malignant cell clusters.

Cluster 1		Cluster 2		Cluster 3		Cluster 4		Cluster 5		Cluster 6	
Gene 1-50	Gene 51-100	Gene 1-50	Gene 51-100	Gene 1-50	Gene 51-100	Gene 1-50	Gene 51-100	Gene 1-50	Gene 51-100	Gene 1-50	Gene 51-100
RPS4Y1	RPL19	HLA-DRA	POLR2L	MALAT1	ARID1B	COL1A1	MT2A	PCLAF	HELLS	PTTG1	MKI67
RPS3	CLU	S100B	COX7B	NEAT1	MMP19	CHI3L1	CLEC2B	PCNA	CARHSP1	H2AFZ	PFN1
RPS8	CAV1	APOD	KRT18	AC058791.1	ANGPTL4	RRAD	CEBPB	H2AFZ	C19orf48	STMN1	LSM4
ADIRF	RPL23A	SLPI	VDAC1	WSB1	MT-CYB	FTH1	MARCKS	TYMS	RPN2	UBE2S	CDK1
RPL31	MIA	CD74	PDCD6	AC020916.1	WDR60	SERPINE1	CD44	STMN1	CENPU	TUBA1B	GGH
RPS19	RPL9	S100A10	HSBP1	HNRNPH1	CDK5RAP3	SERPINE2	GDF15	RPA3	SLC25A5	TOP2A	CENPX
RPS28	FAU	PLAC8	ADH5	SON	QTRT1	TGFBI	IFI6	TUBA1B	PLP2	CENPF	DBI
RPS17	RPL8	TMSB4X	PAPSS2	MT-ND2	LINC01116	COL1A2	SH3BGRL3	CENPX	NME1	ARL6IP1	TAGLN2
RPL13A	RPL4	HLA-DPB1	TPD52L1	AKAP9	BRD9	IGFBP7	CALD1	GINS2	MCM6	BIRC5	NUDT1
RPL35A	RPS13	CALM1	PAIP2	MT-ND3	CHD9	NNMT	EMP3	DUT	POLR2L	HIST1H4C	PLP2
RPS6	RPL3	MYL12B	NDUFB3	FUS	MT-ND1	PLIN2	RHOC	MCM7	SLFN13	HMGB2	TPI1
RPS5	RACK1	PLAC9	LRRC75A	ABI2	MUC20-OT1	CHI3L2	MGST1	TK1	ZNF367	CKS2	TYMS
RPL5	RPS29	RPS24	SNORC	DDX5	NKTR	TUBA1A	NPC2	MCM3	CDT1	UBE2C	DEK
RPL34	RPL32	CSTB	AZIN1	MT-CO2	ACE	HMOX1	SPARC	THY1	PRKDC	HMGN2	ENO1
RPL41	RPL39	PSMB4	PPP1R3C	MT-ND4	ITGA5	CTSB	BGN	FGL2	SRGN	JPT1	TK1
RPS25	RPS4X	HLA-DRB1	PHB	MT-CO1	STAT3	PKM	TFPI	ESYT2	ZWINT	TUBB	EMP3
RPS15A	RPS16	LGALS3	H2AFJ	CCNL1	EMP1	TMSB4X	CFH	MT-ATP6	CENPM	CKS1B	NUCKS1
RPS2	RPL29	TPT1	SRP9	AC004990.1	ADAMTS7	TMSB10	PPP1R14B	SHH	CKLF	CCNB1	CDKN2C
RPL10	COMMD6	DYNLL1	SNRPD3	AC016831.5	COL1A2	LGALS1	GLUL	MT-CO3	MT-CYB	LGALS1	TMPO
RPS7	RPL22	ATP5F1E	OPTN	PLEKHH2	GPATCH8	PLOD2	ACTB	DEK	CENPH	PBK	CAVIN3
RPL11	RPS3A	MSRB2	NDUFA5	KCNQ1OT1	PCSK7	IFITM3	TPI1	C2orf40	PPM1G	TUBA1C	NMU
RPL24	RPL6	H3F3A	IFI35	ACAN	CDK13	ID3	ACTG1	PTMA	AP2S1	TPX2	SGO2
RPS27A	ZFAS1	MYL12A	PSMC5	IFRD1	RIC3	SELENOP	TSC22D1	MCM4	MYL6	H2AFV	HJURP
CST3	CD164	PRDX1	SUB1	AHI1	RNMT	NDUFA4L2	LMNA	DHFR	CYBA	DTYMK	TROAP
RPS9	RPS23	ATP5MD	RHOA	ITGA3	DDX3X	PTMA	ISG15	COL2A1	GMNN	ANLN	CENPA
SLC25A6	ATP5MC2	NDUFB2	UBC	POLR2J3.1	ARIH1	CYP1B1	SLC16A3	LSM4	RAN	CALM2	CCDC34
RPS14	RPL14	NDUFA4	NME1	CCNL2	ATP6V0A1	SPP1	TPM2	PFN1	ATP5MC1	TUBB4B	LSM5
RPL18	CEBPD	KRT19	ANP32A	SRSF2	ANXA1	SOX4	ERRFI1	MT-CO2	EPYC	NUSAP1	SKA2
RPL37A	RPL28	ARPC3	C9orf16	DST	LENG8	GADD45A	PLTP	TGFA	TMEM97	AL353751.1	HDGF
RPS15	RPS21	C2orf40	NDUFS6	LUC7L3	HNRNPA2B1	MDK	CD151	NUDT1	DCTPP1	MAD2L1	CFL1
RPLP2	PRELP	ESYT2	NDUFB8	ARGLU1	SEC31A	ANGPTL4	VMP1	DNMT1	HAPLN1	PTN	UBB
RPL18A	EEF1A1	GTF3C6	RPA3	AEBP1	BRD2	ANXA2	MT1X	HNRNPAB	TF	PCLAF	RPA3
RPL36A	TOMM7	GNAS	NDUFC1	SLC25A37	FOSB	TREM1	TNFRSF12A	MCM5	SIVA1	PTMA	DDX39A
RPS27	RPL27	S100A1	TAX1BP1	N4BP2L2	ST5	ZFP36L2	CDKN1A	TUBB	COPZ2	UBE2T	DYNLL1
RPL36	BTF3	HSP90AA1	ATOX1	HES1	TM4SF1	CTHRC1	DAB2	CKS1B	NASP	RAN	ANXA2
RPL7A	NOP53	ARPC1B	MTPN	MT-ND5	FAM118A	PRSS23	C1S	LAMA4	UBE2T	KPNA2	HMGB1
RPL12	CUTA	PSMC1	BZW1	THUMPD3-AS1	LONP2	SOD2	EGLN3	RARRES2	PLAC9	CDKN3	CKAP2
RPS10	C19orf33	CCT3	DNAJC21	FN1	MRC2	TM4SF1	AKR1C1	DTYMK	PAQR4	TMEM106C	DCTN3
RPS18	NUPR1	S100A11	SRGN	SF3B1	SEC24D	TGM2	ANXA1	CENPK	PLOD3	CALM3	HMMR
RPL13	ZFP36	UQCRB	PRDX3	SNRNP70	HPS4	SCG2	LY6E	E2F1	TFPI2	SMC4	COX20
RPL15	RPS20	ISG15	PPIA	SLC38A2	OGT	S100A16	MIF	PGP	MRGPRX3	RRM2	CEP55
RPL26	ATRAID	SNCG	HMGN3	SRSF11	DOCK5	TIMP1	EIF4EBP1	IGFBP2	CSPG4	CDCA3	RUVBL2
EEF1D	CCNI	CBR1	CHCHD2	ACADVL	PPP1R12B	IFITM2	IFIT3	RANBP1	NAA38	CCNB2	ARHGAP11A
RPL10A	KRT13	KRT13	CAPNS1	IRF1	GAS6	PLAUR	FNIP2	GAPDH	SCARA5	PRC1	RANBP1
RPL27A	RPL21	CAVIN2	NUCKS1	ABL2	PILRB	CCDC80	GSN	AKR1B10	PSMC3IP	NUCB2	MIF
RPL17	C1QTNF3	RHEB	ATP5PF	GABPB1-AS1	ASAP2	CP	SULF1	TMEM106C	ARPC1B	CDC20	RHEB
RPL35	RPL38	SH3BGRL	PSME2	SLC20A1	RBM39	C1R	HILPDA	ATAD2	RNASE1	CENPW	PIMREG
CD99	RPS12	NDUFAB1	ETV1	LMNA	ATF6B	IGFBP6	CDKN2A	PARP1	NDUFB2	ASPM	CENPN
FXYD1	RNASE1	ATP5F1C	AP2S1	RND3	AAK1	MXRA8	FTL	SNRPB	PTN	RPL39L	PHF19
RPL7	A1BG	UFC1	NSA2	SRSF10	ZNF83	TAGLN2	FABP5	KRT18	TCF19	PTMS	MXD3
