# Supplementary material for: Single-cell transcriptome reveals cellular hierarchies and guides p-EMT-targeted trial in skull base chordoma
Source: Cell Discov. 2022 Sep 20;8:94. doi: 10.1038/s41421-022-00459-2 (PMC9489773; doi:10.1038/s41421-022-00459-2)
Supplement: Supplementary file 17 — Supplemental Tab S7 [file 41421_2022_459_MOESM17_ESM.pdf]

Supplementary Table 7. Bulk RNA-seq identified significant changed genes in UM-Chor1 after in vitro 64 Gy radiation and two-day culture.

UP			DOWN				
Gene 1-50	Gene 51-100	Gene 101-118	Gene 1-50	Gene 51-100	Gene 101-150	Gene 151-200	Gene 201-220
CEACAMP10	PLEKHA7	AC010547.2	KNTC1	NEIL3	SPAG5	KIF20B	ASPM
HIST1H2AC	ANKRD24	TMEM106A	TACC3	ZNF395	NCAPD2	PTTG1	CIT
HIST1H4I	PDCD4-AS1	RARRES2	NDC1	AURKA	AURKB	SMARCA5-AS1	TRIB3
HIST1H1C	AC006262.2	PRODH	DEPDC1B	FANCI	SPC25	FAM83D	MKI67
HIST1H2BK	GPR158	ZNF552	TMPO	SLC4A8	SHCBP1	PRR11	DLGAP5
HIST1H2BD	LINC02482	AKR1B10	MTMR11	DHRS13	PTMAP5	SEMA6B	PIMREG
HIST1H4H	AC002401.4	PMS2P10	NEURL1B	P2RY1	SKA1	SHMT2	RNU6-1
HIST1H2BC	IL1B	AC129507.1	RGMB-AS1	PARPBP	AC026740.1	PBK	CENPF
KRT34	FAM19A5	AC139100.2	ATAD5	AC108463.1	CIP2A	DNAJC19P5	PIF1
ASTN1	RHCE	CDK5RAP3	SLCO4A1-AS1	SDAD1P1	CBS	BUB1B	DHFRP1
AL139158.2	PCSK6	LINC01534	CENPU	CHTF18	BEST1	DBF4B	AL031283.2
PI3	MRPS24	EPHX2	SAP30	ESCO2	C1QL4	TPI1P1	HIST1H1E
SPINK1	ADGRD1	RMDN2	FOSL1	UBE2SP1	SGO1	BUB1	LINC01320
HIST1H3B	TP53I3	DDO	RRM2	DEPDC1	TK1	CENPE	AC069113.3
TNFSF15	ITIH6	LYNX1	NSD2	POLQ	TM4SF18	NUSAP1	RF00003
PSG1	PSD4	AL139220.2	PSRC1	PPFIA4	ZNF887P	CDC20	TROAP
SLC9A3-AS1	AL390198.1	CPA4	AC010186.2	NCAPG2	AC091057.1	CDCA3	SREBF1
MMP28	SORCS3	TMEM217	FANCD2	ADM2	TMPO-AS1	F13A1	SERPINA3
COL17A1	TMEM198B		POC1A	AP005233.2	ZNF362	BIRC5	AP000892.3
HIST1H2BJ	AC093909.1		NCAPH	DOK3	KIF11	AC091057.6	HIST2H2AC
NEURL3	DOCK4		PKDCC	CENPM	THOC6	SPC24	
ACHE	UBE2QL1		CCDC150	KIF18B	TPX2	H19	
AC018738.1	PBLD		MELK	HMGN2	CDCA2	CKAP2L	
SLC9A3	MRAS		MND1	SLC7A5	CCDC141	IQGAP3	
EBI3	PODNL1		DHCR7	FAM72D	RAD54L	KNL1	
IFI27	AC017104.1		AC102945.2	KIFC1	SMC4	CDKN3	
RGS16	CHST4		C19orf57	SRP9P1	RPS7P10	NUF2	
LGALS3	AL049767.1		C18orf54	CCDC18	HJURP	TOP2A	
IL32	AC020928.1		TTN	ERCC6L	CCNB2	MYBL2	
ADAMTS17	TMEM150A		BORA	ATP8B3	LMNB1	GTSE1	
C11orf96	CTSL		MIS18BP1	SMC2	LRRC75A	AC068831.7	
MUC12	AKR1B15		KIF22	INSIG1	ESPL1	MXD3	
KCNB1	CPNE7		PHF19	ASNS	NCAPG	LINC01293	
HSD17B7P2	ZNF554		MAD2L1	OPRD1	PLK4	GLIPR1	
APOE	AC234775.3		RECQL4	HMGN2P5	SKA3	ANLN	
CEACAM1	AC139491.2		HASPIN	KIF18A	CEP55	KIF20A	
CCNT2-AS1	VSIR		CENPN	KIF2C	TTK	MMP3	
HIST2H2BE	COL9A1		BLM	AC069499.1	AC099850.3	SAPCD2	
CEACAM6	GSTM2		PARD6A	CENPH	NDC80	AC005224.4	
AC022034.1	CEMIP		NDUFA4L2	CCNA2	FHAD1	LINC01291	
MIR3648-1	ABHD11-AS1		HAS2	HOXA2	CCNB1	AC093001.1	
PLA2G4C	CYGB		OIP5	HMGB2	PCK2	FOXM1	
AC007448.4	HLA-DMA		KHK	CDCA8	KIF4A	KIF15	
LIPH	CPLX1		GGH	NUDT1	HMGN2P3	PLK1	
HIST1H2BN	AKR1B10P1		DHFR	C17orf49	NRGN	SPAG4	
GLYAT	CPZ		ARHGEF39	CHAC1	DPYSL4	SLC24A3	
PKD1L2	DACT1		FADS2	PLEKHA4	AC023355.1	KIF14	
AC063952.1	VSTM2L		LMNB2	CDKN2C	ARHGAP11A	CENPA	
AC142381.3	LOXL4		TRIP13	HMMR	NEK2	GPR146	
MMP19	NSUN5P2		RAD51AP1	APOBEC3B	SGO2	MBOAT1	
