# Supplementary material for: Single-cell transcriptome reveals cellular hierarchies and guides p-EMT-targeted trial in skull base chordoma
Source: Cell Discov. 2022 Sep 20;8:94. doi: 10.1038/s41421-022-00459-2 (PMC9489773; doi:10.1038/s41421-022-00459-2)
Supplement: Supplementary file 18 — Supplemental Tab S8 [file 41421_2022_459_MOESM18_ESM.pdf]

Supplementary Table 8. Three gene expression modules were identified based on up-regulating genes after in vitro radiation in UM-Chor1.

Module1	Module2	Module3
IL32	MRPS24	CEACAM6
COL17A1	SLC9A3	ITIH6
ACHE	CEACAM1	RARRES2
PRODH	ANKRD24	TP53I3
CEMIP	PLA2G4C	HIST1H2BJ
EBI3	VSIR	LGALS3
PBLD	CDK5RAP3	PCSK6
PI3	CPZ	CHST4
IL1B	ADGRD1	GLYAT
CPA4	COL9A1	ASTN1
APOE	RMDN2	NEURL3
PODNL1	EPHX2	PLEKHA7
VSTM2L	MMP19	HIST1H2BK
MRAS	PSD4	AKR1B10
CYGB	DOCK4	PDCD4-AS1
SPINK1	CTSL	DDO
IFI27	LOXL4	HLA-DMA
HIST1H2AC	ADAMTS17	UBE2QL1
HIST1H2BC	RGS16	FAM19A5
C11orf96	GPR158	CCNT2-AS1
HIST1H1C	SORCS3	LINC01534
MUC12	HIST1H2BD	AL139220.2
AL139158.2	HIST1H4H	AC022034.1
MMP28	KCNB1	LINC02482
	LIPH	AC020928.1
	DACT1	AC006262.2
	TMEM150A	HIST1H3B
	CPLX1	HIST1H4I
	ZNF554	AC007448.4
	TMEM217	
	CPNE7	
	ZNF552	
	LYNX1	
	TNFSF15	
	HIST2H2BE	
	TMEM106A	
	RHCE	
	GSTM2	
	AKR1B15	
	HIST1H2BN	
	AC002401.4	
