# Supplementary material for: Single-cell transcriptome reveals cellular hierarchies and guides p-EMT-targeted trial in skull base chordoma
Source: Cell Discov. 2022 Sep 20;8:94. doi: 10.1038/s41421-022-00459-2 (PMC9489773; doi:10.1038/s41421-022-00459-2)
Supplement: Supplementary file 20 — Supplemental Tab S10 [file 41421_2022_459_MOESM20_ESM.pdf]

Supplementary Table 10. Top 100 markers of eight modules identified by NMF in malignant cells of SBC.

RIBOSOME		OXIDATIVE PHOSPHORYLATION		CELL_CYCLE		IMMUNITY RESPONSE		ENDOPLASMIC RETICULUM STRESS		RNA SPLICING		STRESS		p-EMT	
Genes 1-50	Genes 51-100	Genes 1-50	Genes 51-100	Genes 1-50	Genes 51-100	Genes 1-50	Genes 51-100	Genes 1-50	Genes 51-100	Genes 1-50	Genes 51-100	Genes 1-50	Genes 51-100	Genes 1-50	Genes 51-100
RPS8	RPS27	S100A1	LINC01291	SERPINE2	NUDT1	IGHG3	TMEM59	SPP1	PRELP	MALAT1	SLC25A37	CXCL2	MYADM	NNMT	CEBPD
RPS6	RPL13A	SNRPG	MRPS21	SERPINE1	CYTOR	C1QA	OSTC	C3	TMED9	NEAT1	SF3B1	ANGPTL4	NNMT	IGFBP7	COL1A1
RPL26	RPL18A	SCRG1	NDUFA13	RRAD	TMSB4X	LY96	DNAJB9	HLA-B	C1QTNF3	AC020916.	GABPB1-A	ADM	MAFF	CHI3L1	MDM2
RPL5	RPS16	COX6A1	TXNDC17	FTH1	PTMA	IGHG1	EXOC1L	PDGFRL	BSG	NCL	UGP2	SOCS3	PLK3	CTSC	JUNB
RPS23	RPL39	S100A13	ATP5MC1	NDUFA4L2	LDHA	LRRC75A	CAV1	SEC62	CYBA	SLC38A2	ABCA1	JUN	BRD2	CYP1B1	RGS2
RPS4Y1	RPL32	LAMTOR5	MYL6	PLOD2	ARL6IP1	POMP	SLPI	COL2A1	TGFBI	XIST	SEC31A	HILPDA	CYTOR	PCSK2	SERPINE2
RPL30	RPL37A	CRYAB	MRPL20	PTN	COX8A	SPCS2	HLA-DPA1	HLA-A	GUSB	GOLGA8B	TPM1	FOSB	NR4A1	MT2A	TKT
STON1-G	RPS9	SEM1	ATP5MF	TGFBI	TUBB	SRGN	LAPTM4A	TF	PRSS23	HMGB1	MIR99AHG	ZFP36	GADD45A	FTH1	RND3
RPS3	RPL3	DMAC1	ATP5ME	H2AFZ	CCND1	KRT17	EIF3E	HLA-C	CD151	HSF4	ANXA1	JUNB	HSPA1A	SPP1	CYP27A1
RPS3A	RPL15	IFI27L2	SERF2	PLAUR	CYBA	HLA-DPB1	SNRPB2	COL5A2	CTSD	QTRT1	AAK1	RGS16	ATP6V0A1	SOD2	CDC42EP3
RPL7A	EEF1A1	AL353751.	BEX3	STMN1	FXYD5	NAMPT	ATP5MPL	LY96	COL11A1	CDK5RAP3	FTX	SERPINB1	SGK1	IFITM3	H3F3A
RPS14	RPL36A	LAMTOR4	NDUFA3	CCDC80	CD151	BPIFB1	SKAP2	C1S	TMCO1	PLEKHH2	NARF	CEBPD	TUBB4B	MT1X	AKR1B1
RPL13	RPL29	POLR2J	POMP	PTTG1	MGLL	SEC61G	RPL7	HTRA1	PRDX4	WSB1	RORA	AC020916.	ID2	CXCL2	PKM
EEF1B2	RPL41	H3F3A	NME2	UBE2S	RCN3	LSM5	LITAF	CLU	HLA-F	SRSF2	ARGLU1	TNFRSF12	TM4SF1	MDK	ANGPTL2
RPL12	TPT1	ZSCAN16-	COX6C	PGK1	CALD1	TMBIM4	PSMA1	HEXA	KRTCAP2	AC058791.	UTRN	HEXIM1	NABP1	HTRA1	TMEM123
RPS4X	RPS17	S100B	CAPG	TNFRSF12	HIST1H4C	MGST1	ARL6IP1	IGFBP7	ASAH1	SRRM1	ZMAT1	IER2	PPP1R3C	UCHL1	GLIPR1
RPL10	RPL22	ATP5IF1	GSTP1	CRYAB	ACTN4	RPL34	SELENOT	COL1A2	CA4	DDX5	TPR	HES1	HSPA1B	ANGPTL4	TMSB4X
RPL31	RACK1	UBL5	COX17	TUBA1B	H2AFV	CXCL8	UGP2	C2orf40	ITM2B	SON	VMP1	NEU1	RAB20	GADD45A	RRAD
RPS20	RPS28	MRPL33	COX7A2	THY1	PGP	PYURF	YWHAZ	GPNMB	SSR4	AHI1	N4BP2L2	IRF1	CYR61	GBP2	NUPR1
RPS13	RPL36	TRMT112	ATP5PF	S100A10	CD63	BPIFA1	SPCS1	SLPI	HSP90B1	FAM133B	YBX3	GADD45B	MYC	TUBA1A	CTSL
RPS18	RPL35	COX5B	NDUFB2	AL353751.	CKS1B	PPIG	TMCO1	LY6E	MYDGF	DDX18	FAM118A	KLF2	TAGLN	APOE	GLUL
RPL34	RPS24	COX7A1	ATP5F1E	LGALS1	H3F3A	CALM2	APOD	SDC2	MESD	ABI2	SRSF11	KLF4	IGFBP2	MGST1	PHLDA1
RPL19	RPL27	SRP14	HIGD2A	IFI6	TUBA1C	SFT2D1	HLA-DRB1	HAPLN1	THY1	IFRD1	SNRNP70	FOS	AMOTL2	CHI3L2	CFH
RPL11	RPS21	TXN	ARPC1B	TXN	NUCB2	HLA-DRA	SUB1	HEXB	CTSC	CCNL2	HNRNPH1	EMP1	ACTN4	SOX4	CDKN2A
RPL10A	RPL28	UQCRQ	CALM1	SLC16A3	MYDGF	PSMA2	ERH	CD320	CD99	HNRNPDL	RSRP1	NFE2L2	RHOB	ARID5B	NFKBIA
RPL9	NOP53	MTLN	S100A10	IGFBP6	KDELR2	H3F3B	CD47	RARRES2	LMAN2	RAB3B	HNRNPA2	ANXA1	IFRD1	HILPDA	FN1
RPL23A	RPS19	NDUFB1	S100A11	ANGPTL4	TAGLN2	TMEM60	UBE2B	CD164	TIMP1	DST	ITGA3	KLF5	ZC3H12A	EFNA5	SDCBP
RPS15A	COMMD6	NDUFA1	NDUFAF8	CTSB	VIM	IGLC3	CAPZA2	CTSL	FMOD	NABP1	BRD9	ATF3	NT5DC3	ID3	PLIN2
ZFAS1	RPL38	SNU13	COX8A	S100A6	CRNDE	TMEM167	TM2D1	PCSK2	MRGPRX3	TCF25	MCOLN3	DUSP1	FGL2	C3	CYR61
RPS12	RPLP2	OST4	NDUFB4	TUBA1A	TPM4	RSL24D1	C1QB	EFEMP2	LCN1	DDIT3	THUMPD3	CTGF	C11orf96	C1R	S100A6
RPS25	RPS26	GTF2H5	UQCR10	PLIN2	CALU	SUMO1	CD9	MFGE8	BCAP31	STAT3	ZRANB2	KLF6	SRSF7	VMP1	NPC2
RPS10	RPL4	HSBP1	EXOC1L	RTN4	S100A16	RPS27L	RTN4	LAPTM4A	IFI6	MEG3	CCNL1	PER2	PPP1R15A	CTSB	RARRES3
RPL21	RPS15	COX7B	NDUFA2	P4HB	GAPDH	GJA1	SRP9	GJA1	TMED10	PNISR	TRA2B	BHLHE40	TES	COL1A2	GDF15
RPS2	RPS11	RPS26	HOPX	ANXA2	GUK1	B2M	MYL12A	APOL1	PDIA3	POLR2J3.1	MUC20-O	HLA-E	YWHAH	SLPI	MSMP
RPL35A	RPL27A	RPS27L	NAGK	EIF4EBP1	KRT8	MGP	IGLC2	PPT1	NDFIP1	LINC00513	IRF1	IER3	SGMS2	GPNMB	LDHB
RPL7	FAU	NDUFS5	C19orf33	HMGB2	MMP3	TMEM50A	ZFP36L1	RCN1	DPP7	AC004990.	RBM39	JUND	PLAUR	PTMA	ZFP36L1
RPS7	BTF3	POLR2L	COX6B1	EMP3	UCHL1	PCBP1	C4orf3	CFH	ATP6V0B	LINC01116	TBXT	ARID5B	CAVIN1	TGFBI	MTHFD2
RPS27A	SLC25A6	NDUFA4	SELENOH	ACTB	ACTG1	SDCBP	RPA3	APOE	AEBP1	CDC37	DSP	RAPH1	MIR4435-	ZFP36L2	SH3BGRL3
RPS5	UQCRH	ADIRF	NDUFC1	CD44	ITGB1	CCL3L1	HMGN3	EPYC	TMBIM4	KCNQ1OT	RNMT	EGR1	ID3	IFITM2	PRSS23
RPS29	NDUFA4L2	SOD1	SNCG	SH3BGRL3	JPT1	YPEL5	TMEM70	LGALS3BP	RPN2	SBDS	EIF3A	TUBA1A	CCNL1	MT1E	CHMP4B
RPL24	RPSA	CAPZA2	COPS9	PRSS23	PFN1	TMED2	AZIN1	CD63	TMBIM6	DDX17	HP1BP3	ZEB2	PHLDA1	CDKN1A	HMOX1
RPL8	RPLP1	MINOS1	LGALS1	TIMP1	RAN	MARCKS	TMEM14A	FCGRT	CPQ	LYST	SRSF10	TRIB1	ITGA5	GSN	RBPMS
RPLP0	EEF1D	HSPE1	SDHB	HMGN2	TUBB4B	ATP6V1G1	CNIH4	HSPA1A	TMEM205	RHOT2	SYNE1	LMNA	DDX21	HIF1A	FAM20C
NACA	SNHG8	NAA38	ATP5PD	KRT18	TALDO1	SF3B6	RAB1A	TMEM59	HSPA5	SRSF5	TRA2A	NFKBIA	ZFP36L1	CXCL1	SQSTM1
RPL18	EPB41L4A	ATP5MD	VAMP5	TMSB10	MIF	SCGB1A1	IGHA1	LAMP1	IGFBP6	ACADVL	GOLGB1	CHD1	UBE2S	C1S	IGFBP6
RPL6	PFDN5	SKP1	NDUFS6	CD59	UBB	MORF4L2	EIF4A2	MGST1	CALR	LUC7L3	UPF2	RORA	KLHL21	MALAT1	PKIG
RPL14	EIF3E	PTRHD1	TMA7	MCM7	CLIC1	VPS29	WFDC2	IGFBP2	OLFML2A	FUS	TTC14	AL118516.	ZNF331	SELENOP	SOD3
RPL23	EIF3H	ATP5MC3	ROMO1	HSP90B1	PRDX4	EIF5B	MEAF6	MGARP	CTSA	CLEC18A	WDR60	BTG2	EZR	MYC	APOD
RPL37	EIF3L	NDUFB3	CA3	DTYMK	COX20	COX7A2L	RPL39	B2M	ACAN	AKAP9	EPB41L2	PPP1R12B	TMEM173	DAB2	LY96
RPL17	EEF2	TMSB4X	UQCR11	S100A11	CD24	AP3S1	SNHG25	SCRG1	SRGN	AC016831.	LRRFIP1	ABL2	H2AFX	FTL	KCNMB4
