# Supplementary material for: Single-cell transcriptome reveals cellular hierarchies and guides p-EMT-targeted trial in skull base chordoma
Source: Cell Discov. 2022 Sep 20;8:94. doi: 10.1038/s41421-022-00459-2 (PMC9489773; doi:10.1038/s41421-022-00459-2)
Supplement: Supplementary file 21 — Supplemental Tab S11 [file 41421_2022_459_MOESM21_ESM.pdf]

Supplementary Table 11. The clinical data summary of 187 SBC patients.

	Overall N = 187	p-EMT^{High} (N = 94)	p-EMT^{Low} (N = 93)	p
Gender (female)	90 (48.1%)	46 (49.5%)	44 (46.8%)	0.828
Age (years old)	45.3 (14.5)	46.51 (13.5)	44.10 (15.4)	0.256
Duration (months)	3.0 [1.0, 9.0]	3.00 [1.0, 9.0]	3.00 [1.0, 8.5]	0.805
Symptoms				
Visual loss	72 (38.5%)	36 (38.7%)	36 (38.3%)	1.000
Oculomotor paralysis	64 (34.2%)	29 (31.2%)	35 (37.2%)	0.473
Headache	51 (27.3%)	27 (29.0%)	24 (25.5%)	0.709
Dysphagia	18 (9.6%)	12 (12.9%)	6 (6.4%)	0.206
Facial numbness	12 (6.4%)	5 (5.4%)	7 (7.4%)	0.780
Hoarseness	11 (5.9%)	8 (8.6%)	3 (3.2%)	0.207
Walking instability	9 (4.8%)	3 (3.2%)	6 (6.4%)	0.505
Hearing loss/tinnitus	5 (2.7%)	2 (2.2%)	3 (3.2%)	1.000
Surgical history	67 (35.8%)	38 (40.9%)	29 (30.9%)	0.202
Surgical approach (%)				0.428
Transcranial	51 (27.3%)	29 (31.2%)	22 (23.4%)	
Transsphenoidal	136 (72.7%)	64 (68.8%)	72 (76.6%)	
Resection extend				
Total	60 (29.4%)	30 (32.6%)	23 (26.1%)	0.430
Subtotal	127 (70.6%)	63 (67.4%)	71 (73.9%)	
Radiotherapy after surgery				0.429
No	119 (63.6%)	62 (66.7%)	57 (60.6%)	
Conventional radiotherapy	27 (14.4%)	15 (16.1%)	12 (12.8%)	
Cyberknife/Gamaknife	21 (11.2%)	9 (9.7%)	12 (12.8%)	
Proton beam	20 (10.7%)	7 (7.5%)	13 (13.8%)	
