# Supplementary material for: Single-cell transcriptome reveals cellular hierarchies and guides p-EMT-targeted trial in skull base chordoma
Source: Cell Discov. 2022 Sep 20;8:94. doi: 10.1038/s41421-022-00459-2 (PMC9489773; doi:10.1038/s41421-022-00459-2)
Supplement: Supplementary file 22 — Supplemental Tab S12 [file 41421_2022_459_MOESM22_ESM.pdf]

Supplementary Table 12. Summary of phase I clinical trial of YL-13027 in three SBC patients.

Section/Topic	Item
**Title**	Single-arm, Open-Label, Single and Multiple-Dose Escalation Phase I Clinical Study to Evaluate the Tolerability, Pharmacokinetics and Pharmacodynamics of YL-13027 in Patients with Advanced Solid Tumors
**Methods**	
Trial design	This is a single-arm, open-label, single and multiple-dose escalation Phase I clinical study. It is intended to enroll about 19-36 subjects. There are YL-13027 60 mg/d, 120 mg/d, 180 mg/d, 240 mg/d, 300 mg/d, and 360 mg/d groups, and a consecutive 28-day dose regimen is applied. One subject will be enrolled in 60 mg/d and 120 mg/d groups, respectively, and 3-6 subjects will be enrolled in 120 mg/d above groups, respectively. During the multiple-dose phase, route of administration: oral, twice a day.<br>This study includes two phases: dose escalation and dose expansion; each phase includes single-dose and multiple-dose studies. The dose expansion test can be carried out since one case of PR / CR or two cases of SD ≥ 8 weeks occurred in any dose group after discussion and confirmation between the investigator and the sponsor. The dose expansion test can be extended to 8-12 subjects to further evaluate the safety, preliminary efficacy and pharmacokinetics study.
Participant(s)	**Patients meeting all of the following inclusion criteria can be enrolled in this trial:**<br>1) Patients who are 18-75 years old (including cut point), regardless of gender;<br>2) Patients with advanced malignant solid tumors diagnosed by histology or cytology who have failed in standard of care, or have not receive the standard of care, or are not suitable for standard of care at this phase as determined by the investigator;<br>3) In the dose escalation part, according to the RECIST1.1 criteria, both measurable and non-measurable tumor lesions are acceptable; in the dose expansion part, there is at least one measurable tumor lesion. ECOG performance status (PS) is Grade 0-1;<br>4) Expected survival ≥ 3 months;<br>5) With good organ function level:<br>a) Neutrophil count (ANC) ≥1.5×109/L;<br>b) Platelet count (PLT) ≥100×109/L;<br>c) Hemoglobin (Hb) ≥90 g/L (no blood transfusion within 14 days before screening test);<br>d) Total bilirubin (TBIL) ≤ 1.5 times the upper limit of normal;<br>e) Alanine aminotransferase (ALT) and aspartate aminotransferase (AST) ≤2.5 times the upper limit of normal (for patients with liver metastases, ≤5.0 times the upper limit of normal);<br>f) Serum creatinine (Cr) ≤1.5 times the upper limit of normal or creatinine clearance ≥50 ml/min;<br>g) Left ventricular ejection fraction (LVEF) ≥50%;<br>h) QT interval corrected using Fridericia (QTcF) ＜ 450 ms.<br>6) The washout phase from the end of the prior anti-tumor therapy (including chemotherapy, biological therapy, endocrine therapy, targeted therapy, immunotherapy, tumor embolization or Chinese medicine with anti-tumor indications) to the first dosing of the study drug is ≥ 3 weeks, of which the washout phase for oral fluorouracils and small-molecule targeted drugs is ≥2 weeks, the washout phase for mitomycin C

and nitrosourea is ≥6 weeks, and the washout phase for other anti-tumor treatments (radiotherapy, treatment with other investigational drugs) is ≥4 weeks;
7) Male and female patients with childbearing potential must agree to take medically approved birth control during the study and within 6 months after the last dosing of the study drug;
8) Female patients with childbearing potential: The blood pregnancy test result is negative within 7 days before the first dosing of the study drug. The patient cannot breastfeed; if the subject has stopped breastfeeding when entering the study, then breastfeeding should be stopped from the day starting giving the study until more than 30 days after the last dosing;
9) Patients who have not received the investigational drug treatment within 1 month before participating in this trial;
10) Patients who have high compliance judged by the investigator, and are willing to complete the test and able to comply with the study protocol;
11) Voluntarily to participate in this clinical investigation, understand the study procedure and be able to sign ICF.

Patients meeting any one of the following criteria cannot be enrolled in this study:
1) Patients who have third interstitial fluid (such as a large amount of pleural fluid and ascites) which cannot be controlled as judged by the investigator;
2) Patients who have experienced Grade 3 or 4 digestive tract haemorrhage or varicose vein hemorrhage within 3 months before the first dosing and require blood transfusion or endoscopic or surgical intervention;
3) Patients with failure to swallow, chronic diarrhoea, or intestinal obstruction, and many other factors which affect the drug use and absorption;
4) Patients with confirmed history of neurological or mental disorder;
5) Patients with active hepatitis B (hepatitis B surface antigen and/or hepatitis B core antibody positive and HBV-DNA ⩾103 copies/mL or ⩾ 200 IU/mL) or hepatitis C (hepatitis C virus antibody positive and/or HCV-RNA positive);
6) Patients with medical history of immunodeficiency, including HIV test positive, or with other acquired, congenital immunodeficiency, or with history of organ transplant, or with history of allogeneic bone marrow transplant;
7) Patients with moderate or severe cardiac disorders:

a) Patients with myocardial infarction, angina pectoris, Grade III/IV congestive heart failure, pericardial effusion, and uncontrollable severe hypertension (up to 150/90 mmHg or below) within 6 months before the first dosing;
b) Patients with abnormal ECG that is of clinical significance: such as symptomatic or persistent atrial or ventricular arrhythmia, Grade II or III atrioventricular block, bundle branch block, and ventricular hypertrophy;
c) Patients whose echocardiogram shows significant abnormalities: such as moderate or severe heart valve function defects, evaluated based on the lower limit of normal specified by the test facility; patients with minimal or mild valve regurgitation (tricuspid valve, pulmonary valve, mitral valve or aortic valve);
d) Patients whose troponin T increased in laboratory tests during screening;
e) Patients with many factors that may increase the risk of QTcF prolongation or the risk of arrhythmia events: such as hypokalemia, congenital long QT syndrome, and concomitant medications that may prolong the QT interval;
f) Patients with susceptibility factors for occurrence and development of ascending aorta or aortic arch aneurysm, such as Marfan’s syndrome, and a history of cardiac vascular injury recorded by CT;
g) Patients with a history of cardiac or aortic surgery;

	8) Patients with metastasis to the central nervous system;<br>9) At the start of the study treatment, the unrecovered toxicity of the prior therapy is higher than CTCAE5.0 Grade 1 (except for alopecia);<br>10) Patients who have received the treatment with TGF-β signaling targeted drugs;<br>11) Patients with medical conditions that seriously endanger the safety of the patients or affect the completion of the study judged by the investigator, such as uncontrollable diabetes, thyroid disorder, interstitial pneumonia, severe active infection or uncontrolled chronic infection, Child- Pugh Grade Bor C cirrhosis etc.;<br>12) Subjects who are not suitable for participating in this study for other reasons at discretion of the investigator.<br>Patients with SBC were assessed and enrolled in Huashan Hospital in Shanghai, China, since Jan. 2021. This study was approved by Medical Ethics Committee of Huashan Hospital, Fudan University, Shanghai, China.
Interventions	This is a phase I open-label study. Only one group was designed. All 3 enrolled SBC patients were treated by YL-13027 in oral administration.<br>Single dose: Administrated with a total single dose under fasting condition in the morning on Day 1.<br>Multiple doses: Multiple doses: 48 hours after Day 1 of dosing, for 28 consecutive days, as a treatment cycle.<br>Patients were followed up every 4 weeks, MRI evaluation were performed every 8 weeks.<br>Resumption of treatment for patients with a dose-limiting toxicity was permitted (when clinically appropriate) if the severity of the toxicity fell to grade I or lower and treatment was interrupted for no more than 2 weeks. All adverse events were graded according to the National Cancer Institute's Common Terminology Criteria for Adverse Events (CTCAE; version 5.0).
Outcomes	Primary Objective:<br>a) To determine the safety, tolerability and Maximum Tolerated Dose (MTD) of YL-13027 tablets in patients with advanced solid tumors by single and multiple oral administrations.<br>Secondary Objectives:<br>a) To observe the pharmacokinetic profiles of YL-13027 in patients with advanced solid tumors;<br>b) To preliminarily evaluate the efficacy of YL-13027 treatment in patients with advanced solid tumors;<br>c) To explore and observe changes in biomarker indicators TGF-β1, pSMAD2/pSMAD3, total SMAD2 and SMAD3.
Sample size	All patients with chordoma who meets the eligibility criteria were enrolled after informed consent since Jan. 2021. Totally, 5 patients were assessed and 3 were enrolled.
Statistical methods	The statistical description method is mainly used in the test results. The mean, standard deviation, median, minimum, and maximum are listed for the measurement indicators, and the frequency (composition ratio) and ratio are listed in the count data and grade data. The 2-sided test is used in the statistical tests. If the P value is less than or equal to 0.05, the difference tested is considered to be statistically significant (unless otherwise specified), and the confidence interval is 95%. The plasma concentration data is used to calculate the primary PK parameters to fully reflect the drug absorption, distribution, metabolism and excretion characteristics in the human body. The primary PK parameters include Tmax, Cmax, AUC0-t, AUC0-∞, t1/2, Vd/F, CL/F, etc.

**Results**	
Participant flow	See Supplementary Fig. S10a. Two SBC patients were excluded because of difficulty in swallowing due to lower cranial neuropathy caused by SBC compression.
Recruitment	Five Patients were assessed and 3 were recruited from January to August in 2021. All recruited patients were followed up every 8 weeks. Two for 3 cycles and 1 for 2 cycles.
Baseline data	See Supplementary Table 13
Outcomes and estimation	Both of them could tolerate the 360 mg/d dose and show no SAE or DLT during follow-up. All patients reached SD during follow-up. The pharmacokinetic profiles of both patients were shown in Supplementary Fig. 10b-d.
Harms	Up to the study cutoff date, several adverse events (AEs) were observed and summarized in Supplementary Table 14, including rash and transient creatinine elevation. No serious AEs (SAEs) were observed.
**Other information**	
Registration and Protocol	https://clinicaltrials.gov/ct2/show/record/NCT03869632
