# Supplementary material for: Single-cell transcriptome reveals cellular hierarchies and guides p-EMT-targeted trial in skull base chordoma
Source: Cell Discov. 2022 Sep 20;8:94. doi: 10.1038/s41421-022-00459-2 (PMC9489773; doi:10.1038/s41421-022-00459-2)
Supplement: Supplementary file 23 — Supplemental Tab S13 [file 41421_2022_459_MOESM23_ESM.pdf]

Supplementary Table 13. Summary of Baseline date of three SBC patients enrolled in the clinical trial.

Number	Patient 1	Patient 2	Patient 3
Age (year)	50	40	58
Gender	female	male	female
Height (cm)	158	165	155
Weight (kg)	56.5	60	68
Primary tumor location	Sellar region and uppers clivus	Lower clivus and cervical junction	Uppers clivus
Date of first pathologic diagnosis	2017/07/18	2020/08/07	2017/11/05
Pathologic diagnosis	chordoma	chordoma	chordoma
Clinical diagnosis	Skull base chordoma	Skull base chordoma	Skull base chordoma
Baseline tumor maximum diameter (mm)	46	45	39.9
ECOG performance status	0-1	0-1	0-1
Acceptable organ function level	yes	yes	yes
Having received prior anti-tumor therapy before	no	no	no
Having received the investigational drug treatment within 1 month	no	no	no
With childbearing potential	no	no	no
With third interstitial fluid	no	no	no
Experienced grade 3 or 4 digestive tract haemorrhage or varicose vein hemorrhage within 3 months	no	no	no
Good function of drug absorption	yes	yes	yes
With confirmed history of neurological or mental disorder	no	no	no
With active hepatitis B or C	no	no	no
With medical history of immunodeficiency	no	no	no
With moderate or severe cardiac disorders	no	no	no
With metastasis to the central nervous system	no	no	no
Having received the treatment with TGF-β	no	no	no

signaling targeted drugs before			
With medical conditions that seriously endanger the safety of the patients or affect the completion of the study	no	no	no
