# Supplementary material for: Single-cell transcriptome reveals cellular hierarchies and guides p-EMT-targeted trial in skull base chordoma
Source: Cell Discov. 2022 Sep 20;8:94. doi: 10.1038/s41421-022-00459-2 (PMC9489773; doi:10.1038/s41421-022-00459-2)
Supplement: Supplementary file 24 — Supplemental Tab S14 [file 41421_2022_459_MOESM24_ESM.pdf]

Supplementary Table 14. Summary of Adverse Effects in phase I clinical trial of YL-13027 in three SBC patients.

Patient	AE	Starting day	Classification of AE	Relavent to YL-13027*	Duration	Intervention
Patient 1	Creatinine elevation	22	1	Possibly relevant	Transient	None
Patient 1	Rash	15	1	Possibly relevant	Persistent	Symptomatic treatment
Patient 1	Leukopenia	141	2	Possibly relevant	Persistent	None
Patient 2	Constipation	3	2	Possibly relevant	Transient	Symptomatic treatment
Patient 2	Gastric distension	3	1	Possibly relevant	Transient	Symptomatic treatment
Patient 2	Appetite reduction	3	1	Possibly relevant	Transient	None
Patient 2	Creatinine elevation	15	1	Possibly relevant	Transient	None
Patient 2	Rash	54	1	Possibly relevant	Transient	None
Patient 1	Maculopapule	1	2	Possibly not relevant	Transient	Symptomatic treatment
Patient 1	Fever	2	1	Possibly not relevant	Transient	None
Patient 1	Pharyngalgia	2	2	Possibly not relevant	Transient	None
Patient 1	Purpura	5	1	Possibly not relevant	Transient	None
Patient 1	Insomnia	3	1	Possibly not relevant	Transient	None
Patient 1	Cough	6	1	Possibly not relevant	Transient	Symptomatic treatment
Patient 1	Xerosis cutis	16	1	Possibly not relevant	Transient	Symptomatic treatment
Patient 1	Leukopenia	15	2	Possibly not relevant	Transient	None
Patient 1	Fever	18	2	Possibly not relevant	Transient	Symptomatic treatment
Patient 1	Cervical lymphadenectasis	19	2	Possibly not relevant	Transient	Symptomatic treatment
Patient 1	Gum bleeding	36	1	Possibly not relevant	Persistent	None
Patient 1	FOBT (+)	85	1	Possibly not relevant	Transient	None
Patient 1	Constipation	113	2	Possibly not relevant	Persistent	Symptomatic treatment
Patient 2	Papule	1	1	Possibly not relevant	Transient	None
Patient 2	Cough	11	1	Possibly not relevant	Transient	Symptomatic treatment
Patient 2	Fever	11	1	Possibly not relevant	Transient	None
Patient 2	FOBT (+)	29	1	Possibly not relevant	Transient	Symptomatic treatment
Patient 2	Diarrhea	56	1	Possibly not relevant	Transient	Symptomatic treatment
Patient 2	FOBT (+)	57	1	Possibly not relevant	Transient	None

Patient 2	Urine occult blood (+)	46	1	Possibly not relevant	Persistent	None
Patient 2	Urine red blood elevation	46	1	Possibly not relevant	Transient	None
Patient 2	Hypokalemia	59	1	Possibly not relevant	Transient	Symptomatic treatment
Patient 2	Acute gastroenteritis	58	3	Possibly not relevant	Transient	Stop YL-13027 administration for 2 days and symptomatic treatment
Patient 2	Urine red blood elevation	141	1	Possibly not relevant	Persistent	None
Patient 2	Urobilinogen (+)	141	1	Possibly not relevant	Transient	None
Patient 3	Urinary leukocyte elevation	7	1	Possibly not relevant	Transient	None
Patient 3	Hypokalemia	42	1	Possibly not relevant	Transient	Symptomatic treatment
Patient 3	Urinary leukocyte elevation	112	1	Possibly not relevant	Transient	None

* Relevant to YL-13027 was judged by investigators
